# Structure-based design of an aromatic helical foldamer–protein interface†

**DOI:** 10.1039/d5sc01826a

**Published:** 2025-06-02

**Authors:** Lingfei Wang, Céline Douat, Johannes Sigl, Post Sai Reddy, Lucile Fischer, Béatrice Langlois d'Estaintot, Zhiwei Liu, Vojislava Pophristic, Yuwei Yang, Yingkai Zhang, Ivan Huc

**Affiliations:** a Department Pharmazie, Ludwig-Maximilians-Universität München Butenandtstr. 5–13 81377 München Germany ivan.huc@cup.lmu.de; b CNRS, Bordeaux INP, CBMN, UMR5248, Univ. Bordeaux F-33600 Pessac France; c Department of Chemistry & Biochemistry, Rowan University 201 Mullica Hill Road 08028 Glassboro New Jersey USA; d Department of Chemistry, New York University 10003 New York New York USA

## Abstract

The starting point of this study is the solid state structure of a complex between human carbonic anhydrase II (HCAII) and a helically folded tetradecaamide aromatic foldamer with a nanomolar HCAII ligand appended at the N terminus of the helix. In this complex, the foldamer is achiral but its handedness is biased by diastereoselective interaction with the protein. Computational analysis of the HCAII surface and inspection of the initial solid state structure led to the suggestion of main chain and side chain modifications of the foldamer helix that would result in an extension of the foldamer protein interface as well as in absolute helix handedness control. Molecular dynamics simulations validated several of these suggested modifications as potentially resulting in favorable foldamer–protein contacts. Five new Fmoc-protected amino acid building blocks bearing new biogenic-like side chains were synthesized. Nine new tetradecaamide sequences with or without the appended HCAII ligand were synthesized on solid phase and purified by RP-HPLC. The solid state structures of four of these sequences in complex with HCAII were obtained and validated the main design principles: (i) side chains can be predictably introduced at precise positions of the foldamer surface to create new contacts with the protein; (ii) side chains modifications do not alter main chain behavior and can be implemented independent from each other; (iii) some main chain units derived from quinoline-, pyridine-, or benzene-based δ-amino acids are largely interchangeable without altering the overall helix curvature in the context of a complex with a protein. An assessment of the *K*_D_ values required the adaptation of an existing fluorescence competition assay and suggested that the side chain and main chain modifications introduced in the new sequences did not result in significant improvement of the affinity of the foldamers to HCA.

## Abstract 2

Computational analysis guided the functionalization of an aromatic foldamer helix with biogenic-like side chains to extend its interaction interface with a protein to which the foldamer is bound *via* a ligand.

## Graphical abstract

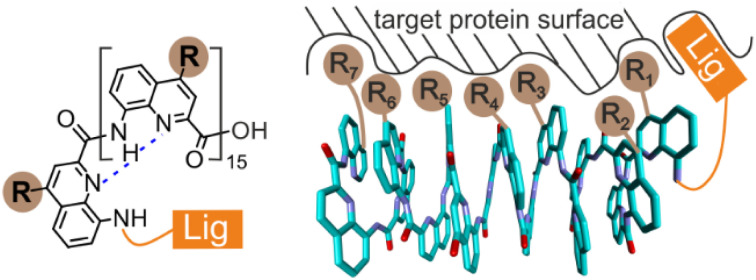

## Introduction

**Fig. 1:**
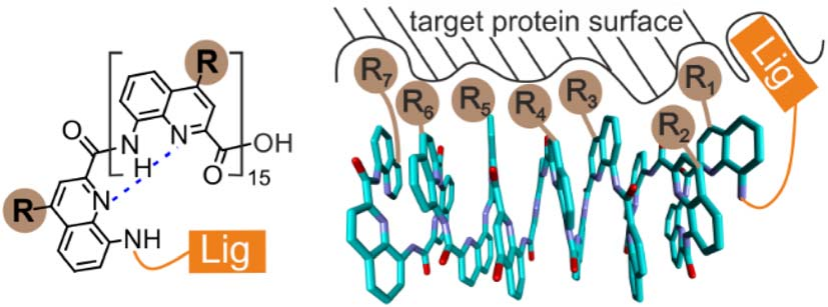
Chemical formula (left) of a hexadecaamide of 8-amino-2-quinolinecarboxylic acid bearing a protein ligand (Lig) at the N terminus and biogenic-like side chains (R groups) in position 4. Schematic representation (right) of the helical structure of the hexadecaamide with some R groups interacting with a protein surface to which the ligand is also bound.

Aromatic oligoamides represent a large class of compounds that can be used to recognize proteins and nucleic acids and that may interfere with their functions in multiple ways.^1^ They comprise natural products such as distamycin,^2^ cystobactamids,^3^ and albicidin,^4^ drug molecules such as suramin that has been crystallized bound to numerous proteins,^5^ rod-like oligomers many of which have been developed as α-helix mimetics,^6^ and oligomers that adopt helically folded conformations.^7–10^ We have been interested in the latter because their relatively large size offers the possibility to cover a large surface area of a protein target, which is relevant to protein–protein and protein–nucleic acid interactions, two types of interactions that are difficult to inhibit with small molecules.^11^ Helical aromatic oligoamide foldamers (AOFs) and in particular those derived from 8-amino-2-quinolinecarboxylic acid (Fig. 1) also possess the advantage that their conformations are very stable in particular in protic solvents,^12^ and that synthetic methods exist to introduce various biogenic-like side chains at their periphery.^13^

The potential of helical AOFs to interfere with protein function has been highlighted in the context of amyloid fibers^7^ and DNA-binding proteins.^8^ For the latter, AOFs that specifically mimic the shape of charge distribution of DNA have been developed. In contrast, methods are still missing to design *ab initio* a helical AOF protein binder that does not mimic an already known binding epitope, for example through the introduction of biogenic-like side chains complementary to the protein surface. To assess the potential of some helical AOFs to interact with a given protein surface, we introduced a tethering approach where a covalent or non-covalent linkage confines the AOF at the surface of the protein (Fig. 1).^8,9^ Tethering between two small molecules or between a small molecule and its protein target is a common approach in the context of drug research to compensate for initially weak binding. It lies at the heart of linker design in fragment-based approaches,^14^ including in the context of template-assisted strategies,^15^ and of covalent ligands.^16^ Tethering of AOFs to a protein target was used to detect foldamer–protein interactions upon observing a preferred handedness in an achiral oligomer.^8,9^ Taken alone, the achiral AOF exists as a racemic mixture of right-handed (*P*) and left-handed (*M*) enantiomeric conformers. If either helix (*M* or *P*) interacts better than the other with the protein surface, the resulting change of proportion leads to an induced circular dichroism (CD) signal. An AOF CD signal is easy to detect because AOFs absorb above 350 nm, in regions where proteins are transparent. For these studies, we have used human carbonic anhydrase II (HCAII) as a model system.^8,9^ This protein was initially selected because it is robust and commercially available as an easy-to-handle freeze-dried solid, *i.e.* it can be supplied to a chemistry laboratory without requiring recombinant protein expression. Furthermore, commercial HCAII was found to readily crystallize and small nanomolar ligands for this enzyme that can conveniently be appended to foldamers exist. Thus, strong helix handedness induction was observed with several helical AOFs linked to the protein *via* a nanomolar ligand. Subsequently, solid state structures of such complexes were obtained that confirmed the preferred *P* helix handedness and informed about foldamer protein contacts.^8,9^

**Fig. 2:**
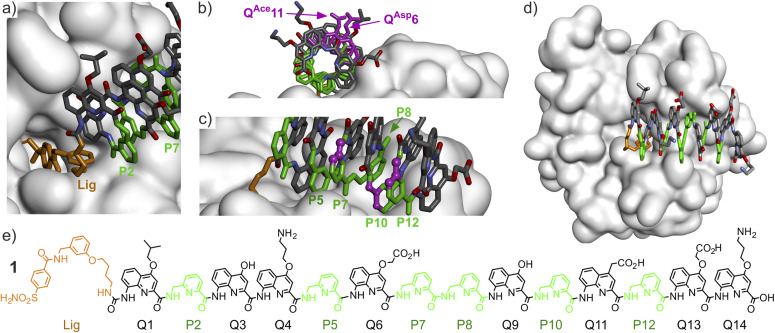
(a)–(d) Show different views of the solid state structure of the complex between HCAII and AOF 1.^9^ Only the surface of the protein is shown. The foldamer is shown in stick representation. Pyridine units (P) are shown in green and the HCAII ligand is shown in gold. In (b), two residues of interest are shown in purple. In (c) three carbon atoms of three P residues are shown in purple balls. These carbons would belong to the additional benzenic ring when implementing a P→Q mutation at these positions. Hydrogen atoms have been omitted for clarity. (e) Structural formula of 1.

In the case or tetradecamide sequence 1, the solid state structure showed a large contact surface area between the foldamer and HCAII (Fig. 2).^9^ However, it has not yet been shown whether such a structure could serve to further design the foldamer to extend its contact with the protein surface. Furthermore, although the AOFs are known to be rigid, it remained to be demonstrated whether side chain and main chain modifications could be implemented without altering their overall structure, a task difficult to achieve with *e.g.* a peptide or an aliphatic peptidic foldamer.^17^ Here, we show that, with the help of computational tools, the solid state structure of the complex 1·HCAII can be used as a starting point to place side chains at defined positions in space to further elaborate the foldamer–protein interface. We validate that the foldamer structure remains independent of side chain variations and even some main chain variations. Although the changes implemented have not resulted in significant changes in the dissociation constant of the complexes, the results further validate the concept that AOF helices can serve as reliable scaffolds to display biogenic-like side chains at the surface of a protein. The results also show that the HCAII system had some shortcomings alongside the advantages mentioned above.

## Results and discussion

### Synthesis and general design principles

**Fig. 3:**
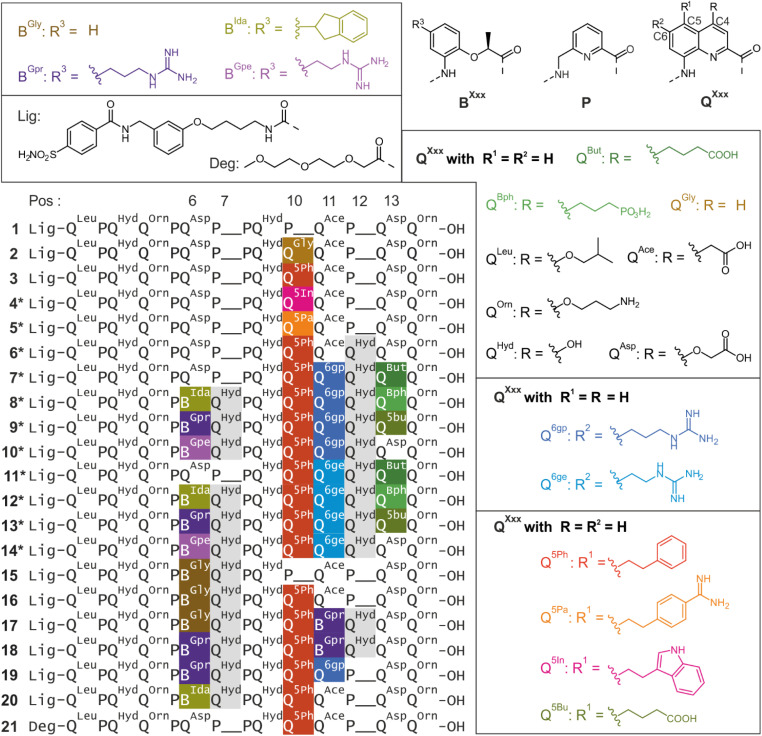
Structural formulas of P, Q^Xxx^, and B^Xxx^ δ-amino acids and of N-terminal Lig and Deg functional groups. Carbon atoms in position 4, 5, and 6 of Q^Xxx^ are indicated and carry R, R^1^, and R^2^ side chains, respectively. The Xxx three letter code used for the side chains is sometimes inspired by the three letter code of α-amino acids bearing similar side chains, even when they may not exactly match. The three letter code also indicates when the side chain is in position 5 or 6 of the quinoline ring. Sequences 1–21 are defined with the letter code used in this study. To facilitate residue identification, the colors of the highlighted residues match with the side chain colors in the adjacent boxes. Sequence numbers with a star (4*–14*) indicate sequences that were investigated in molecular dynamics simulations only and were not synthesized.

Oligoamide sequence 1 consists of eight Q^Xxx^ δ-amino acid monomers presenting different biogenic-like side chains in position 4, and of six P residues (Fig. 2e and 3). P residues bring the same contribution to helix curvatures as Q^Xxx^ but they are more flexible. Their initial role was to make helix handedness dynamics fast enough to be practically monitored, *e.g.* in the course of minutes to hours,^18^ for example when helix handedness bias takes place upon binding of 1 to HCAII.^9^ Unexpectedly, P residues were found to be directly involved in foldamer–protein contacts in the solid structure of 1·HCAII (Fig. 2b). Their role thus extends to that of interacting units despite the fact that they carry no biogenic-like side chains.

In order to extend foldamer–HCAII contacts, a number of Q^Xxx^ monomers were considered bearing side chains in position 4, 5 or 6 (Fig. 3). B^Xxx^ monomers were also involved. B^Xxx^ monomers are δ-amino acids as well and may thus bring a contribution to helix curvature similar to that of Q and P.^19^ In addition, they carry a stereogenic center that has been shown to quantitatively bias helix handedness.^20^ Sequences comprising B^Xxx^ monomers are thus designed to be one handed even when they are not bound to HCAII. The *S* configuration of B^Xxx^ monomers is intended to favor *P* helicity. Among the monomers used in this study (Fig. 3), some were previously described, some were used only in computations, and five – B^Ida^, B^Gpr^, Q^Gly^, Q^5Ph^ and Q^6gp^ – were newly synthesized (Fig. S1†). The preparation of these building blocks in a form suitable for solid phase synthesis is presented in detail in the ESI.† All monomers were produced with a free carboxylic acid and an Fmoc-protected main chain amine. In addition, the side chains of B^Gpr^ and Q^6gp^ were protected with Boc groups. Typically, side chain installation involved Sonogashira or Suzuki cross-coupling reactions on a bromoaryl precursor.^13^ The synthesis of Q^5In^ is also described in the ESI.† However, although this monomer is a stable compound in its protected form (with a Boc group on the indole ring), sequences that contain it degrade over time after deprotection, apparently through an oxidation process, and could not be investigated. A representative example of this degradation is shown in Fig. S2.† It appears that oxidation is mediated by the foldamer and possibly involves light. For instance, we found that biotinylated sequences must be protected from air and light for storage to avoid the formation of biotin oxide.^10*a*^

Sequences 1–3 and 15–21 (Fig. 3) were synthesized on solid phase using an established *in situ* acid chloride activation protocol for the coupling steps.^21^ An improved procedure for the on-resin introduction of the HCAII ligand at the N-terminus of the helix using a urea linkage was also developed. Sequences were purified by RP-HPLC after TFA-mediated resin cleavage and side chain deprotection.

Sequences 4*–14* were investigated in computational studies but not synthesized. Sequences 2–3, 4* and 5* were the focus of a first phase of our investigation. They are analogues of 1 in which P10 is replaced by different, more rigid Q10, residues. In the case of 3, it could be verified experimentally that helix handedness bias upon binding to HCAII takes place despite the added rigidity, albeit significantly slower than with 1 (Fig. S3†). This first phase led to the installation of a Q^5Ph^10 residue in sequence 3, instead of P10 in 1. Q^5Ph^10 was conserved in all subsequently synthesized sequences except in 15. In a second phase, residue variations in positions 6, 7, 11, 12 and 13 were assessed computationally in sequences 6*–14* (see next section) and a selection of these variations was experimentally implemented in 15–20. Sequence 21 is an analogue of 3 lacking the N-terminal ligand.

Including chiral B^Xxx^ units to favor *P* helix handedness was desirable, for example to avoid conformational changes in the course of a *K*_D_ value determination. For this purpose, residues 6 and 11 were chosen as possible locations. This choice was based on the observation that the side chains in position 4 of the quinoline rings of Q^Asp^6 and Q^Ace^11 of 1 lie far from the HCAII surface (Fig. 2b). Removing these side chains and part of the pyridine ring of Q when performing a Q→B mutation should not alter the HCAII–foldamer interactions observed in the solid state. In contrast, the carbon atoms in position 5 and 6 of the quinoline rings of Q^Asp^6 and Q^Ace^11 in 1 seem better oriented to introduce a side chain that may interact with the HCAII surface and a B^Xxx^ monomer may offer a similar side chain presentation.

Before implementing Q→B mutations, another design feature had to be considered. While chiral B units have been shown to quantitatively bias helix handedness in the context of (Q)_*n*_ oligomers,^20^ this has not been validated when the helix also contains multiple more flexible P units, as in 1. Indeed, partial handedness bias has occasionally been observed when Q monomers are mixed with other monomers.^22^ To mitigate the risk that the chiral B-containing sequences would not be quantitatively one handed, we replaced some P units by Q in the vicinity of the B6 and B11 monomers. With their additional fused benzenic ring, Q monomers are bulkier than P. The structure of the 1·HCAII complex showed that this extra bulk could be accommodated without generating clashes in P7, P10 and P12, but not in P5 and P8 (Fig. 2c). Chiral B-containing sequences 15–20 therefore contain at least one and sometimes up to three Q monomers at residues 7, 10 or 12.

The one-handed nature of the new chiral foldamers could be verified by ^1^H NMR spectroscopy through the observation of a single set of signals. On top of ensuring quantitative handedness bias, the additional Q residues also resulted in slow helix handedness inversion in water. In case handedness bias was incomplete when the foldamer was first dissolved in water, *e.g.* for RP-HPLC purification, it may no longer proceed to completion. This pitfall is easily detected by the observation of two distinct sets of signals on the ^1^H NMR spectra, corresponding to *P* and *M* diastereomeric conformers. To solve this problem, one can dissolve and incubate the compound in an organic solvent such as DMF, where helix handedness inversion takes place faster,^12^ before evaporating and redissolving in water.

### Computational design

#### Protein surface analysis

**Fig. 4:**
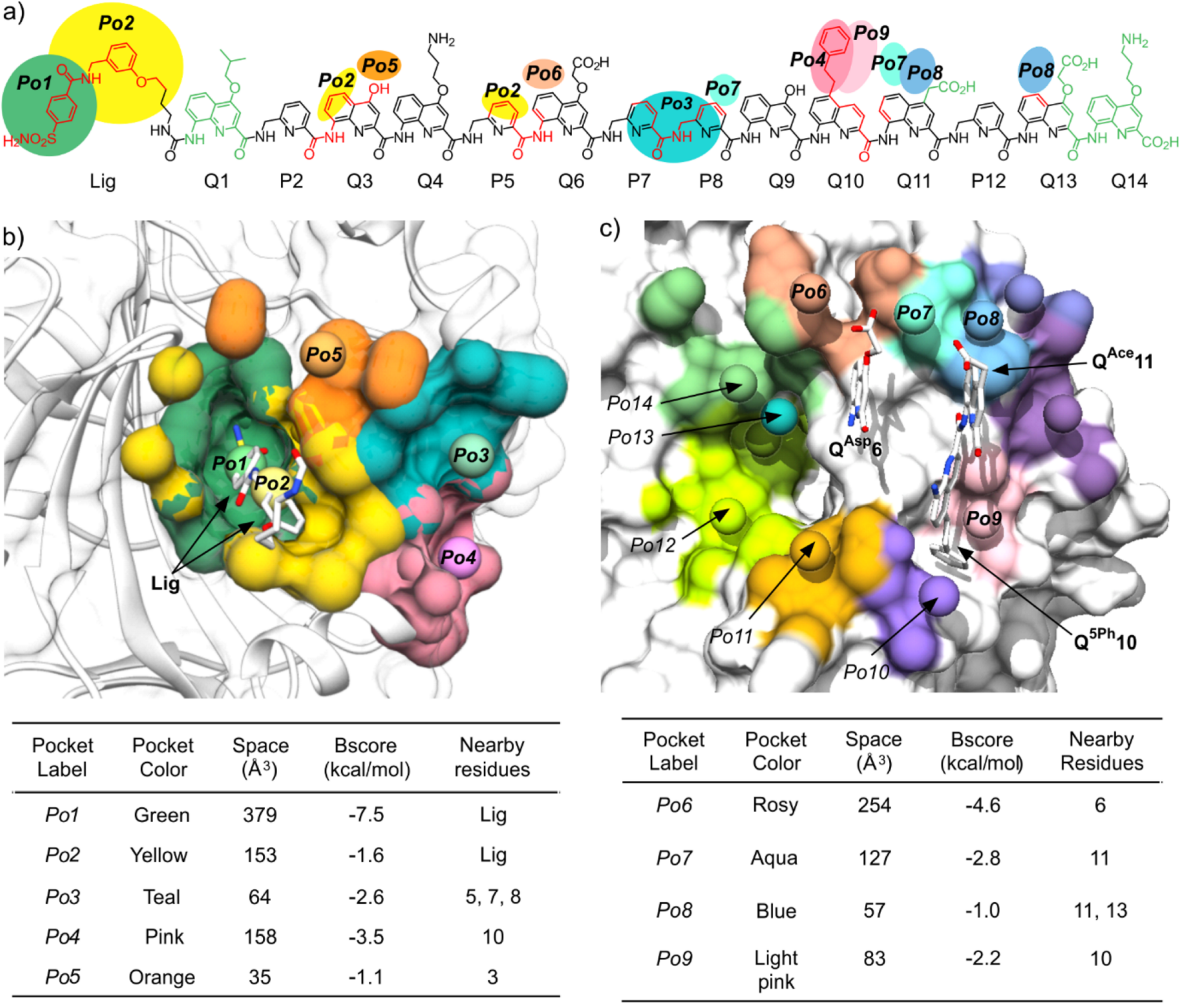
(a) Structural formula of 3 including Q and P residue numbering. The bonds shown in red highlight parts of the molecule in direct contact with the HCAII surface in the solid state structure of the 3·HCAII complex (Fig. 5). The bonds shown in green highlight parts of the foldamer involved in intercomplex contacts in the crystal lattice of the solid state structure of the 3·HCAII complex. The color patches indicate the pockets near the foldamer main chain or side chains in the solid state structure of the complex. Pockets are colored and numbered as in (b) and (c). (b) Pocket analysis of the surface of HCAII restricted to the vicinity of the ligand binding site and the contact area with the foldamer helix in the solid state structure of the 1·HCAII complex (Fig. 2). (c) Pocket analysis of the surface of HCAII restricted to the vicinity of the contact area with the foldamer helix in the solid structure of the 3·HCAII complex (Fig. 5). In (b) and (c), pockets have been assessed in terms of their volume and their ligandability (BScore). Pockets Po10-Po14 were not included in this study.

The potential of the HCAII surface for interacting with biogenic-like residues was assessed with AlphaSpace, a computational analysis tool designed for fragment-centric topographical mapping.^23^ The assessment proceeded in two phases. In a first phase, the surface in the vicinity of the HCAII active site was analysed, leading to the identification of potential binding pockets Po1–Po5 (Fig. 4b). Po1 has the highest ligandability (highest Bscore) and corresponds to the HCAII active site where HCAII ligands usually bind.^24^ In the 1·HCAII complex, Po1, Po2, Po3 and Po5 are filled by the N-terminal ligand and the helix backbone, as indicated by the color patches in Fig. 4a, leaving essentially no space to add functionalities on the foldamer helix to further enhance contacts with the protein surface. In contrast, Po4 was identified as a sizeable (158 Å^3^) cavity nearby P10. Since a P10Q mutation appeared to be feasible without causing steric clashes (Fig. 2c), various side chains were docked in Po4 while being connected to the C5 carbon of the quinoline ring of Q10. All 274 side chains of the Swiss amino acid database^25^ were tested. In each case, the amino acid was replaced by the quinoline residue and Autodock Vina^26^ was used to score interactions between the side chain in position 5 and Po4 (Fig. S4†). Q^5Ph^, Q^5Pa^, and Q^5In^ were selected as having a sufficiently low estimated Δ*G* and as being at the same time synthetically accessible. As presented in detail below, subsequent computational steps, synthesis and structural analysis eventually delivered the solid state structure of the 3·HCAII complex where Po4 is indeed filled by the phenethyl side chains of Q^5Ph^10.

The second surface analysis was performed on the 3·HCAII complex in order to identify potentially ligandable sites in the vicinity of the foldamer helix where foldamer–protein contacts may be extended through the addition of foldamer side chains (Fig. 4c). This analysis led to the identification of pockets Po6–Po9. Po9 consists of the space left in Po4 that is not occupied by the side chain of Q^5Phe^10, hinting at the possibility that this side chain may be further elaborated (Fig. S5†). However, this option was not explored as many of the suggested side chains were synthetically challenging. We focused instead on Po6–Po8 which all lie on the same side of the foldamer helix, and may potentially be reached with side chains on residues Q6, P8, Q11, and Q13 (Fig. 4a). As explained above for Q10, side chains that both had a reasonable docking score and appeared to be synthetically accessible were kept for subsequent investigations (Fig. S6 and S7†). For Q6 and Q11, we have mentioned above that side chains in position 4 of the quinoline do not establish contacts with the protein surface (Fig. 2b) and that these positions were considered for the introduction of chiral B residues to control helix handedness. Instead, the HCAII surface analysis suggested the side chains in position 6 of the quinoline ring might establish contacts with the protein. This eventually led to the mutation of Q^Asp^6 into guanidinium-containing B^Gpr^6 or B^Gpe^6 in sequences 9*, 10*, 13*, 14*, 18, 19, as well as indane-containing residue B^Ida^6 in sequences 8*, 12*, 20. Similarly, mutation of Q^Ace^11 to guanidinum-containing residues Q^6gp^11, Q^6ge^11 or B^Gpr^11 was implemented in sequences 7*–14* and 16–19.

Possible modifications of Q^Asp^13 were inspired by a salt bridge between this residue and Lys24 of HCAII observed in one of the solid state structures presented below. To better reach this Lys24, residues Q^But^13, Q^Bph^13 and Q^5Bu^13 were considered in sequences 7*–9* and 11*–13*. It should be pointed that, given the extensive HCAII surface that the foldamer helix covers, opportunities for mutations and for the creation of new foldamer–protein contacts were too numerous to be considered at the same time. For instance, pockets Po10–Po14 were not investigated (Fig. 4c).

#### Molecular dynamics (MD) simulations

Prior to investing time and resources in the preparation of new momoners and new sequences suggested by the HCAII surface analysis, the effect of foldamer modifications in sequences 3 and 4*–14* on interactions with HCAII were evaluated using MD simulations in explicit water using the AMBER22 package.^27,28^ The initial HCAII and foldamer structures and positions were based on structure alignments to the 3·HCAII solid state structure. The ff14SB force field^29^ was used for α-amino acid residues. The general AMBER force field (GAFF),^30^ with improved torsional parameters for arylamides;^31^ was used for the foldamer (see ESI† for details). One additional simulation in the presence of 125 mM NaCl was performed on 3·HCAII. It resulted in minor changes such as slightly larger fluctuations, deviations, and a reduction of the occurrence of salt bridges. To inspect the interactions between foldamer and HCAII, we carried out a combination of structure visualization, calculations of root mean square displacements (RMSD) of protein and foldamer backbone atoms with respect to the solid state structure (Fig. S8†), as well as analysis of specific residue-to-residue distances. With the exceptions of sequences 7* and 11*,‡‡Sequences 7 and 11 are very similar – they differ only from one methylene group in the side chain of Q11 – so the fact that in both cases the MD trajectories significantly deviate from initial solid state coordinates is probably not a coincidence (Fig. S8). The exact reason is not clear but it appears that the combination of an anionic side chain on Q6 and a cationic side chain on Q11 may lead to this behavior. These two side chains are five units apart, *i.e.* exactly two helix turns, and thus only about 7 Å from each other. Salt bridges between them are observed especially in the case of 7 (40% of the simulation time) which has a longer cationic side chain, and most significant deviation from the solid state structure (Fig. S8). all backbone RMSDs stayed within 3 Å of the solid state structure. Furthermore, sequence pairs with similar sets of mutations (7* and 11*, 8* and 12*, 9* and 13*, 10* and 14*) exhibit similar RMSDs that differ from those of other pairs, with these differences beginning to emerge after approximately 100 ns. Partial dissociation of sequence 7* was observed after 200 ns (Fig. S8†). The observed RMSDs, along with subsequent residue-to-residue distance analyses, support that conformational sampling from the 500 ns trajectory is sufficient for examining the interactions between HCAII and foldamers in the vicinity of the ligand binding site.

**Fig. 5:**
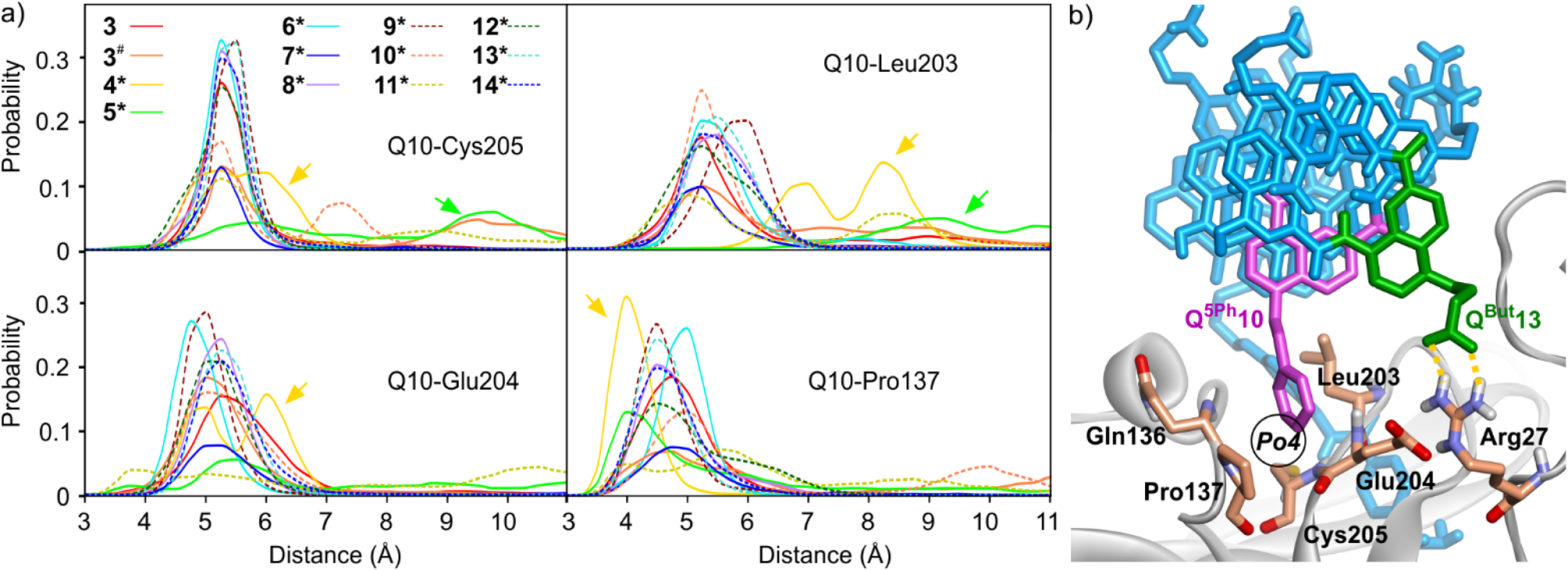
(a) MD simulations of complexes between HCAII and foldamers 3 and 4*–14* showing the occupancy of pocket Po4 by the side chain of Q10, that is, Q^5Ph^10 for all sequences but 4* (Q^5In^10) and 5* (Q^5Pa^10). The histograms show the probability, through the entire simulation time, of the distance between the center of mass (COM) of the phenyl ring of Q^5Ph^10 or Q^5Pa^ or of the pyrrole ring of Q^5In^, and HCAII residues Cys205 (position of Cβ), Glu204 (COM of Cβ and Cγ), Leu203 (COM of all side chain C atoms), and Pro137 (COM of Cβ, Cγ, and Cδ). Two distinct simulations were performed with sequence 3. The simulation marked with # included 125 mM NaCl. Arrows highlight the different positioning of Q^5In^10 and Q^5Pa^10 in the pocket. The histograms show weaker probabilities for sequences 7* and 11* due to strong deviations from the initial structure in these two cases (the Q10 residues are most of the time at distances >12 Å from Po4). (b) Snapshot from the MD simulation of 7*·HCAII showing the relevant residues. In this snapshot, one can also spot a transient salt bridge between the carboxylate side chain of Q^But^13 and the Arg27 (dashed yellow lines). This salt bridge does not occur frequently.

A first aspect concerns the rationale that led to selecting sequences 3 and 4*–14*. Because the number of possible single mutations was large, these were not investigated individually. Most sequences, carry two, three or four simultaneous side chain modifications with respect to 1, at positions 6, 10, 11 and 13. This way, all but two side chain modifications were examined in at least two distinct MD simulations. The first essential result is that the various side chain and, sometimes, main chain modifications mainly depend on where they are implemented, and generally do not depend from one another. When a mutation is performed at a given position, the behavior of the new residue tends not to vary with mutations at other positions. This is a major advantage for making predictions and sharply contrasts with aliphatic peptides where local modifications may impact global behavior.^17^ The consistent behavior of each new residue regardless of other sequence modifications also suggests that no mutation led to a major steric clash that would disturb the whole structure. In addition, owing to the independent behavior of the side chains, we could perform an analysis per interaction site/pocket, instead of an analysis per sequence. The results are presented in Fig. S9–S14† and a representative example is shown in Fig. 5.

The MD simulations reflected the strong interaction between the ligand and HCAII (Fig. S9–S10†). In all simulations, the bond between the HCAII-bound Zn^2+^ ion and the ligand sulfonamide group, tight contacts between the two aryl groups of the ligand and pockets Po1 and Po2, and contacts between Q3 and P5 with HCAII in Po2, all remained well in place (Fig. 2a, 4a and b). Concerning the mutations of P10 implemented to fill Po4, Q^5Ph^10 was found to form stable hydrophobic contacts with Pro137, Leu203, Glu204 and Cys205, as highlighted by the histograms of distance shown in Fig. 5. Note that sequences 4* and 5* show some deviations in these histograms because their different Q^5In^10 and Q^5Pa^10 residues establish distinct contacts. The indole side chain of Q^5In^10 in sequence 4* lies closer to Pro137 (Fig. S12†). This residue was synthesized but sequences containing it had stability issues that hampered experimental investigations. The benzamidinium side chain of Q^5Pa^10 in sequence 5* appeared to be too large for pocket Po4 and its position fluctuated more (Fig. S12†). It was not considered further and Q^5Ph^10 was conserved in all subsequent experiments.

The interactions between side chains on residues 6, 11 and 13 and pockets Po6–Po8 (Fig. S13†) can be summarized as follows. Overall, the distance histograms show larger variations than for the contacts in pockets Po1–Po4. The salt bridge between Q^Asp^13 and Lys24 seen in one solid state structure (see below) was absent or present in small percentage (3% to 41%) of the time along the trajectories in aqueous solution. Using other negatively charged side residues Q^But^13, Q^Bph^13, and Q^5Bu^13 made little difference. These were therefore not tested experimentally. In the case of residue 6, the hydrophobic side chain of B^Ida^6 or the cationic side chains of B^Gpe^6 or B^Gpr^6 could potentially form contacts with Phe20 and Asp19, respectively, within pocket 6. Some of these residues were subsequently synthesized and implemented in sequences 19–20. Finally, the benefit of cationic residues in position 11 to fill pocket Po8 was not clear. Salt bridges were established only during small fractions of simulation time. With sequence 10*, a possible exception to the independent role of the side chains was observed with an apparent positive cooperative effect of the guanidinium-containing side chains of B^Gpe^6 and Q^6gp^11 (Fig. S13†). Specifically, salt bridging (59% of simulation time for Q^6gp^11–Asp19 and 95% for B^Gpe^6–Asp19) and hydrogen bonding (68% of simulation time for Q^6gp^11–Asp19) are more frequent in 10*·HCAII than with 7*, 8*, 9* and 14* in which either B^Gpe^6 or Q^6gp^11 is present (0 to 28% for Q^6gp^11–Asp19, 74% for B^Gpe^6–Asp19 and 0–11% H-bond for Q^6gp^11–Asp19).

MD simulations revealed an additional, unplanned, favorable foldamer–protein contact between Q^Hyd^7 and Gln135 (Fig. S14†). As mentioned above, P7Q^Hyd^ and P12Q^Hyd^ mutations were introduced to rigidify the helix and mitigate the risk that chiral B residue may not quantitatively bias helix handedness. Q^Hyd^ residues were selected for that purpose because their relatively acidic yet small hydroxy side chain would not decrease foldamer water solubility. Nevertheless, MD simulations suggest that the side chain of Q^Hyd^7 can also hydrogen bond to Gln135.

### Structure elucidation

Crystallization was attempted for all foldamer–HCAII complexes. In the case of 2·HCAII, 3·HCAII, 16·HCAII and 20·HCAII, single crystals suitable for X-ray diffraction analysis were obtained (Fig. S15†) and the solid state structures were elucidated in the *P*2_1_2_1_2 space group at a resolution of 1.4, 2.1, 1.6, and 2.1 Å, respectively (Fig. S16 and S17†). For the four structures, crystallization conditions were similar to that of 2·HCAII, and so were the unit cells and packing arrangements. Some parts of the foldamer molecule are involved in intercomplex contacts in the crystal lattice, including the side chain of Q^Ace^11 (Fig. 4a and S18†), these contacts are all conserved in foldamers 1, 2, 3, 16 and 20. In retrospect, we hypothesized that the unsuccessful crystallization of the complexes with 17, 18 and 19 may be assigned to the mutation of Q^Ace^11 into B^Gpr^11 or Q^6gp^11 in these compounds.

**Fig. 6:**
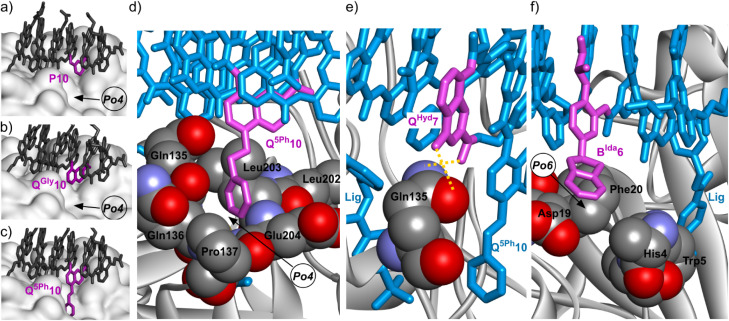
Solid state structures of HCAII in complex with: (a) 1, (b) 2, (c) 3, (d and e) 16, (f) 20. In (a)–(c), the protein is shown as a white soft surface and the foldamer is shown in stick representation in gray except the residue in position 10 colored in purple. In (d)–(f), the protein is shown in gray ribbon representation except relevant amino acids which are in space filling representation. The foldamer is shown in blue stick representation except the residue in position 10 in (d), the residue in position 7 in (e) and the residue in position 6 in (f), which are colored in purple. Hydrogen bonds are indicated as yellow dashed lines. Pockets Po4 and Po6 are defined in Fig. 4.

**Fig. 7:**
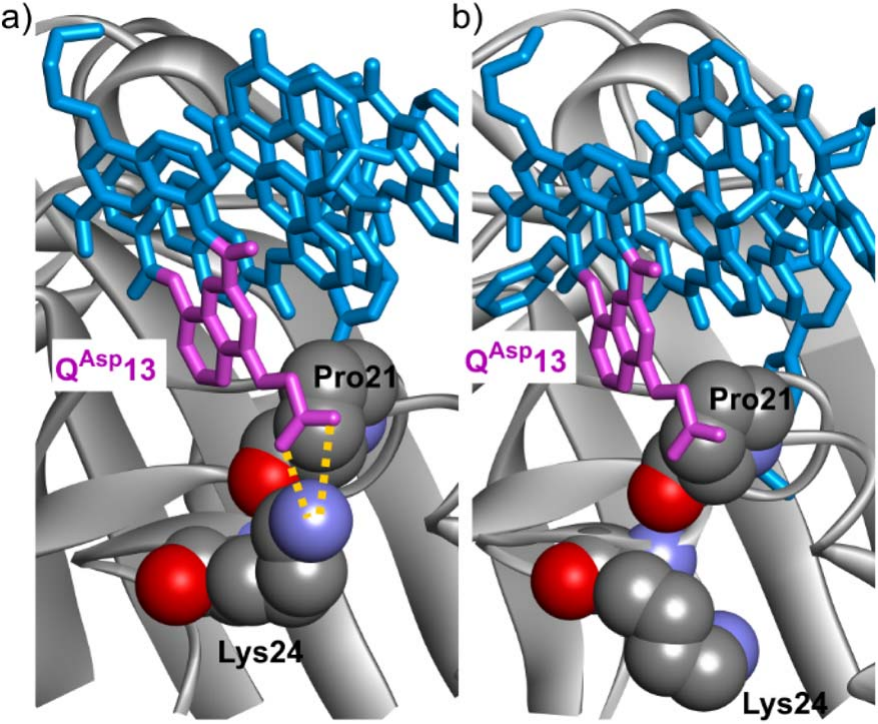
Solid state structures of HCAII in complex with: (a) 2, (b) 20. The protein is shown in gray ribbon representation except P21 and Lys24, which are in space filling representation. The foldamer is shown in blue stick representation except the residue in position 13, which is colored in purple. Hydrogen bonds (here within a salt bridge) are indicated as yellow dashed lines.

The structure of 2·HCAII validated that a P10Q^Gly^ mutation of the foldamer could be performed without any steric apparent clash between the protein and Q^Gly^10 or any alteration of the foldamer helix shape (Fig. 6a and b). This structure also revealed a salt bridge between the carboxylate of the Q^Asp^13 and residue Lys24 (Fig. 7a). In the structure of 1·HCAII, the side chain of Lys24 was only partly visible in the electron density map and the salt bridge was overlooked. As mentioned above, MD simulations suggested that this salt bridge is not stable in aqueous solution and is not convincingly stabilized when using anionic side chains longer than in Q^Asp^13, or placed in position 5 of the quinoline ring, or having a dianionic phosphonate group, as in Q^But^, Q^Bph^, and Q^5Bu^.

The structure of 3·HCAII then validated that the phenethyl side chain of Q^5Ph^10 filled Po4 as predicted by computations (Fig. 6c and d). The methylene carbon atom linked to the quinoline ring was found at 3.9 Å from a methyl group of Leu203, and one carbon atom of the phenyl ring lies within 3.5 Å from the nitrogen atom of Pro137. The structure of 16·HCAII confirmed the position of Q^5Ph^10 found in 3·HCAII and validated the double mutation Q^Asp^6B^Gly^ and P7Q^Hyd^ intended to introduce helix handedness bias (through B^Gly^6) and to make the helix more rigid (through Q^Hyd^7). The proximity of Q^Hyd^7 and Gln135 (Fig. 6e) makes the hydrogen bonding observed in MD simulations plausible. In the solid state, the Gln135 amide NH_2_ hydrogen bonds to the main chain carbonyl of Q^Hyd^7. In addition, the proximity between the primary amide of Gln135 and the hydroxy side chain of Q^Hyd^7 likely favors contacts with the latter as well, be it in a protonated or deprotonated state. Finally, the structure of 20·HCAII validated that the indane side chain of B^Ida^6 filled Po6 again as predicted by computations, establishing contacts with Phe20 (Fig. 6f). In this structure, Lys24 was again visible in the electron density map, but in a conformation where hydrogen bonding to Q^Asp^13 is not established (Fig. 7b), different from the structure of 2·HCAII. Of note, in all structures, Q^Asp^13 is involved in a salt bridge with a lysine (Lys80) belonging to another HCAII molecule of the crystal lattice as part of the intercomplex contacts (Fig. S18†). This probably influences, that is, competes with the formation of the salt bridge with Lys24 in the solid state.

Altogether, the solid state structures validate the predictions made by computations. They demonstrate the equivalent contribution to main chain helix curvature of P, Q and B monomers in the context of a foldamer–protein contact area. They also demonstrate that the positions of the foldamer side chains and the type of interactions they may engage with the protein are predictable.

### Binding studies

**Fig. 8:**
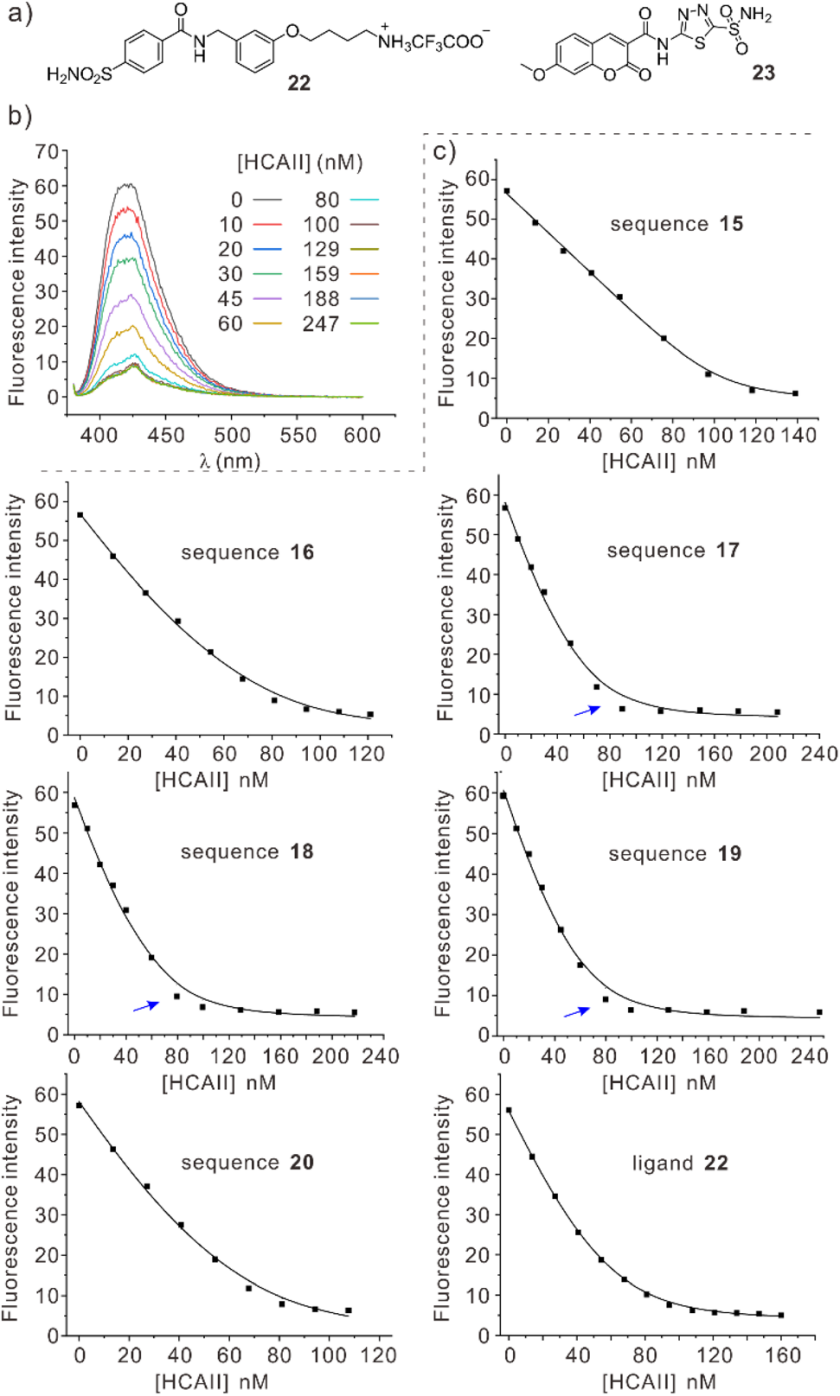
(a) Structural formula of HCAII ligand 22 and of fluorescence probe 23 used in the competition assay. (b) Changes in fluorescence spectra (380–600 nm) upon HCAII titration of sequence 19. [19] = [23] = 50 nM; (c) Experimental (■) and calculated values using a 1 : 1 binding isotherm (−) of fluorescence intensity of sequences 15–20 and 22 titrated with HCAII. [competing species] = [23] = 50 nM. Note that the curve fitting is shown at one wavelength (419 nm) as an illustration but that the *K*_D_ values (Table 1) were calculated by simultaneously fitting data recorded in the 380–600 nm range. Blue arrows point to a systematic error discussed in the text.

We set out to measure the binding affinities of *P*-helical, chiral B-containing sequences 15–20 for HCAII to assess the extent to which they reflected the changes introduced in the foldamers. Sequences 1–3 are potentially problematic as they exist as a racemic mixture of *M*- and *P*-helical conformers that must have different *K*_D_ values and whose proportion evolve with time upon binding to HCAII, hence the focus on 15–20. This assessment proved challenging. Simple ligands such as 22 (Fig. 8a) – the fragment of 15–20 that fills pockets Po1 and Po2 of HCAII – bind in the low nM range. Getting accurate *K*_D_ values to comment on potentially small effects for such strong binding is delicate. Furthermore, we have shown that appending a foldamer on 22 has one major consequence: both the association and dissociation kinetics are slowed down by almost two orders of magnitude.^8*a*^ Unlike with classical small molecule HCAII ligands, dissociation becomes so slow that techniques such as surface plasmon resonance (SPR) or biolayer interferometry (BLI) no longer deliver reliable results.

We turned to a recently published assay that exploits the quenching of the fluorescence of nanomolar ligand 23 upon binding to HCAII (Fig. 8a).^32^ The low *K*_D_ value of 23 makes it suitable to perform competition (displacement) assays with compounds binding in the same concentration range. However, some optimization of the assay was required to perform experiments with foldamer-containing ligands because the foldamers absorb both at the excitation (373 nm) and emission (400–450 nm) wavelengths of 23. Performing classical direct displacement titrations where a foldamer is added to a solution containing 23 and HCAII would be complicated by variable inner filter effects. Instead, we performed titrations in which aliquots of an HCAII solution, typically 10 μM, were added to a solution already containing a foldamer (50 nM) and 23 (50 nM). The foldamer and 23 were also present at the same concentrations in the HCAII solution. This way, the concentrations of fluorophore and foldamer were kept constant and only the ratio of HCAII was varied. A representative titration is shown in Fig. 8b and the corresponding *K*_D_ values are shown in Table 1. With this assay, the *K*_D_ value for simple ligand 22 was 10 nM compared to 5 nM previously measured by SPR with HCAII immobilized on the SPR chip under slightly different buffer conditions.^8*a*^§§For SPR measurements: 2 : 98 DMSO/aqueous phosphate saline buffer PBS at pH 7.4 (vol/vol) at 25 °C, with PBS = 10 mM Na_2_HPO_4_, 1.8 mM KH_2_PO_4_, 2.7 mM KCl and 137 mM NaCl. For fluorescence measurements: 50 mM HEPES (4-(2-hydroxyethyl)-1-piperazineethanesulfonic acid) at pH 7.2. For the titrations of the foldamers, one may note a slight but persistent systematic deviation in the curve fitting of the data by the 1 : 1 binding isotherm (blue arrows in Fig. 8). It appears that the fluorescence drops upon adding HCAII, *i.e.* that ligand 23 binds to HCAII, in presence of the foldamer faster than predicted by the binding model. A better fit can be artificially obtained by inputting HCAII concentrations 20% larger than those measured or foldamer concentrations 20% lower than those measured in the curve fitting program. Using a 2 : 1 binding model (two foldamers per HCAII) also gave a better fit. The results are nevertheless presented as observed because the modifications that lead to a better fit have no experimental justification. Furthermore, these modifications do not change the trend and conclusions presented below.

**Table 1:** Dissociation constants of the complexes formed with HCAII determined by the fluorescence competition assay

HCAII binder^a^	*K*_D_ b (nM)
22 (reference ligand)	10
15	1.5
16 (with Q^5Phe^10)	5.2
17 (with Q^5Phe^10, B^Gpr^11)	10
18 (with B^Gpr^6, Q^5Phe^10, B^Gpr^11)	7.2
19 (with B^Gpr^6, Q^5Phe^10, B^6gp^11)	9.3
20 (with B^Ida^6, Q^5Phe^10)	7.4

a Some remarkable features are indicated in parenthesis.

b Values were found to be repeatable within ±15% in duplicate experiments.

**Fig. 9:**
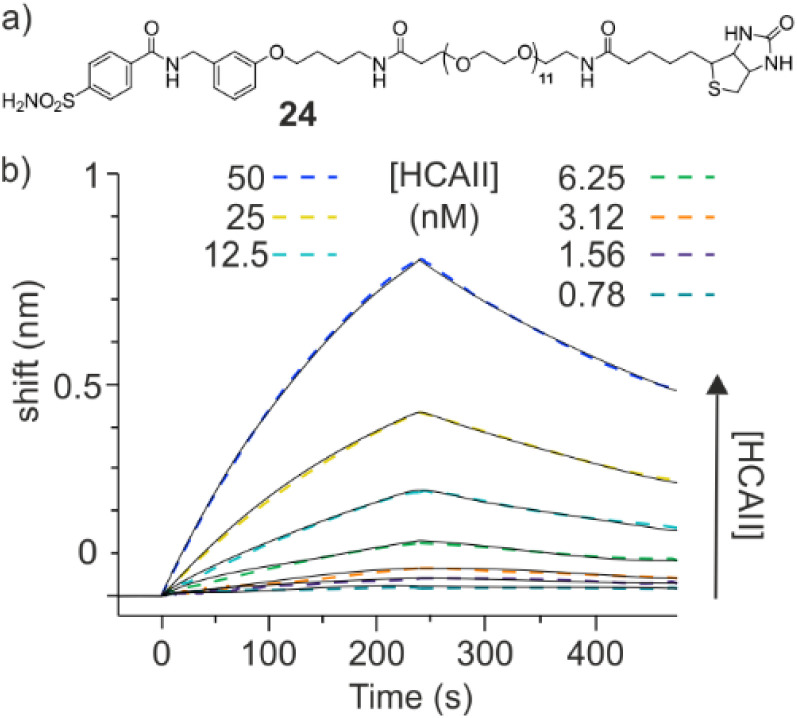
(a) Structural formula of biotinylated HCAII ligand 24. (b) BLI sensorgrams (black solid lines) of the titration of 24 immobilized on the streptavidin sensors at different HCAII concentrations. Calculated curves based on a 1 : 1 binding isotherm (colored dashed lines) fit with the measured values, yielding *K*_D_ = 38.2 nM.

In order to confirm these data, we tried to develop an alternate competition assay using BLI. A new biotinylated HCAII ligand 24 (Fig. 9a) was synthesized which, after immobilization on streptavidin sensors allowed for an accurate *K*_D_ determination of its association with HCAII (Fig. 9). Immobilized 24 may in principle act as a reporter of the concentrations of free HCAII in solution. However, in this case as well, the kinetics were slow, a steady state regime was not reached. A calibration curve could in principle be produced by intercepting a value on the sensorgrams after a fixed amount of time instead of waiting until a steady state is reached. However, this proved not to be accurate enough to reliably determine the free HCAII concentration in solution.

Coming back to the *K*_D_ values measured with the fluorescence competition assay, it appears that sequence 15, with no added side chain in positions 6, 10 and 11, is the best binder and that all others bind similarly. These results should nevertheless be taken with caution. The foldamers suffer from low water solubility and a contribution from foldamer aggregation cannot be excluded. For instance, when the fluorescence titrations were performed at higher concentrations (*e.g.* [23] = 100 nM, [foldamer] = 200 nM, Fig. S19†), the apparent *K*_D_ were higher than in Table 1, consistent with an effect of aggregation that reduces the effective foldamer concentration for binding to HCAII. The values in Table 1 could therefore also reflect that sequences 16–20 aggregate more than 15, *e.g.* because of their hydrophobic Q^5Ph^10 residue.

## Discussion

It appears that none of the additional side chain combination of 16–20 result in a strong enhancement of their affinity for HCAII. It should be pointed that the structure-based design is intended to stabilize the complex, that is, to slow down complex dissociation. The effect of the additional side chains on the kinetics of complex formation, *e.g.* potentially slowing it down, remains unknown and is not taken into account in the computations. Early studies on HCAII ligands had shown that higher affinity correlated with faster complex formation rather than slower complex dissociation.^33^ Another early study also reported the lack of effect of extending an HCAII ligand in the search for secondary binding sites, a result comparable to ours in an approach conceptually similar, albeit with much smaller molecules.^34^ Finally, it may be that the very architecture of foldamers 15–20 makes it difficult for side chain modification to result in strong effects. These compounds consist of small nanomolar ligand to which is appended a much larger foldamer that has inherently no affinity for HCAII even in the low micromolar range – no induced CD is observed at 35 μM with 21 which lacks an HCAII ligand (Fig. S3†). In other words, the starting affinity of the foldamers for HCAII is too low to hope that a few modifications will bring it to an interesting range of *K*_D_ value. In this respect, the choice of HCAII as a model system was perhaps not ideal. HCAII is a therapeutically relevant target and transmembrane isoforms HCAIX and HCAXII are overexpressed in some cancers and identified as potential targets as well.^24,28^ Nevertheless, we selected HCAII as a model system mainly for its robustness, easy overexpression, good crystal growth ability and the availability of simple nanomolar ligands that could act as tethers. To our knowledge, the vicinity of the HCAII active site is not involved in protein–protein interactions and deprived of any hotspot that may facilitate foldamer binding.

## Conclusions

In summary, starting from the crystal structure of the complex between HCAII and tetradecaamide foldamer 1, we have used computational tools to identify main chain and side chain modifications that may result in an extended foldamer–protein interface. New monomers and sequences incorporating these monomers were synthesized and several solid state structures of complexes with HCAII validated the design principles. We find that Q, B, and P main chain variations are interchangeable also in the context of a foldamer–protein complex. We also find that side chains may generally be introduced independently from one another, a result that was consistent in both MD simulations and solid state structures. This behavior is in sharp contrast with that of peptides and peptidic foldamers in which a local change, *e.g.* a side chain modification, may also result in a different behavior of the main chain.^17^ The robustness of aromatic foldamer helices should therefore represent a good starting point for the structure-based design of protein binders that cover large protein surface areas. Nevertheless, the modifications explored in this study did not result into stronger associations nor did they deliver foldamers that would bind HCAII without a ligand or a covalent tether to mediate the interactions. The grand challenge of the *ab initio* design of an aromatic foldamer to bind a given protein surface remains unmet. In the meantime, other studies have revealed the potential of some aromatic helical foldamers to mimic α-helices^13*a*^ or DNA double helices.^10^ Solid state structures have been obtained of complexes between chromosomal protein Sac7d and a DNA-mimic foldamer^10*d*^ and between a fragment of ubiquitin ligase E6AP and a foldamer–peptide macrocycle.^35^ In these complexes, no ligand or covalent tethering are involved. Furthermore, reliable *K*_D_ determination methods are available. These structure thus represent new candidates to apply the structure-based design principles validated here. Steps in these directions are being made and will be reported in due course.

## Author contributions

Except for the first and last authors, the author list groups the authors by institution and does not reflect a ranking of their contributions. LW, PSR and CD performed solution phase and solid phase syntheses. LW performed protein expression. BLE, LW and JS performed crystal growth. LF, JS and TG carried out crystallographic structure elucidation. LW and CD performed fluorescence and BLI titrations. ZL and YY performed computational studies. VP, YZ and IH supervised the research. IH, LW, CD and ZL wrote the manuscript. All authors reviewed and edited the manuscript and approved its final version.

## Conflicts of interest

There are no conflicts to declare.

## Supplementary Material

SC-016-D5SC01826A-s001

## Data Availability

The data supporting this article have been included as part of the ESI.† Crystallographic data for 2·HCAII, 3·HCAII, 16·HCAII and 20·HCAII has been deposited at the Protein Data bank under accession numbers 9GAM, 9GAK, 9GAJ, 9HGB, respectively, and can be obtained from https://doi.org/10.2210/pdbXXXX/pdb where XXXX is the accession number.
