# Supplementary material for: Structure-based design of an aromatic helical foldamer–protein interface
Source: Chem Sci. 2025 Jun 2;16(27):12385–96. doi: 10.1039/d5sc01826a (PMC12154549; doi:10.1039/d5sc01826a)
Supplement: SC-016-D5SC01826A-s001 [file SC-016-D5SC01826A-s001.pdf]

Supplementary Information (SI) for Chemical Science.
This journal is © The Royal Society of Chemistry 2025

Supplementary Information

Structure-based design of an aromatic helical foldamer-protein interface

Lingfei Wang,[a] Céline Douat,[a] Johannes Sigl,[a] Post Sai Reddy,[b] Lucile Fischer,[b] Béatrice Langlois d'Estaintot,[b] Zhiwei Liu,[c] Vojislava Pophristic,[c] Yuwei Yang,[d] Yingkai Zhang[d] and Ivan Huc*[a]

[a.] *Department Pharmazie, Ludwig-Maximilians-Universität, Butenandtstr. 5–13, 81377 München (Germany). E-mail: ivan.huc@cup.lmu.de.*
[b.] *CBMN (UMR5248), Univ. Bordeaux-CNRS-INP, Institut Européen de Chimie et Biologie, 2 rue Escarpit, 33600 Pessac (France).*
[c.] *Department of Chemistry & Biochemistry, Rowan University, 201 Mullica Hill Road, 08028 Glassboro, New Jersey (USA).*
[d.] *Department of Chemistry, New York University, 10003 New York, New York (USA)*

Table of contents

1. Supplementary Figures and Tables ... 3
  1.1 Supplementary figures ... 3
  1.2 Supplementary tables ... 23
2. Computational studies ... 30
  2.1 Pocket analysis and pocket-centric side chain screening ... 30
  2.2 Molecular dynamics simulations ... 30
3. Crystallography ... 31
4. Biophysical measurements ... 33
  4.1 CD and UV-Vis spectroscopy ... 33
  4.2 Fluorescence binding assay ... 34
    4.2.1 K_D measurement of the 23•HCAII complex ... 34
    4.2.2 Sensor aggregation and reasons for protocol optimization ... 35
    4.2.3 K_D measurement of HCAII complexes with AOFs 15-20 and ligand 22 ... 37
  4.3 BioLayer Interferometry ... 38
5. Chemical Synthesis ... 39
  5.1 General ... 39
  5.2 Experimental procedures for chemical synthesis and purification ... 40

5.2.1 Solid phase synthesis and purification of aromatic oligoamide foldamers (AOFs) 40
5.2.2 Attachment of the Ligand and the DEG tail on resin-bound AOFs 40
5.3 AOF monomer and biotinylated HCAII ligand synthesis 42
5.4 Foldamer synthesis on solid support 51
5.5 NMR spectra and RP-HPLC chromatograms of new compounds 57
6. References 86

1. Supplementary Figures and Tables

1.1 Supplementary figures

Biotinylated HCA II Ligand Synthesis

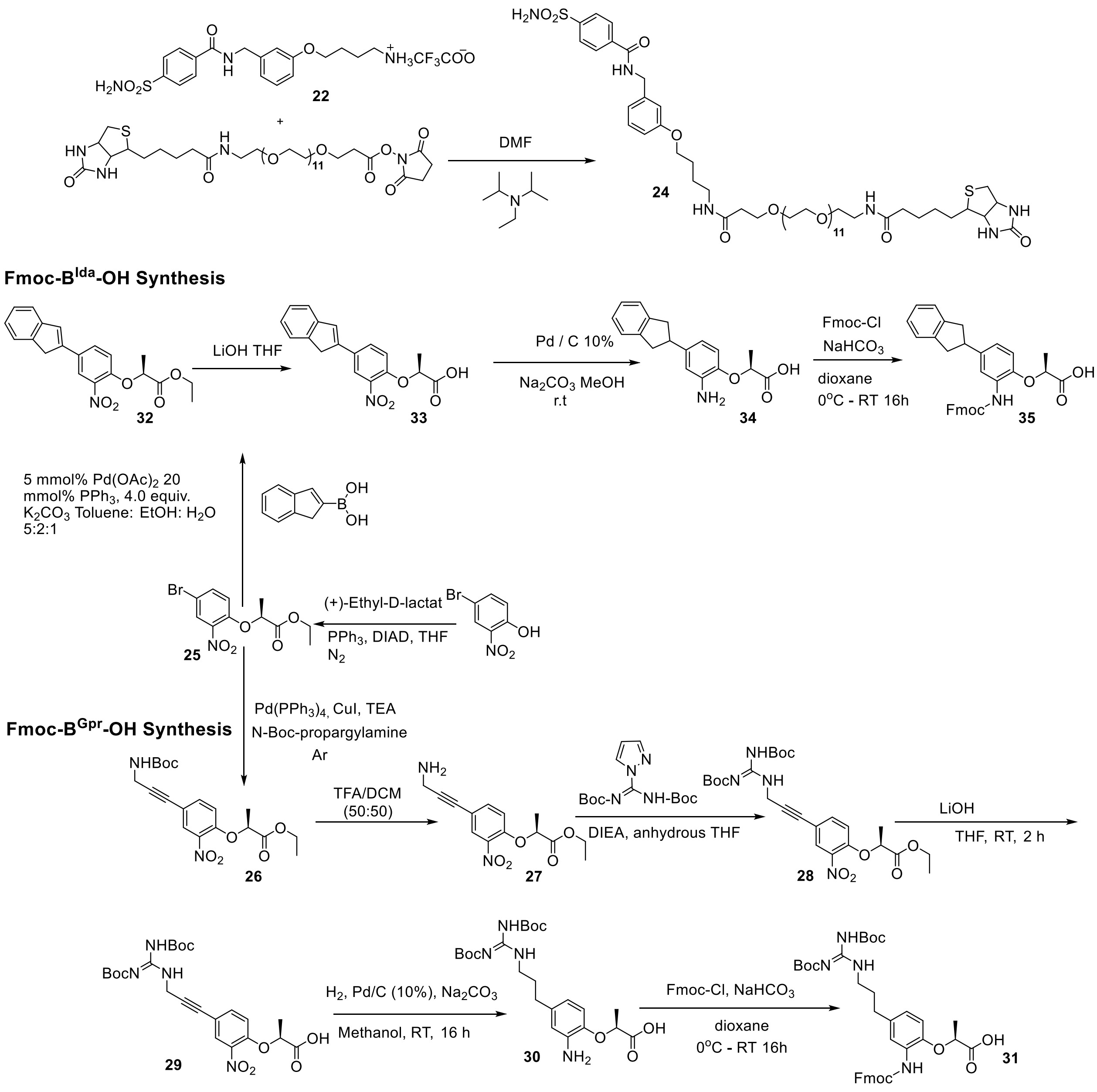

Figure S1 (part 1). Synthetic route of new monomers **31**, **35**, **39**, **43**, **45**, and **48** and of the biotinylated HCAII ligand.

Fmoc-Q^{5Ph}-OH Synthesis

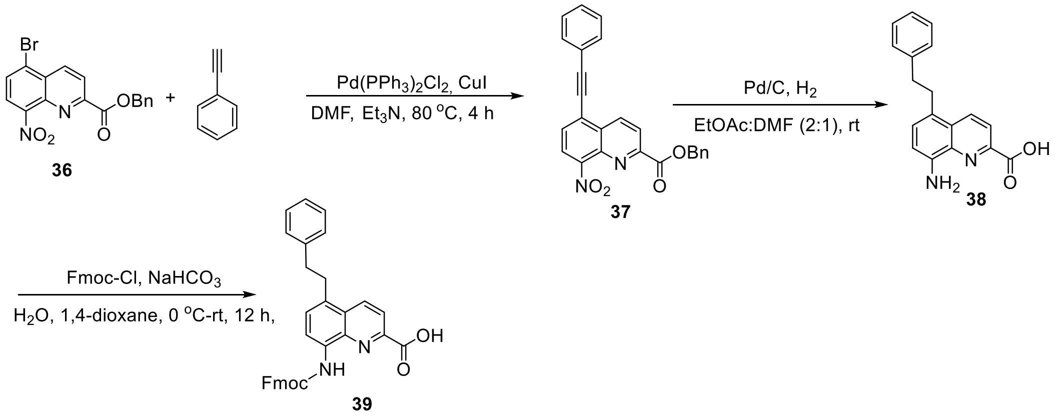

Fmoc-Q^{5In}-OH Synthesis

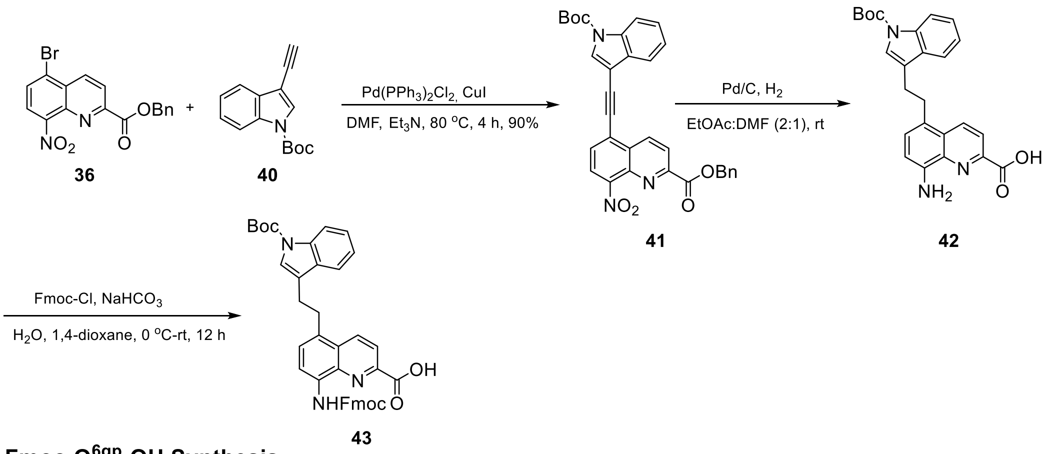

Fmoc-Q^{6gp}-OH Synthesis

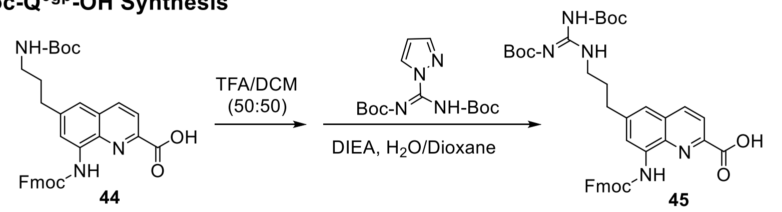

Fmoc-Q^{Gly}-OH Synthesis

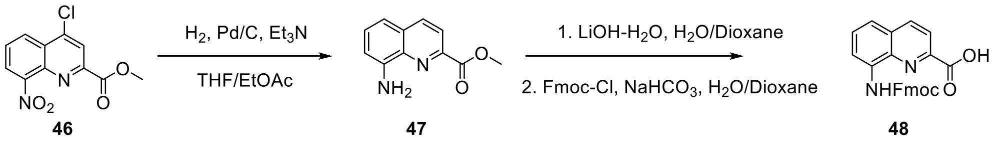

Figure S1 (part 2). Synthetic route of new monomers **31**, **35**, **39**, **43**, **45**, and **48** and of the biotinylated HCAII ligand.

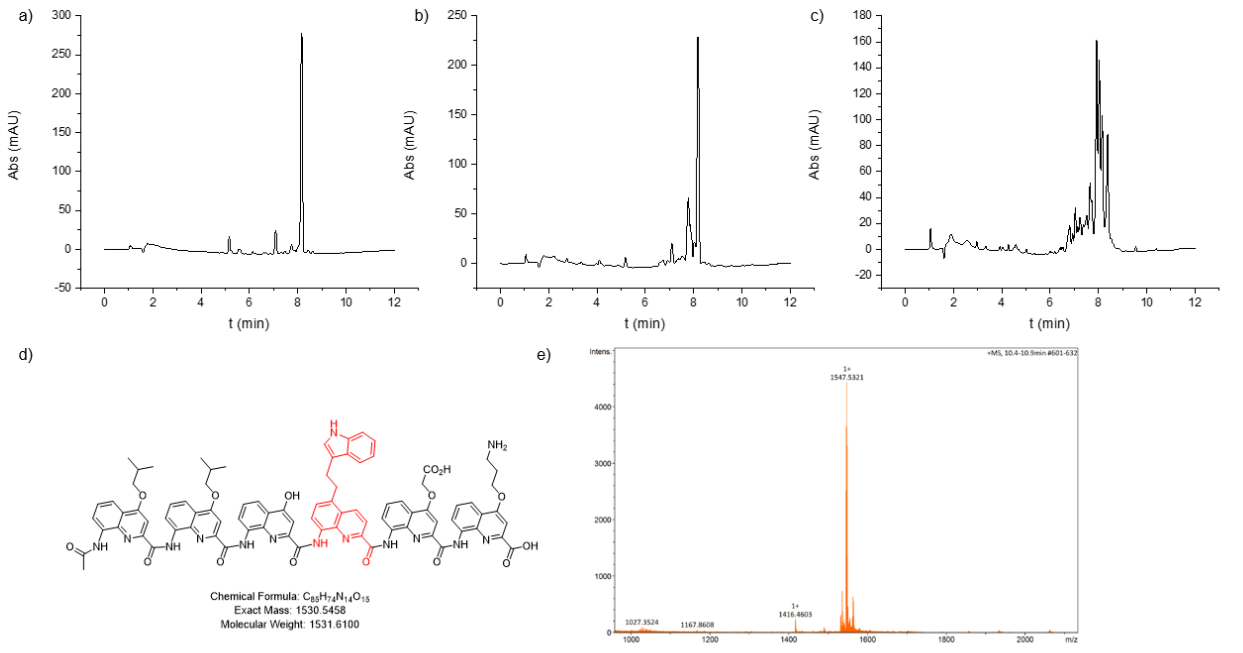

Figure S2: HPLC monitoring of the degradation of a model sequence containing Q^{5In} in water/acetonitrile (1:1 v/v).RP-HPLC profiles of the solution at day 0 (a), day 3 (b) and day 6 (c) (gradient 10% to 100% ACN, 10min, 50 °C, λ = 254 nm). The chemical structure is shown in (d). The mass spectrum in e) shows a main peak corresponding to the initial sequence and a species at M+16, which corresponds to a first oxidation step.

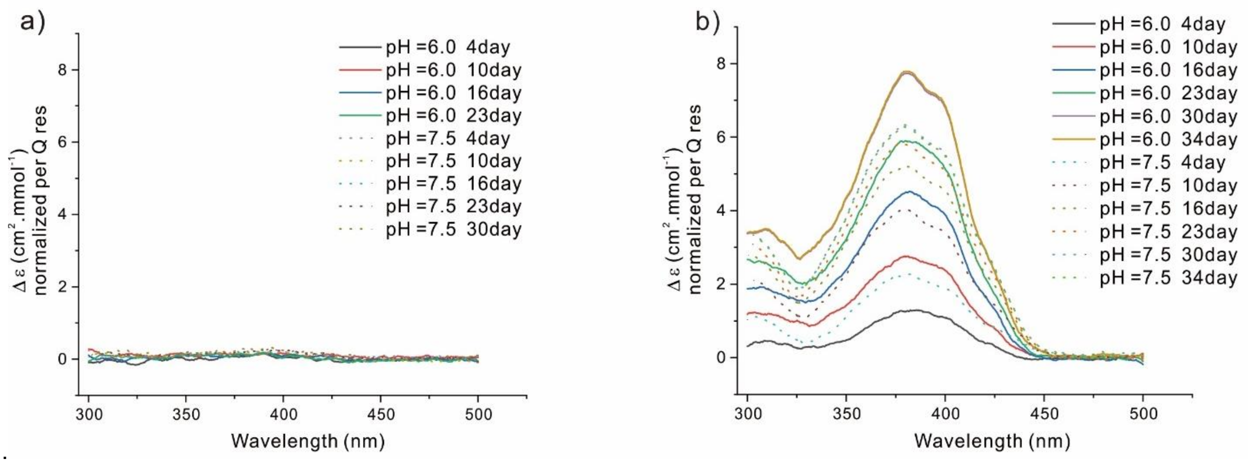

.

Figure S3. CD spectra of **21** (a) and **3** (b) in the presence of HCAII in phosphonate buffer at pH 6.0 and 7.5 at 20 °C. The concentrations of HCAII and foldamers are both 34.5 μM. Procedures are described in chapter 4.1. HCAII is a particularly robust protein that has been used as a model system in many studies for that reason. It can be purchased as a white, freeze-dried, powder and remains suitable for crystal-growth upon long term storage. In these experiments, the actual stability of HCAII was not experimentally verified. However, since an increase of CD intensity was observed, we inferred that the ligand remained bound and therefore the protein kept its structure. Furthermore, UV-spectra were measured alongside CD spectra. The absence of drop in UV-vis absorbance indicated that no precipitation took place that could have remained invisible to the naked eye.

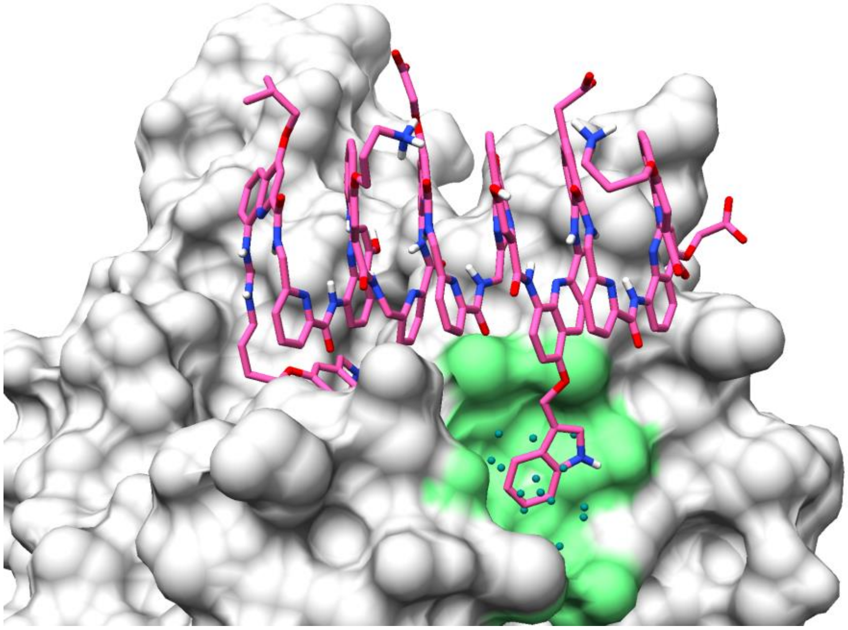

Side chain	ΔG (kcal/mol)
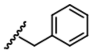 Q^{5Ph}	-1.4
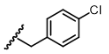	-1.7
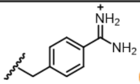 Q^{5Pa}	-2.2
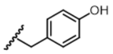	-1.6
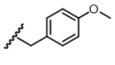	-1.7
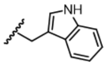 Q^{5In}	-2.0
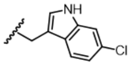	-2.1
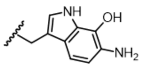	-2.5

Figure S4. Docking and scoring of side chains in position 5 of residue Q10 for their interaction with Po4. Note that side chains in position 4 or 6 may be too far from, or too close to, pocket 4. Hence, proposed side chains were placed in position 5. The top scorers are shown.

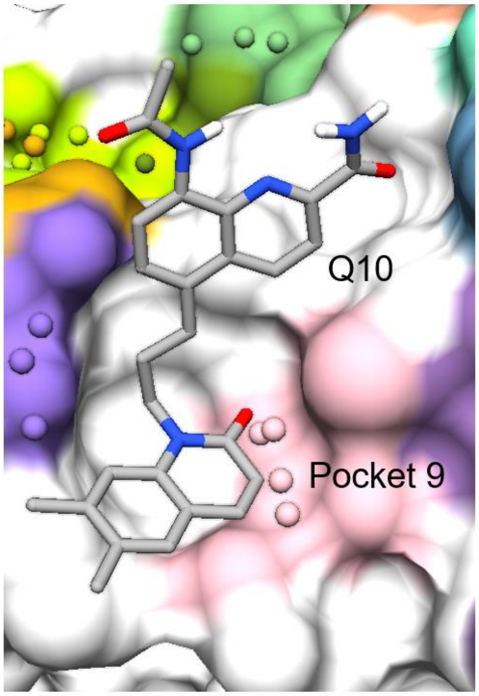

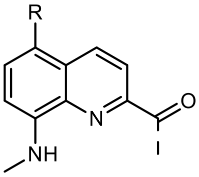

Q10

Side chain	ΔG (kcal/mol)
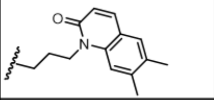	-2.6
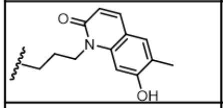	-2.5
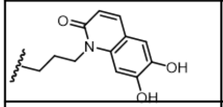	-2.5
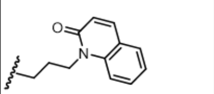	-2.4
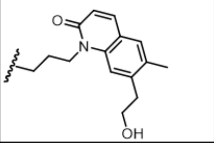	-2.3

Figure S5. Docking and scoring of side chains in position 5 of residue Q10 for their interaction with Po4/Po9. The side chains were not derived from the Swiss side chain database but were manually generated. These side chains were not further investigated.

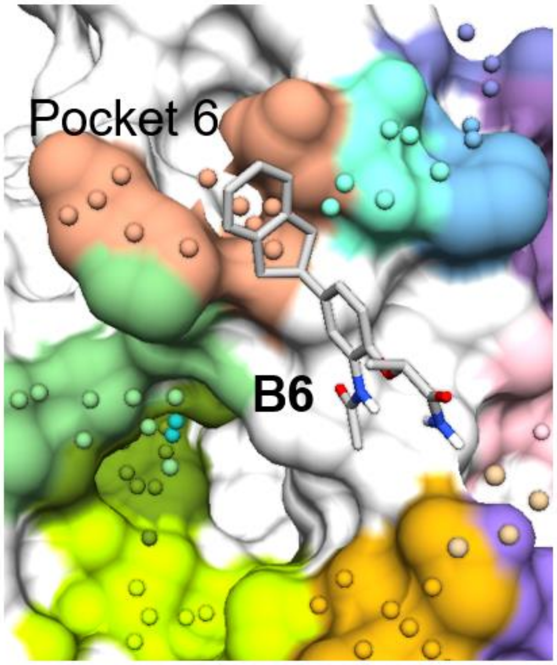

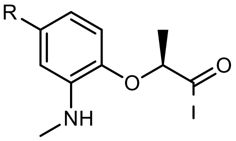

B6

Side chain	ΔG (kcal/mol)
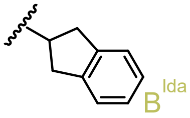	-1.11
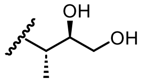	-0.93
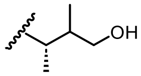	-0.77
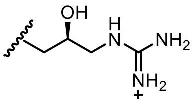	-0.77
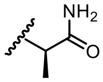	-0.71

B^{Gpr} ← (without OH group)

Figure S6. Docking and scoring of side chains of residue B6 for their interaction with Po6. The indane side chain was implemented in monomer B^{Ida}. The guanidinium-containing side chain without its hydroxy group was implemented in monomer B^{Gpr}.

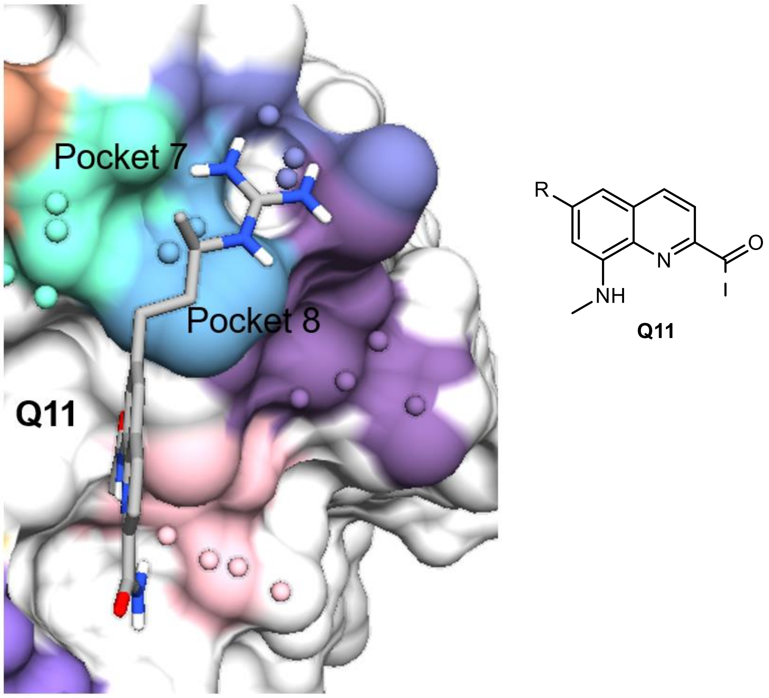

Side chain	ΔG (kcal/mol)
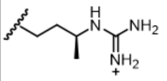	-1.15
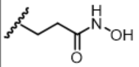	-0.99
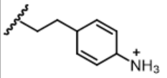	-0.96
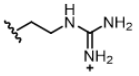 Q^{6ge}	-0.91
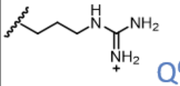 Q^{6gp}	-0.90

Figure S7. Docking and scoring of side chains ion position 6 of residue Q11 for their interaction with Po7 and Po8. Both guanidinium-containing side chains were implemented in this study in monomers Q^{6ge} and Q^{6gp}.

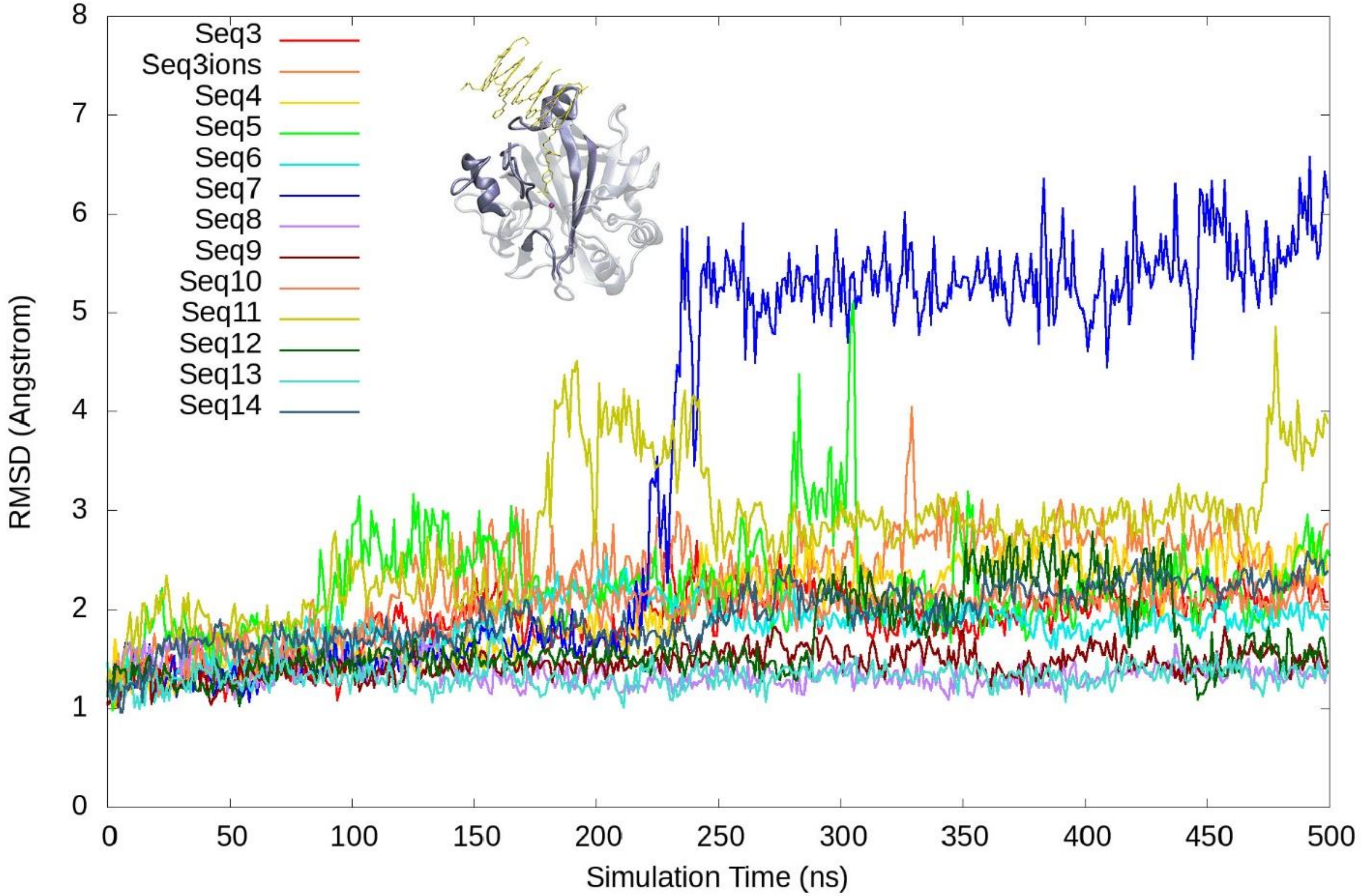

Figure S8. RMSD of protein and foldamer backbone atoms with respect to **3**•HCAII solid state structure along the 500 ns trajectories. Most sequences except **5*, 7*** and **11*** stay within 3 Å to the solid state structure with different levels of deviation or fluctuation. The inserted figure is a snapshot of **7***•HCAII in the second half of the simulation, deviating significantly from the initial structure and showing partial dissociation of the foldamer from the protein surface

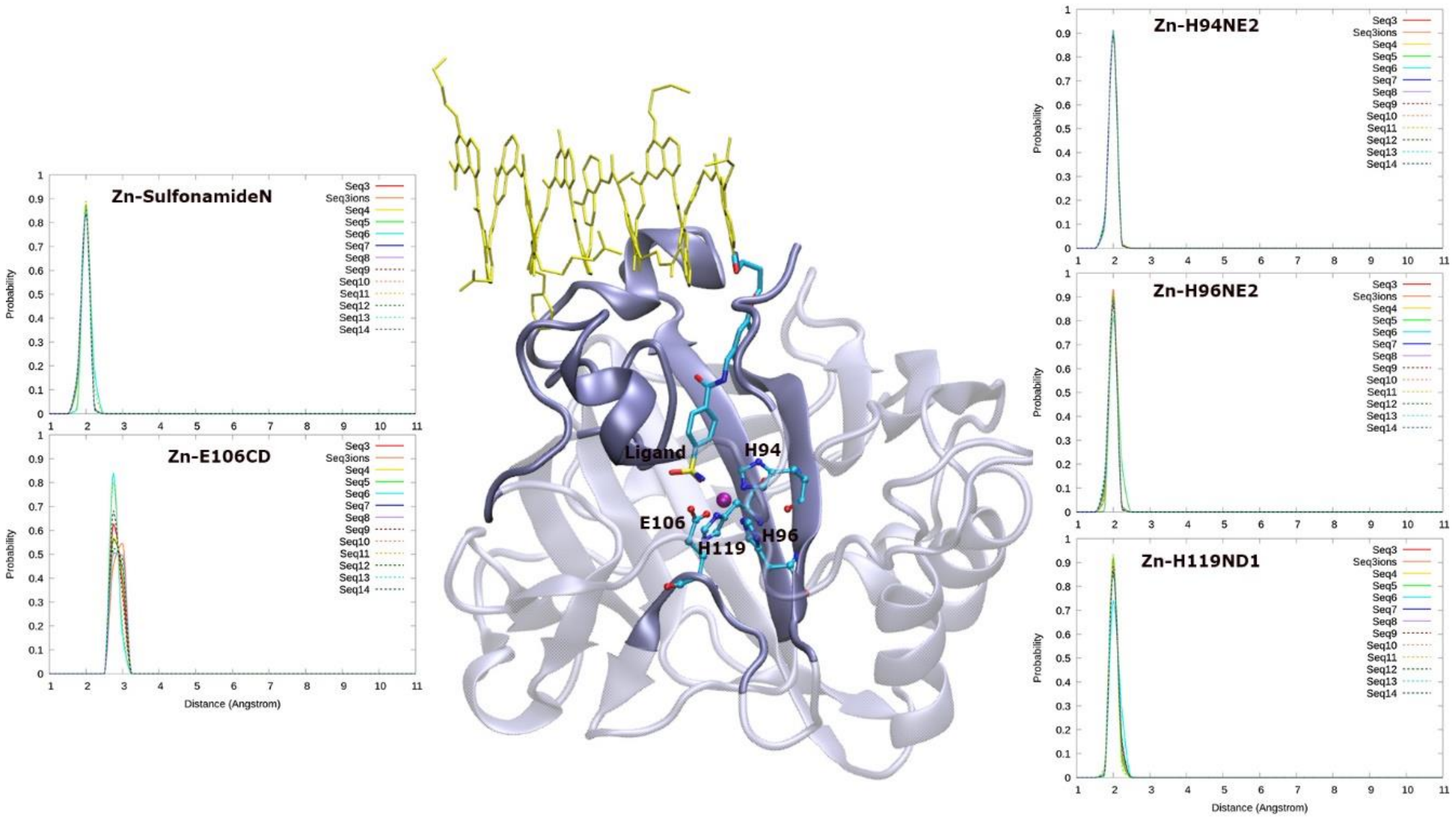

Figure S9. Binding at the ligand binding site. Histograms of distances between Zn^{2+} to HIS94 tele-N, HIS96 tele-N, HIS119 pros-N (right), ligand sulfonamide N and GLU106 δC are calculated based on 500 ns trajectory. The first four distances are constrained during simulation using a weak force constant to ensure proper ligand binding.

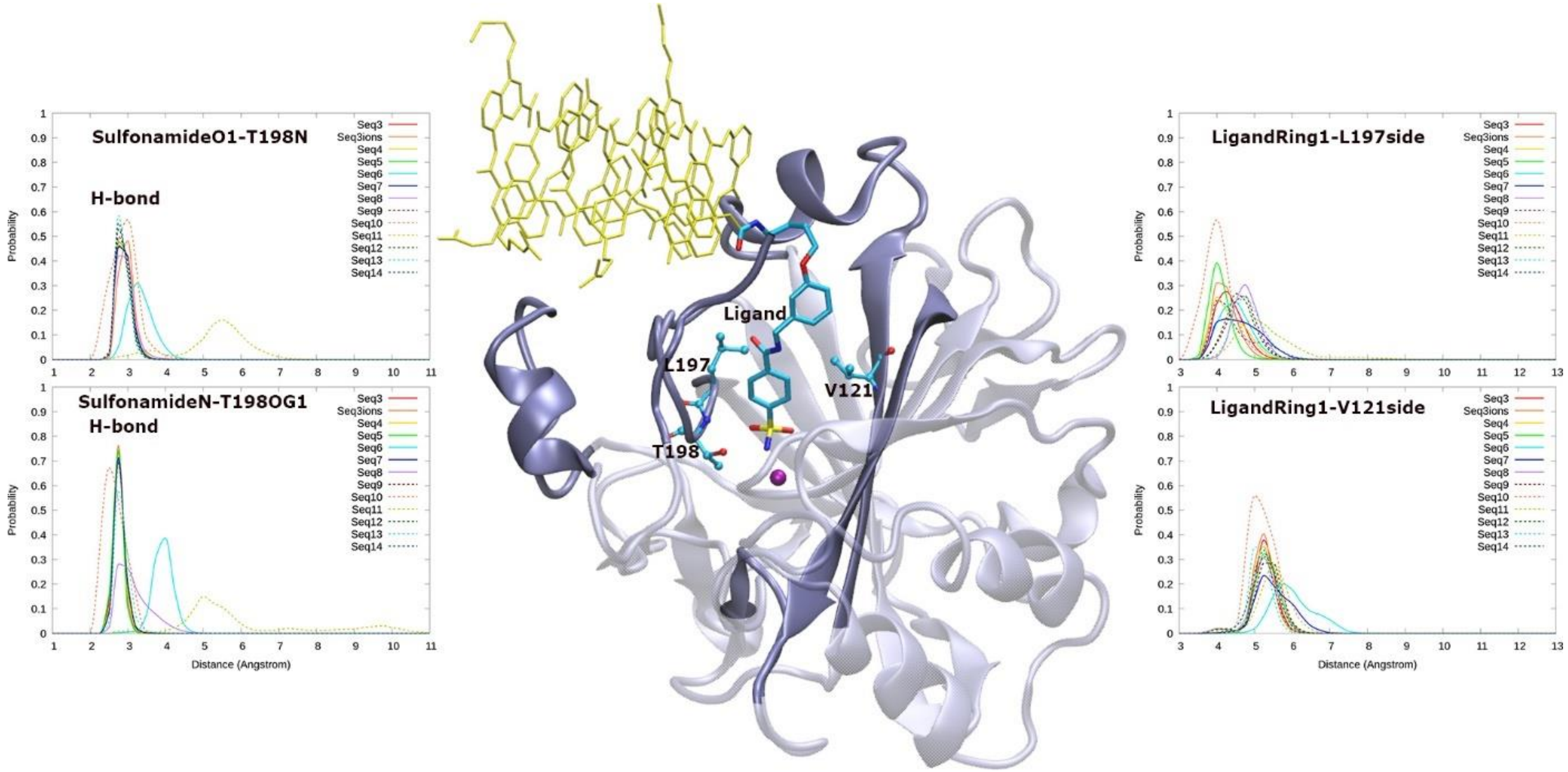

Figure S10. Interaction at pocket 1. Histograms of distances between ligand groups and protein residues indicating either hydrogen bond (H-bond) or hydrophobic interaction are calculated based on 500 ns trajectory.

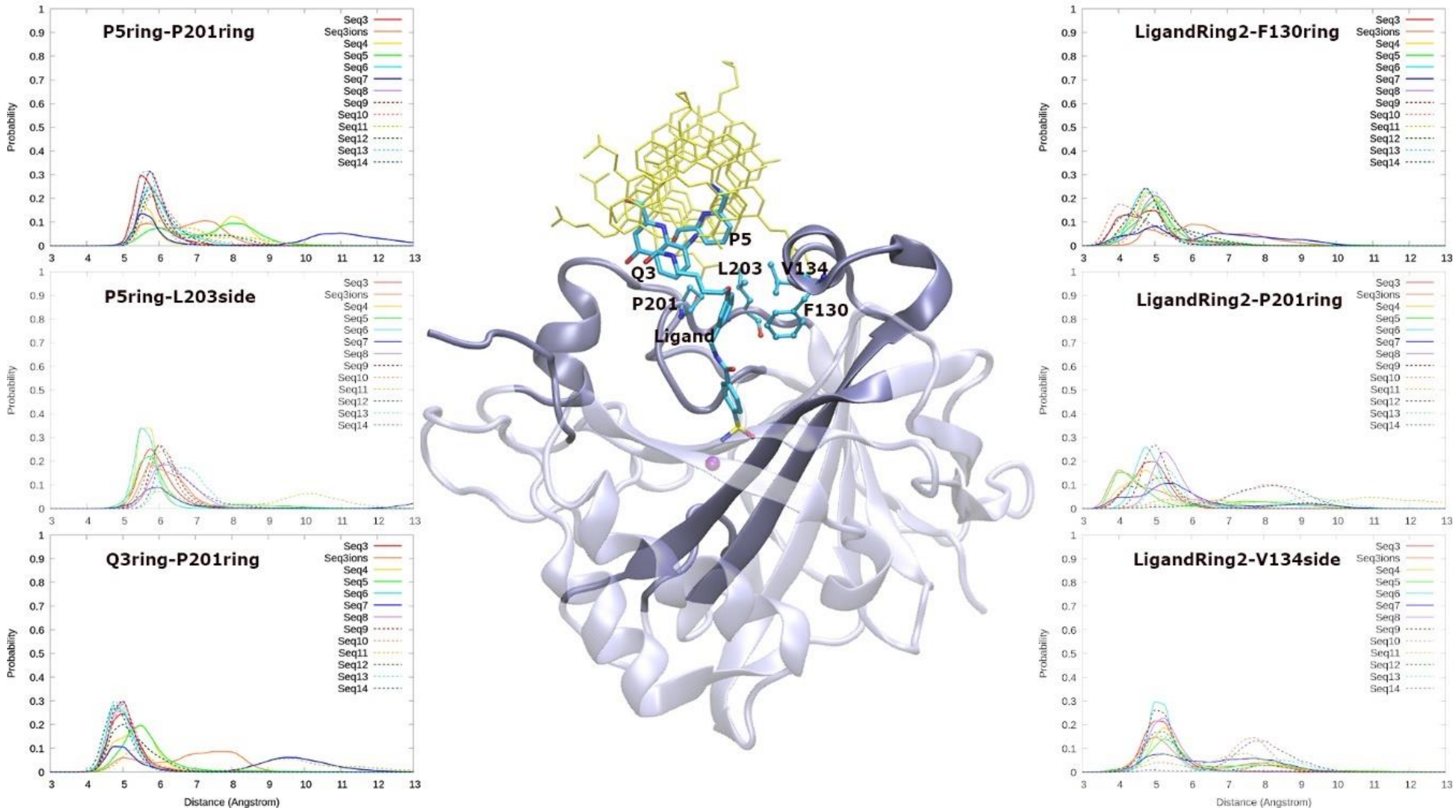

Figure S11. Interaction at pocket 2. Histograms of distances between ligand or foldamer groups and protein residues indicating hydrophobic interaction are calculated based on 500 ns trajectory. (Q3ringN indicates the aromatic ring on the N side of Q3).

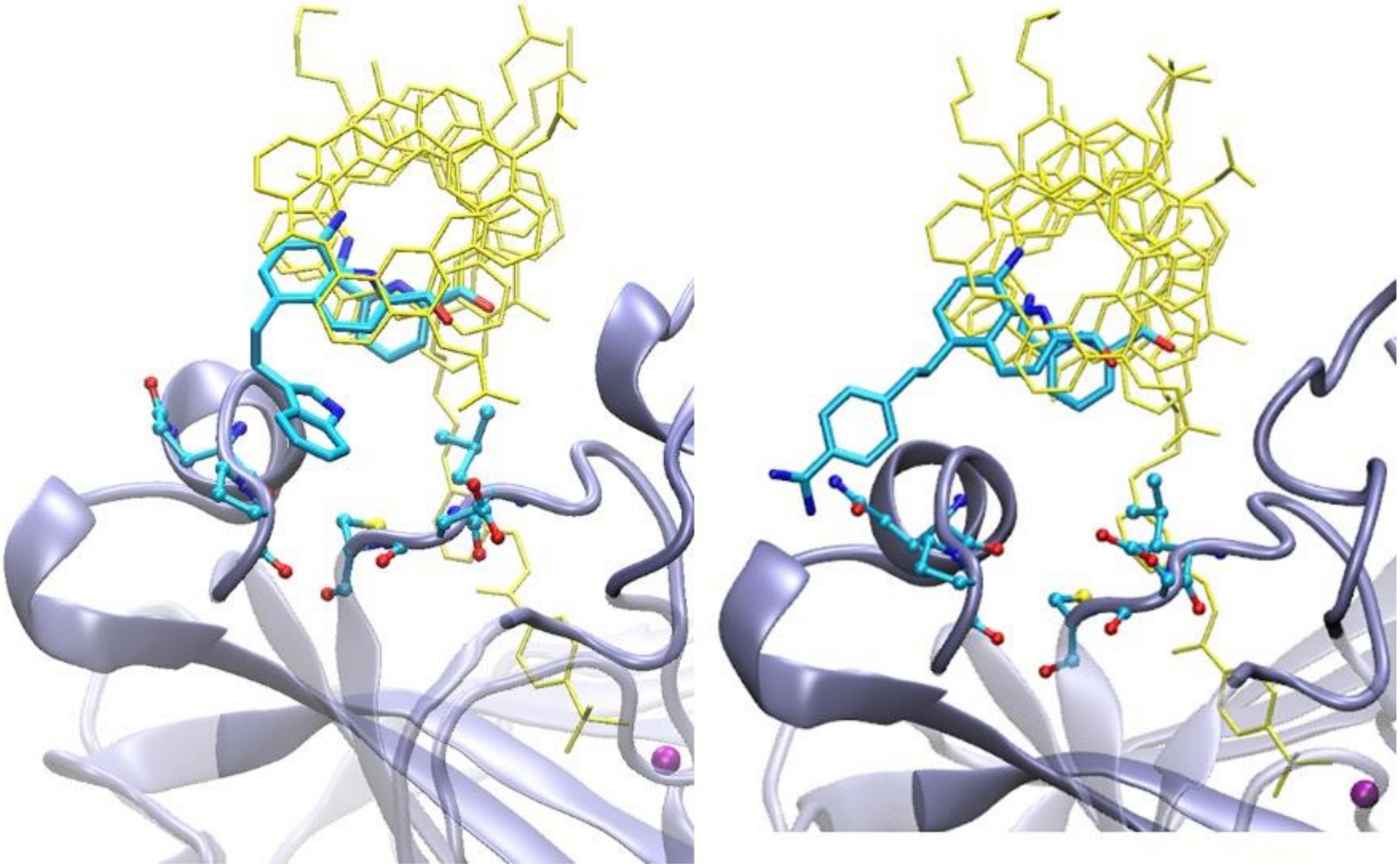

Figure S12. Snapshots of sequences **4*** (left) and **5*** (right) show differences in $Q^{5In}10$ and $Q^{5Pa}10$ side chain positions with respect to that of $Q^{5Ph}10$ in all other sequences (Fig. 5b).

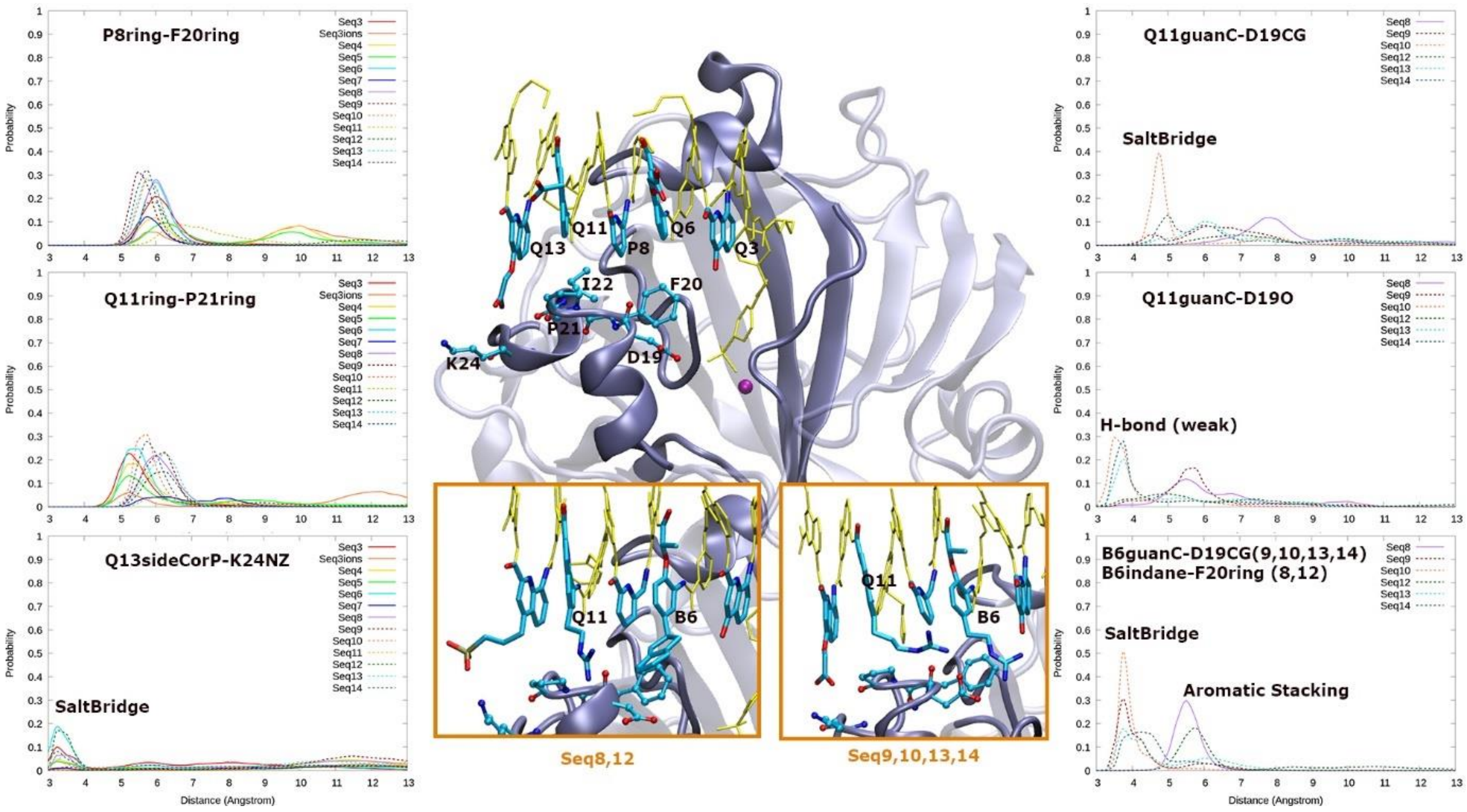

Figure S13. Interaction at pocket 3 or 6 or 7 or 8. Insertions with orange boarders are from different sequences with side chains on unit 6, 11, 13. Histograms of distances between foldamer groups and protein residues indicating salt bridge, H-bond or hydrophobic interactions are calculated based on 500 ns trajectory data.

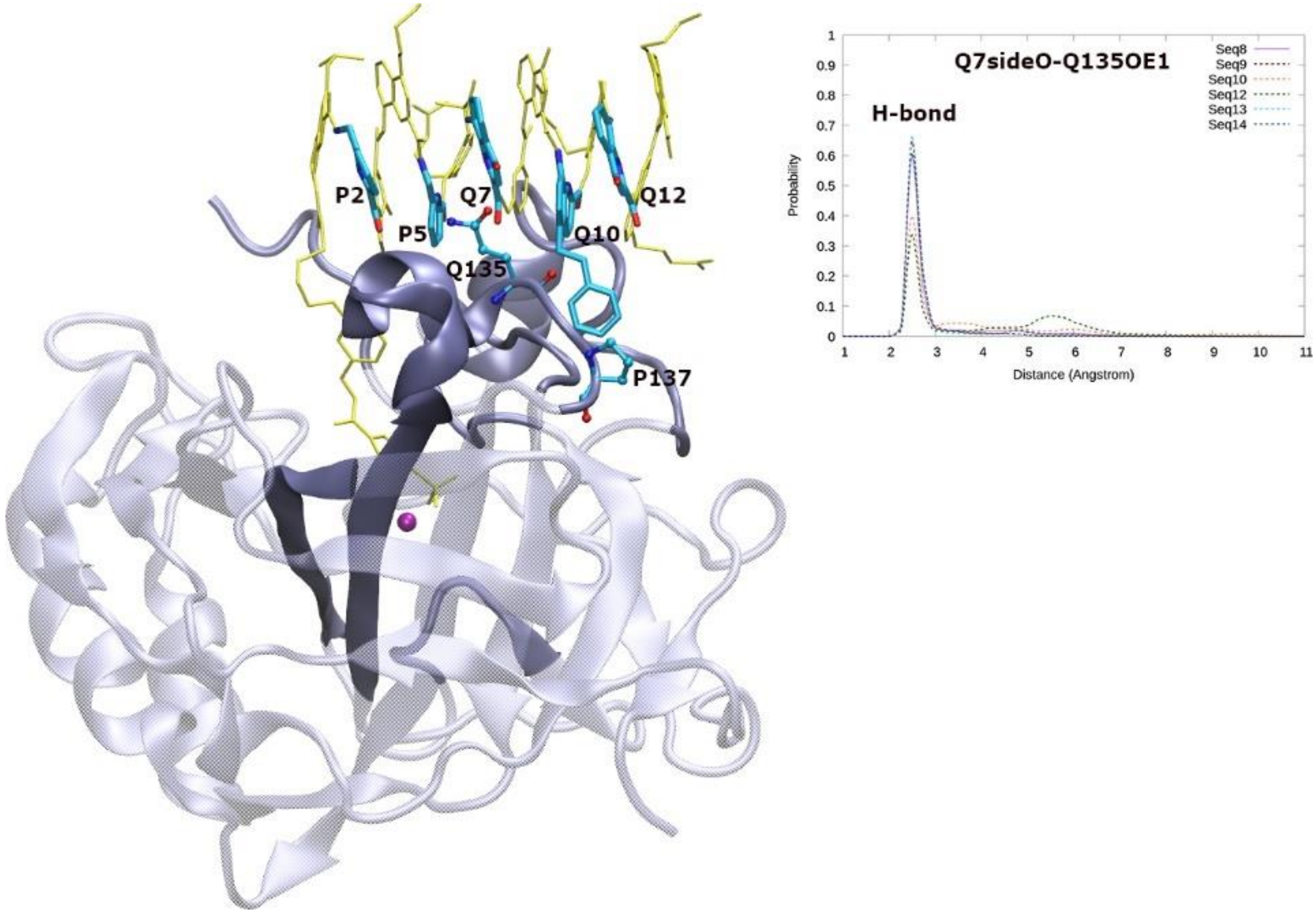

Figure S14. Interaction at pocket 10. Histograms of distances between Q7 sidechain O (of OH group on position 4) and GLN135OE1 indicating H-bond are calculated based on 500 ns trajectory data.

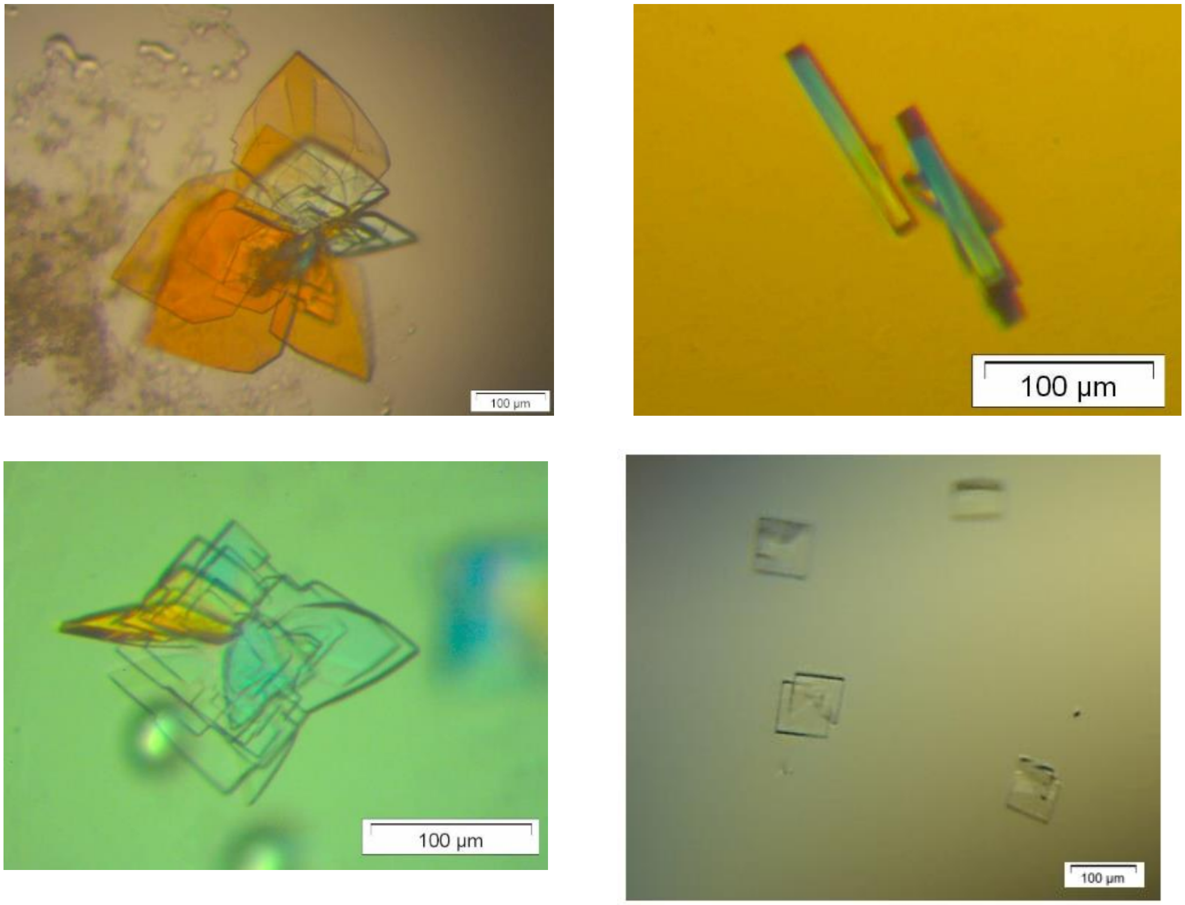

Figure S15. Crystals of complexes of HCAII and **2** (top left), **3** (top right), **16** (bottom left), **20** (bottom right)

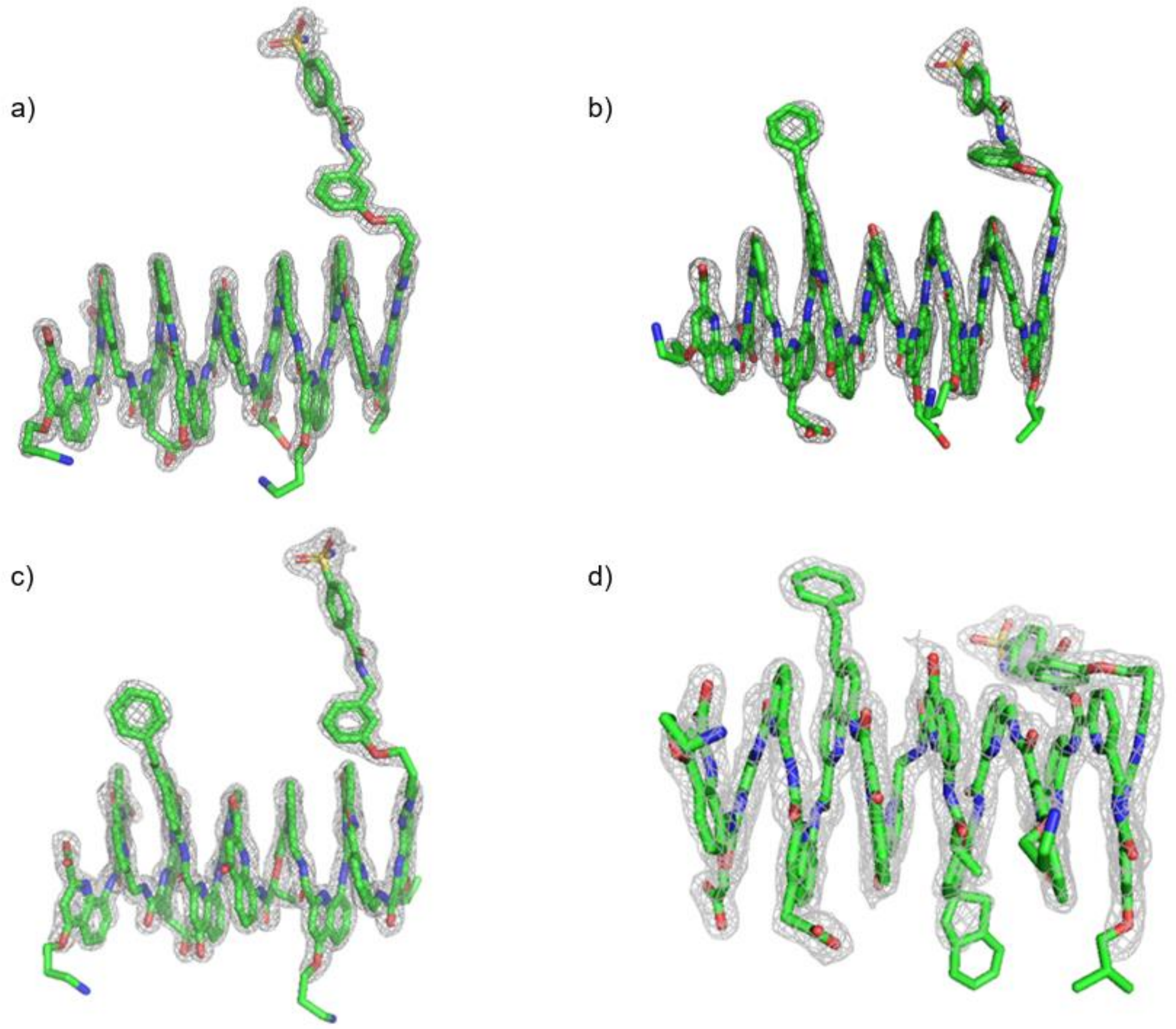

Figure S16. Electron density maps at 1.5 σ cut off of four foldamer helices when bound to HCAII in the solid-state, a): **2**, b): **3**, c): **16**, d): **20**.

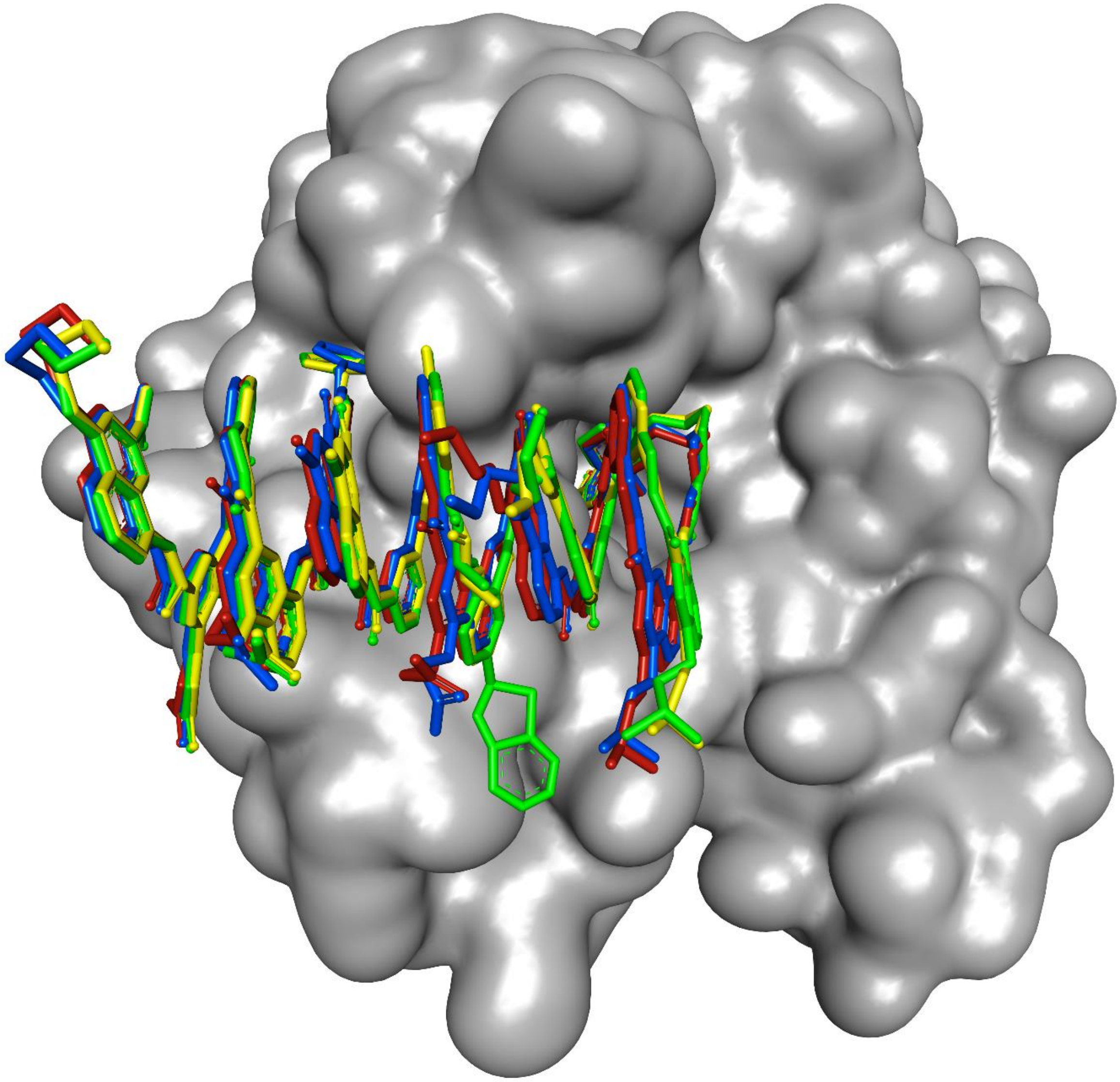

Figure S17: Overlay of foldamer sequences bound to HCAII as they are found in the solid state. The HCAII molecules have been superimposed. Sequence **2** is shown in red, sequence **3** in blue, sequence **16** in yellow, sequence **20** in green. The HCAII surface is colored in grey.

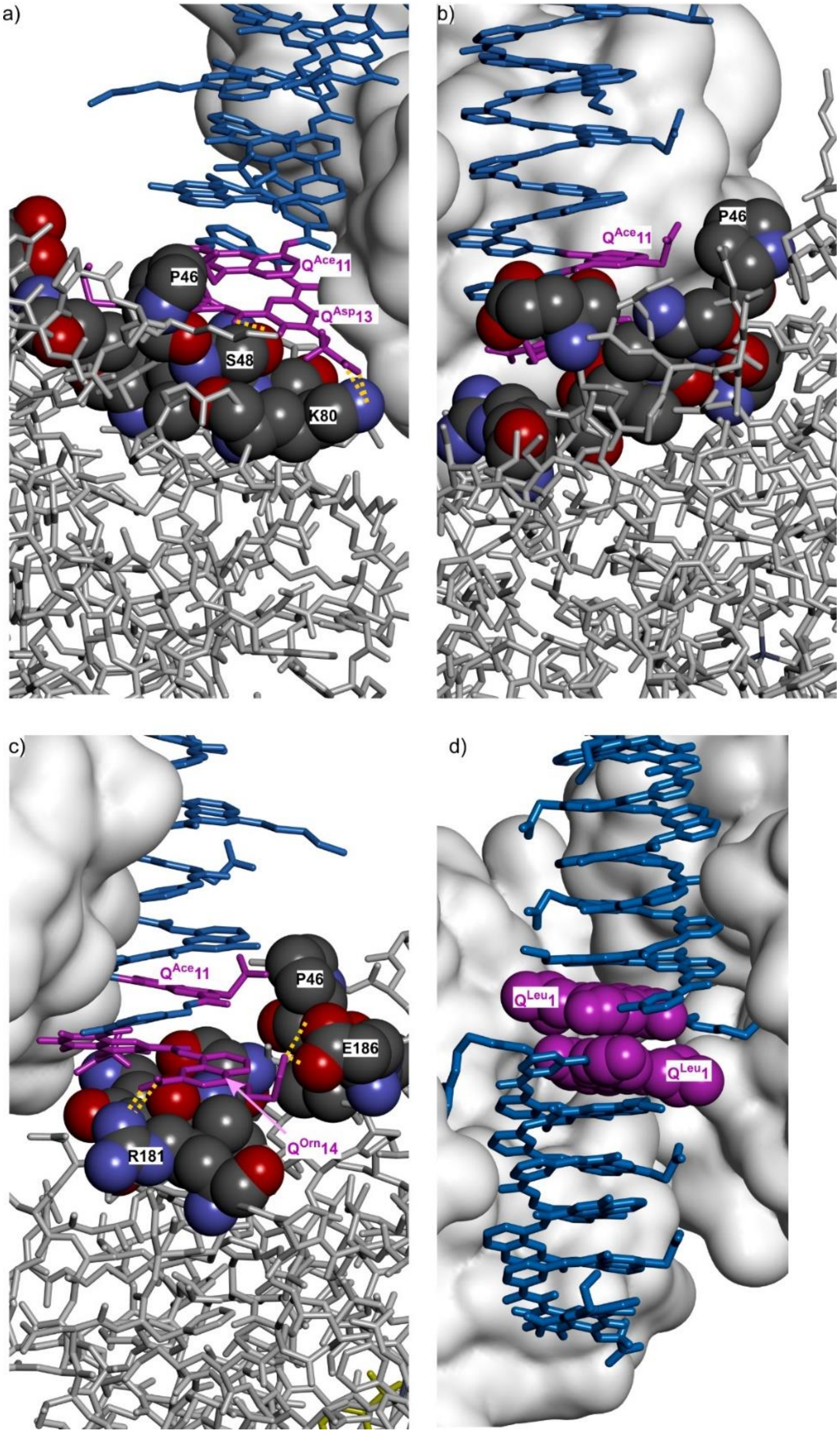

Figure S18. Intercomplex contacts involving the foldamer in the solid state structures

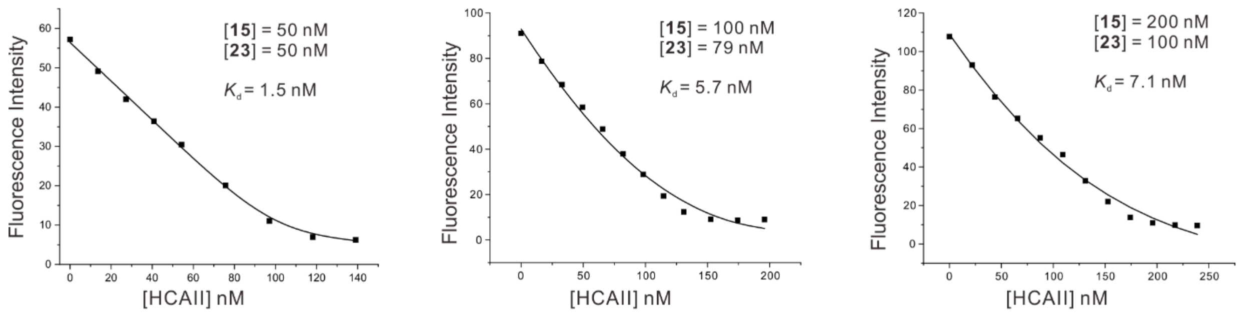

Figure S19. Experimental (■) and calculated (black line -) values for fluorescence intensity of HCAII titration to sequences **15** in different foldamer **15** and fluorescence probe **23** concentration combinations. Note that the curve fitting is shown at one wavelength (419 nm) as an illustration but that the K_D values were calculated by simultaneously fitting 380-600 nm wavelength measured.

1.2 Supplementary tables

Table S1. Screening results of unit 6

SMILES	Score (kcal/mol)
CC(=O)Nc1cc(C2Cc3ccccc3C2)ccc1OC(C)C(N)=O	-1.1
CC(=O)Nc1cc([C@@H](C)[C@@H](O)CO)ccc1OC(C)C(N)=O	-0.9
CC(=O)Nc1cc([C@@H](C)[C@@H](C)O)ccc1OC(C)C(N)=O	-0.8
CC(=O)Nc1cc(C[C@@H](O)CNC(N)=[NH2+])ccc1OC(C)C(N)=O	-0.8
CC(=O)Nc1cc([C@H](O)C(N)=O)ccc1OC(C)C(N)=O	-0.7
CC(=O)Nc1cc(CC(=O)c2ccccc2N)ccc1OC(C)C(N)=O	-0.7
CC(=O)Nc1cc(CCNC(N)=[NH2+])ccc1OC(C)C(N)=O	-0.7
CC(=O)Nc1cc(C[C@H](O)C(=O)O)ccc1OC(C)C(N)=O	-0.6
CC(=O)Nc1cc(C[C@H](O)C(N)=O)ccc1OC(C)C(N)=O	-0.6
CC(=O)Nc1cc(C[C@@H]2CN[C@H](F)N2)ccc1OC(C)C(N)=O	-0.6
CC(=O)Nc1cc([C@@H](C)CC(N)=O)ccc1OC(C)C(N)=O	-0.6
CC(=O)Nc1cc([C@@H](C)CC(=O)O)ccc1OC(C)C(N)=O	-0.6
CC(=O)Nc1cc([C@H](C)CC(=O)O)ccc1OC(C)C(N)=O	-0.6
CC(=O)Nc1cc(CCCCNC(N)=[NH2+])ccc1OC(C)C(N)=O	-0.6
CC(=O)Nc1cc(C[C@H](F)C(=O)O)ccc1OC(C)C(N)=O	-0.6
CC(=O)Nc1cc([C@H](O)CO)ccc1OC(C)C(N)=O	-0.5
CC(=O)Nc1cc(CCO)ccc1OC(C)C(N)=O	-0.5
CC(=O)Nc1cc([C@@H](C)CCBr)ccc1OC(C)C(N)=O	-0.5
CC[C@H](C)c1ccc(OC(C)C(N)=O)c(NC(C)=O)c1	-0.5
CC[C@@H](C)c1ccc(OC(C)C(N)=O)c(NC(C)=O)c1	-0.5
CC(=O)Nc1cc(CCc2ccccc2)ccc1OC(C)C(N)=O	-0.5
CC(=O)Nc1cc(CC(F)F)ccc1OC(C)C(N)=O	-0.5
CC(=O)Nc1cc(CCN)ccc1OC(C)C(N)=O	-0.5
CC(=O)Nc1cc([C@H](C)C(=O)O)ccc1OC(C)C(N)=O	-0.5
CC(=O)Nc1cc(CCC(=O)NO)ccc1OC(C)C(N)=O	-0.4
CNC(=O)CCc1ccc(OC(C)C(N)=O)c(NC(C)=O)c1	-0.4
CC(=O)Nc1cc([C@H](C)C=O)ccc1OC(C)C(N)=O	-0.4
CC(=O)Nc1cc(CCC[C@@H](N)C(=O)O)ccc1OC(C)C(N)=O	-0.4

CC(=O)Nc1cc(CCON)ccc1OC(C)C(N)=O	-0.4
CC(=O)Nc1cc(C(C)C)ccc1OC(C)C(N)=O	-0.4
CC(=O)Nc1cc(CCC(=O)O)ccc1OC(C)C(N)=O	-0.4
CC(=O)Nc1cc(CCC(N)=O)ccc1OC(C)C(N)=O	-0.4
CC(=O)Nc1cc(C(O)O)ccc1OC(C)C(N)=O	-0.4
CC(=O)CCc1ccc(OC(C)C(N)=O)c(NC(C)=O)c1	-0.4
COC(=O)CCc1ccc(OC(C)C(N)=O)c(NC(C)=O)c1	-0.4
CC(=O)Nc1cc(CCON=C(N)N)ccc1OC(C)C(N)=O	-0.4
CC(=O)Nc1cc(CCCC(=O)C(=O)O)ccc1OC(C)C(N)=O	-0.4
CC(=O)Nc1cc(CCCC(=O)O)ccc1OC(C)C(N)=O	-0.4
CC(=O)Nc1cc(CCCNC(N)=S)ccc1OC(C)C(N)=O	-0.4
CC(=O)Nc1cc([C@H](O)C(C)C)ccc1OC(C)C(N)=O	-0.4
CC(=O)Nc1cc([C@@H](O)C(C)C)ccc1OC(C)C(N)=O	-0.4
CC(=O)Nc1cc(CCCCO)ccc1OC(C)C(N)=O	-0.4
CC(=O)Nc1cc(CCCCN)ccc1OC(C)C(N)=O	-0.4
C=Cc1ccc(OC(C)C(N)=O)c(NC(C)=O)c1	-0.3
CC(=O)Nc1cc([C@@H](C)O)ccc1OC(C)C(N)=O	-0.3
CC(=O)Nc1cc([C@H](C)O)ccc1OC(C)C(N)=O	-0.3
C=C(CCCc1ccc(OC(C)C(N)=O)c(NC(C)=O)c1)C(=O)O	-0.3
CC(=O)Nc1cc(CCCNC(N)=[NH2+])ccc1OC(C)C(N)=O	-0.3
CC(=O)Nc1cc(CCCNC(N)=O)ccc1OC(C)C(N)=O	-0.3
C=CCc1ccc(OC(C)C(N)=O)c(NC(C)=O)c1	-0.3
CC(=O)Nc1cc(CCCCCCN)ccc1OC(C)C(N)=O	-0.3
CCc1ccc(OC(C)C(N)=O)c(NC(C)=O)c1	-0.3
CC(=O)Nc1cc(C[C@@H](C)C=O)ccc1OC(C)C(N)=O	-0.3
CCC(CC)c1ccc(OC(C)C(N)=O)c(NC(C)=O)c1	-0.3
CC(=O)Nc1cc(CC=O)ccc1OC(C)C(N)=O	-0.3
CC[C@@H](O)c1ccc(OC(C)C(N)=O)c(NC(C)=O)c1	-0.3
CC(=O)Nc1cc(C[C@@H](C)C(=O)O)ccc1OC(C)C(N)=O	-0.3
CC(=O)Nc1cc(C[C@H](C)C(F)(F)F)ccc1OC(C)C(N)=O	-0.3
CCCCCc1ccc(OC(C)C(N)=O)c(NC(C)=O)c1	-0.3
CC(=O)Nc1cc(CNC(N)=[NH2+])ccc1OC(C)C(N)=O	-0.3
CC(=O)Nc1cc(CCCO)ccc1OC(C)C(N)=O	-0.3

CC(=O)Nc1cc(CCSC(F)F)ccc1OC(C)C(N)=O	-0.3
CC(=O)Nc1cc(CO)ccc1OC(C)C(N)=O	-0.2
CCCc1ccc(OC(C)C(N)=O)c(NC(C)=O)c1	-0.2
CCCCc1ccc(OC(C)C(N)=O)c(NC(C)=O)c1	-0.2
CC(=O)Nc1cc(CCCN)ccc1OC(C)C(N)=O	-0.2
CC(=O)Nc1cc(CCSCO)ccc1OC(C)C(N)=O	-0.2
CC(=O)Nc1cc(C[C@H](C)CF)ccc1OC(C)C(N)=O	-0.2
CC(=O)Nc1cc(CN)ccc1OC(C)C(N)=O	-0.2
CC(=O)Nc1cc(CCSC#N)ccc1OC(C)C(N)=O	-0.2
CC(=O)Nc1cc(CC2CCCC2)ccc1OC(C)C(N)=O	-0.2
CC(=O)Nc1cc([C@H](O)CCl)ccc1OC(C)C(N)=O	-0.2
CC(=O)Nc1cc([C@H](O)C(=O)O)ccc1OC(C)C(N)=O	-0.2
CCSCCc1ccc(OC(C)C(N)=O)c(NC(C)=O)c1	-0.2
CC(=O)Nc1cc(C(F)(F)F)ccc1OC(C)C(N)=O	-0.2
CC(=O)Nc1cc(CC(C)C)ccc1OC(C)C(N)=O	-0.2
CC(=O)Nc1cc(CC(=O)O)ccc1OC(C)C(N)=O	-0.1
CC(=O)Nc1cc(C)ccc1OC(C)C(N)=O	-0.1
CC(=O)Nc1cc(CC[C@H]2C=C[C@@H](N)C=C2)ccc1OC(C)C(N)=O	-0.1
CC(=O)Nc1cc(CC2CCCCC2)ccc1OC(C)C(N)=O	-0.1
CSCCc1ccc(OC(C)C(N)=O)c(NC(C)=O)c1	-0.1
CC(=O)Nc1cc(CCl)ccc1OC(C)C(N)=O	-0.1
CO[C@H](C)c1ccc(OC(C)C(N)=O)c(NC(C)=O)c1	-0.1
CC(=O)Nc1cc(CCS)ccc1OC(C)C(N)=O	-0.1

Table S2.Screening results of unit 10

**SMILES**	**Score (kcal/mol)**
CC(=O)Nc2ccc(CCc1ccccc1)c3ccc(C(N)=O)nc23	-1.4
CC(=O)Nc2ccc(CCc1ccc(Cl)cc1)c3ccc(C(N)=O)nc23	-1.7
CC(=O)Nc2ccc(CCc1ccc(C(N)=[NH2+])cc1)c3ccc(C(N)=O)nc23	-2.2
CC(=O)Nc2ccc(CCc1ccc(O)cc1)c3ccc(C(N)=O)nc23	-1.6
COc3ccc(CCc1ccc(NC(C)=O)c2nc(C(N)=O)ccc12)cc3	-1.7

CC(=O)Nc3ccc(CCc1c[nH]c2ccccc12)c4ccc(C(N)=O)nc34	-2.0
CC(=O)Nc3ccc(CCc1c[nH]c2cc(Cl)ccc12)c4ccc(C(N)=O)nc34	-2.1
CC(=O)Nc3ccc(CCc1c[nH]c2c(O)c(N)ccc12)c4ccc(C(N)=O)nc34	-2.5

Table S3: Screening results of unit 11

SMILES	Score (kcal/mol)
CC(=O)Nc1cc(CC[C@H](C)NC(N)=[NH2+])cc2ccc(C(N)=O)nc12	-1.1
CC(=O)Nc1cc(CCC(=O)NO)cc2ccc(C(N)=O)nc12	-1.0
CC(=O)Nc1cc(CC[C@H]2C=C[C@@H](N)C=C2)cc2ccc(C(N)=O)nc12	-1.0
C=C(CCCc1cc(NC(C)=O)c2nc(C(N)=O)ccc2c1)C(=O)O	-0.9
CC(=O)Nc1cc(CC=Cc2ccccc2)cc2ccc(C(N)=O)nc12	-0.9
CC(=O)Nc1cc(CCNC(N)=[NH2+])cc2ccc(C(N)=O)nc12	-0.9
CC(=O)Nc1cc(CCCNC(N)=[NH2+])cc2ccc(C(N)=O)nc12	-0.9
CC(=O)Nc1cc(CCC[C@@H](N)C(=O)O)cc2ccc(C(N)=O)nc12	-0.9
CC(=O)Nc1cc(CCCNC(N)=O)cc2ccc(C(N)=O)nc12	-0.8
CC(=O)Nc1cc(CCCCNC(N)=[NH2+])cc2ccc(C(N)=O)nc12	-0.8
CC(=O)Nc1cc(CCCNC(N)=S)cc2ccc(C(N)=O)nc12	-0.8
CNC(=O)CCc1cc(NC(C)=O)c2nc(C(N)=O)ccc2c1	-0.8
CC(=O)Nc1cc(CCCN)cc2ccc(C(N)=O)nc12	-0.7
CC(=O)Nc1cc(CCCC(=O)C(=O)O)cc2ccc(C(N)=O)nc12	-0.7
CC(=O)Nc1cc(CCCO)cc2ccc(C(N)=O)nc12	-0.7
CC(=O)Nc1cc(CCCCN)cc2ccc(C(N)=O)nc12	-0.7
CC(=O)Nc1cc(CCCCCCN)cc2ccc(C(N)=O)nc12	-0.7
CC(=O)Nc1cc(CCON=C(N)N)cc2ccc(C(N)=O)nc12	-0.7
COC(=O)CCc1cc(NC(C)=O)c2nc(C(N)=O)ccc2c1	-0.7
CC(=O)Nc1cc(CCC(C)C)cc2ccc(C(N)=O)nc12	-0.7
CC(=O)CCc1cc(NC(C)=O)c2nc(C(N)=O)ccc2c1	-0.6
CC(=O)Nc1cc(CCCC(=O)O)cc2ccc(C(N)=O)nc12	-0.6
CC(=O)Nc1cc(CCC(=O)O)cc2ccc(C(N)=O)nc12	-0.6
CC(=O)Nc1cc(CCCCO)cc2ccc(C(N)=O)nc12	-0.6
CCCCCc1cc(NC(C)=O)c2nc(C(N)=O)ccc2c1	-0.6

CC(=O)Nc1cc(CCC(N)=O)cc2ccc(C(N)=O)nc12	-0.6
CC(=O)Nc1cc(CCON)cc2ccc(C(N)=O)nc12	-0.6
CC(=O)Nc1cc(CCS(C)(=O)=O)cc2ccc(C(N)=O)nc12	-0.6
CC(=O)Nc1cc(CCSC(F)F)cc2ccc(C(N)=O)nc12	-0.6
CCCCc1cc(NC(C)=O)c2nc(C(N)=O)ccc2c1	-0.6
CC(=O)Nc1cc(CCSCO)cc2ccc(C(N)=O)nc12	-0.5
CC(=O)Nc1cc(CC[S@@](C)=O)cc2ccc(C(N)=O)nc12	-0.5
CC(=O)Nc1cc(CC[S@@](C)=O)cc2ccc(C(N)=O)nc12	-0.5
CC(=O)Nc1cc(CCSC#N)cc2ccc(C(N)=O)nc12	-0.5
CC(=O)Nc1cc(CCN)cc2ccc(C(N)=O)nc12	-0.5
CC(=O)Nc1cc(CCO)cc2ccc(C(N)=O)nc12	-0.5
CCSCCc1cc(NC(C)=O)c2nc(C(N)=O)ccc2c1	-0.4
CCCc1cc(NC(C)=O)c2nc(C(N)=O)ccc2c1	-0.4
C=CCc1cc(NC(C)=O)c2nc(C(N)=O)ccc2c1	-0.4
CSCCc1cc(NC(C)=O)c2nc(C(N)=O)ccc2c1	-0.4
CC(=O)Nc1cc(CCc2ccccc2)cc2ccc(C(N)=O)nc12	-0.4
CC(=O)Nc1cc(CCS)cc2ccc(C(N)=O)nc12	-0.4
CCc1cc(NC(C)=O)c2nc(C(N)=O)ccc2c1	-0.3
CC(=O)Nc1cc(CC=O)cc2ccc(C(N)=O)nc12	-0.3

Table S4 Data-collection and refinement statistics (Statistics for the highest-resolution shell are shown in parentheses.)

	**3•HCAII**	**2•HCAII**	**16•HCAII**	**20•HCAII**
**Data collection**				
X-ray source	SOLEIL-Proxima-2	SOLEIL-Proxima-2	ESRF- ID30A-1	ESRF- ID30B
Wavelength (Å)	0.979995	0.979999	0.965459	0.87313
Resolution (Å) (last shell)	50.00-2.11 (2.24-2.11)	65.00-1.40 (1.48-1.40)	45.89-1.64 (1.74-1.64)	17.17-2.05 (2.12-2.05)
Space Group	$P\ 2_1\ 2_1 2$	$P\ 2_1\ 2_1 2$	$P\ 2_1\ 2_1 2$	$P\ 2_1\ 2_1 2$
Cell parameters				
a, b, c (Å)	80.25, 81.29, 46.12	79.596, 81.601, 46.72	78.512, 81.515, 45.893	81.6816, 78.9698, 46.116
α, β, γ (°)	90.00, 90.00, 90.00	90.00, 90.00, 90.00	90.00, 90.00, 90.00	90.00, 90.00, 90.00
Asymmetric unit	1 complex	1 complex	1 complex	1 complex
Unique reflexions	17876 (2756)	60722 (9648)	67930 (10792)	19166 (3555)
Multiplicity	6.50 (6.57)	13.27 (13.19)	2.43 (2.31)	4.4 (4.60)
Completeness (%)	99.8 (97.1)	99.9 (99.6)	97.0 (95.7)	99.04 (97.20)
I/σ(I)	10.79 (1.22)	18.31 (1.17)	7.34 (0.51)	7.20 (1.88)
Rmeas (%)	11.7 (145.9)	7.3 (230.2)	9.2 (212.6)	16.8 (55.31)
$CC_{1/2}$ (%)	99.8 (55.7)	100 (58.8)	99.8 (22.9)	96 (80.1)
**Refinement**				
Rwork	0.2003 (0.366)	0.1555 (0.391)	0.1836 (0.412)	0.1708 (0.2056)
Rfree	0.2524 (0.402)	0.1825 (0.395)	0.2108 (0.385)	0.2309 (0.2937)
r.m.s. bonds (Å)	0.007	0.009	0.009	0.008
r.m.s. angles (°)	1.035	1.425	1.025	2.018
No. of atoms				
Total	2410	2649	2591	2649
protein	2020	2056	2073	2036
ligand	235	227	233	242
water	102	341	278	362
Overall B-factor (Å2)	50.084	22.393	31.218	25.15

protein	53.522	25.045	34.907	23.02
ligand	43.355	20.683	29.174	22.81
water	55.946	37.165	42.376	31.91
Ramachandran favored (%)	96.47	96.86	96.50	96.47
Molprobity score	1.26	0.91	1.23	1.57
**PDB code**	**9GAK**	**9GAM**	**9GAJ**	**9HGB**

2. Computational studies

2.1 Pocket analysis and pocket-centric side chain screening

Pocket analysis is performed using AlphaSpace 2.0,[1] which detects and quantitatively evaluates concave space on the surface of a protein or protein-ligand complex. In the analysis results, pocket space is a geometric feature related to the size and shape of a pocket, and Bscore measures the optimal amount of free energy that can be gained by occupying a pocket. The procedure of side chain screening is as follows: to increase pocket occupancy, pocket-centric screening is carried out to select suitable side chains. Side chains are either manually designed or selected from SwissSideChain (https://www.swisssidechain.ch/). Side chains are attached to the foldamer backbone unit which is located near the pocket of interest. Conformers are generated using RDKit (https://www.swisssidechain.ch/) by keeping the backbone atoms fixed. AutoDock Vina[2] minimization procedure is performed to calculate the binding affinity of each conformer and the highest binding affinity value is used as the score of the side chain.

Side chain screening is performed for pocket 4, pocket 6, and pocket 7 and 8 with side chain alternatives attached to unit 6, unit 10 and unit 11 respectively. For unit 10, the side chain candidates are manually designed; while for unit 6 and unit 11, the candidates are from SwissSideChain.

2.2 Molecular dynamics simulations

We carried out MD simulations on sequence **3** and **4*** to **14*** bound to HCAII (with Zn^{2+}) in explicit water using the AMBER22 package.[3] We built the initial structures of all systems based on the HCAII-**3** crystal structure. Basically, the initial structure of HCAII was directly adopted from the crystal structure and then the foldamer helix was positioned by aligning its backbone with that of HCAII-**3** crystal structure. The foldamer structure was constructed by connecting structurally pre-tuned arylamide building blocks and residues for the ligand. All building blocks/residues are created using a multi-conformational RESP fitting protocol.[4,5] Each system was then solvated by explicit TIP3P water molecules in a periodic box measuring about 82 Å along each side. The ff14SB force field[6] was used for α-amino acid residues. The general AMBER force field (GAFF),[7] with improved torsional parameters for arylamide,[4] was used for the foldamer. All systems were equilibrated using the same procedure involving solvent minimization, heating and NPT simulation at 1 atm and 300K. Production runs using the NVT ensemble at 300K were then carried out for 500 ns per system. Weak constraints were put in place to constrain the distance between Zn^{2+} to

the tele-N, tele-N and pros-N of His residues 94, 96, 119, respectively and the nitrogen of the sulfonamide group of the ligand, to make sure the ligand binds properly.

3. Crystallography

The recombinant HCAII enzyme was expressed and purified according to references.[8] Prior to crystallization, compounds **2**, **3**, **16** and **20** were solubilized in pure DMSO. HCAII (0.3 mM) was preincubated with 1.05 equiv. of foldamer **3**, with 1.05 equiv. of foldamer **2**, with 1.1 equiv. of foldamer **16** and with 1.05 equiv. of foldamer **20** in 50 mM Tris buffer (pH 7.8) containing 3 mM NaN_3.

HCAII–**2**: For the binary complex of HCAII with foldamer **2,** drops consisted of 0.5 µL of complex solution and 0.5 µL of the precipitant solution containing 0.2 M lithium sulfate, 0.1 M Tris (pH 8.5) PEG 4000 20%, and NaN_3 3 mM. The drops were equilibrated by vapor diffusion against the precipitant solution at room temperature, and platelets appeared after 2 to 4 weeks and grew to their final size (250 x 75 x 20 µm) within 1 to 2 months. They were cryo-protected in the precipitant solution supplemented by 33% glycerol.

HCAII–**3**: For the binary complex of HCAII with foldamer **3,** drops consisted of 0.4 µL of complex solution and 0.4 µL of the precipitant solution containing lithium sulfate 0.2 M, Tris 0.1 M pH 8.8, 18% PEG 4000, and NaN_3 3 mM. The drops were equilibrated by vapor diffusion against the precipitant solution at room temperature, and bladed crystals appeared after two days (125 × 75 × 17 µm). Theywere cryo-protected in the precipitant solution supplemented by 33% glycerol.

HCAII–**16**: For the binary complex of HCAII with foldamer **16**, drops consisted of 0.4 µL of complex solution and 0.4 µL of the precipitant solution containing 0.2 M lithium sulfate, 0.1 M Tris (pH 8.0) 24% PEG 4000, and 3 mM NaN_3. The drops were equilibrated by vapor diffusion against the precipitant solution at room temperature, and platelets appeared after 3 days (100 × 50 × 20 µm). They were cryo-protected in the precipitant after addition of one drop of LV CryoOil™.

HCAII–**20**: For the binary complex of HCAII with foldamer **20** drops consisted of 0.8 µL of complex solution and 0.8 µL of the precipitant solution containing 0.2 M lithium sulfate, 0.1 M Tris (pH 8.5), 30% PEG 4000, and 3 mM NaN_3. The drops were equilibrated by vapor diffusion against the precipitant solution at room temperature, and plate-shaped crystals appeared after 14 days (200

× 120 × 25 µm). They were cryo-protected in the precipitant after addition of 20% glycerol prior to flash freezing in liquid nitrogen.

Data were collected on microfocus beamline Proxima-2A at synchrotron SOLEIL for complexes HCAII–**3** and HCAII–**2**, at synchrotron ESRF on beamline ID30A-1 for complex HCAII–**16** and at ESRF beamline ID30B at 100 K for complex HCAII-**20**.

All data were reduced with XDS or CrysAlisPro (Rigaku Oxford Diffraction, (2024), CrysAlisPro Software system, version 1.171.43.130a, Rigaku Corporation, Wroclaw, Poland)[9] X-ray structures of HCAII-**2**, HCAII-**3**, HCAII-**16** were solved by molecular replacement using the program Phaser[10] and atomic coordinates of a previous protein/foldamer complex (PDB code 6QT9)[11] as a search model. The X-ray structure of HCAII-**20** was solved by molecular replacement using programs Phaser[10] and atomic coordinates of an apo-protein structure (PDB: 5EHV) [12] as a search model. Refinement was carried out using Refmac[13] and Phenix[14] and manual model building using Coot.[15] The topology files used to build and refine the modified inhibitors have been generated using Prodrg and Phenix eLBOW.[16] The X-ray structures were validated using Molprobity[17] prior to deposition in the RCSB Protein Data Bank (entry codes 9GAK, 9GAM, 9GAJ and 9HGB).

4. Biophysical measurements

4.1 CD and UV-Vis spectroscopy

Circular Dichroism (CD) spectra were recorded on a Jasco J-815 Circular Dichroism spectrometer using quartz cells of 2 mm optical path length. Scans were measured at 20°C, over a wavelength range of 300-500 nm, with a response time of 0.5 sec and a scanning speed of 50 nm/min. The CD data represents an average of two scans. All CD were baseline-corrected for signal contributions due to the buffer containing HCAII (HCAII 34.5 µM in 50 mM NaH_2PO_4 buffer at pH 7.4 or 6.0). Samples were prepared by adding 1 equiv. of the foldamer (10 mM solution in pure DMSO) to a solution containing HCAII (34.5 µM, in a 50 mM NaH_2PO_4 buffer at pH 7.4 or 6.0).

All ultraviolet–visible (UV/Vis) absorbance measurements were done with a Jasco V-750 spectrophotometer instrument using a 1 cm quartz cuvette. Measurements were performed at 20 °C if not stated otherwise.

A series of solutions of compound **23** (from 0.3 µM to 3.0 µM) were prepared in aqueous 50 mM HEPES buffer (pH 7.2), without degassing as previously described from a DMSO stock solution. The UV absorbance of **23** at 359 nm was shown in **Figure S20** and the whole UV spectrum was shown in **Figure S23**. From 2.0 µM to 3.0 µM (labelled in pink color), the absorbance decreases, which indicates a potential aggregation of **23**. The molar extinction coefficient (ε_{359nm}) was therefore calculated from Beer-Lambert's law and Abs values measured from a range of concentration of 0.3 µM to 2.0 µM. The linear regression gave a value of the ε_{359nm} of 26613 M^{-1} cm^{-1}, which is in good agreement with the reported value 27800 M^{-1} cm^{-1}.[18]

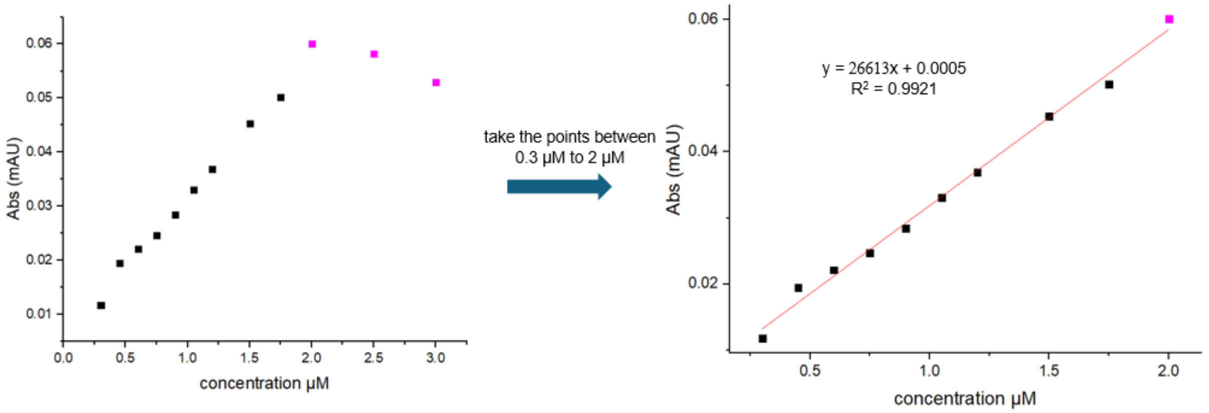

Figure S20. UV absorbance of **23** at 359 nm at different concentrations and UV calibration curve of **23** to compare with the value from literature (after removal of two Abs values above 2 µM).

4.2 Fluorescence binding assay

This fluorescence assay was adapted from the paper of Anzenbacher.[18] All fluorescence titrations were done in aqueous 50 mM HEPES buffer (pH = 7.2) at room temperature, without degassing of the samples and using a quartz fluorescence cuvette with 1 cm path length on Varian Cary Eclipse Spectrophotometer 05813. The titration assays were carried out in two parts: 1) sensor-protein titration (**Figure S21**); 2) sensor-foldamer competition assay (**Figure S24**). Several experiments were repeated and validated a reproducibility within 15% error.

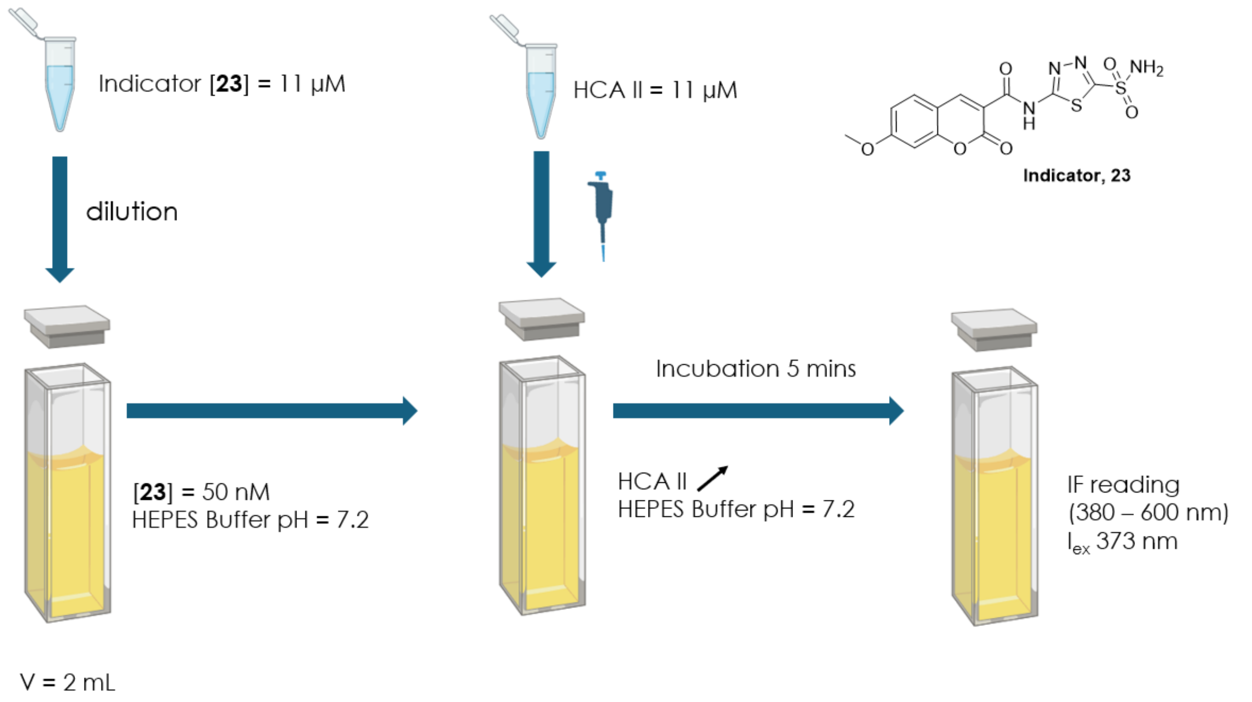

Figure S21. Schematic procedure of the titration experiment between indicator 23 and HCAII (adapted from literature[18]).

4.2.1 K_D measurement of the 23•HCAII complex

1. Fluorophore **23** was synthesized according to literature[18] and next dissolved in pure DMSO to get a stock solution at 3 mM.

2. To the solution of **23** (50 nM) in HEPES buffer (50 mM, pH 7.2) in the fluorescence cuvette (2 mL), incremental volumes of a stock solution of HCAII (11 µM) in HEPES buffer were added. After each addition and gentle agitation, the resulting mixture was incubated for 5 min at RT. The intensity of fluorescence (IF) was then recorded from 380 nm to 600 nm (λ_{ex} = 373 nm).

3. In total around 2.3 equiv. of protein was added (final concentration of protein in the cuvette was 114 nM) (see **Figure S22**). The data were plotted to calculate the K_D value using Hypspec

software.[19] For curve fitting, the binding model was set to 1:1. The spectra of all emitting species were recorded from 380 nm to 600 nm (λ_{ex} = 373 nm) in separate experiments and set as "unknown spectrum". The K_D value was determined considering all the wavelengths recorded. Errors quoted are standard deviations of the overall constants given directly by the program for the input data.

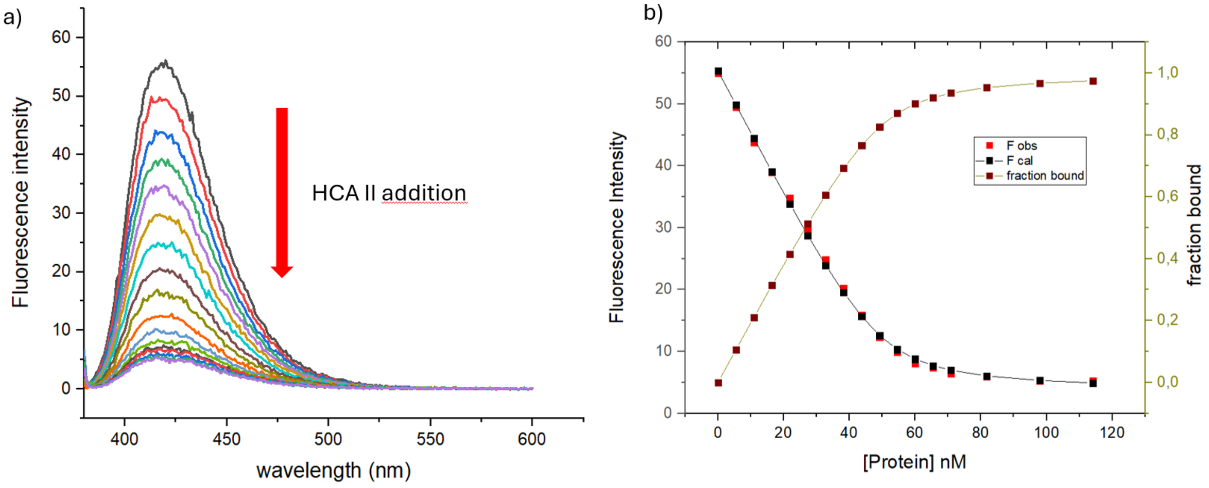

Figure S22. a) Fluorescence spectrum change upon HCAII addition; b) The intensity of fluorescence (IF) values read at λ_{em} =419 nm were plotted**.** The black squares are the read IF values and the red squares are the calculated IF. The overall fitting gives a K_D value for **23** of 1.69 nM, which is in good agreement with the K_D value reported,[18] and will next be used for the calculation of foldamer K_D values.

4.2.2 Sensor aggregation and reasons for protocol optimization

For foldamer binding affinity determination, we sought to implement a competitive fluorescence titration,[18] but performed the experiment after protocol optimization. In the published protocol, the competitors were added to a solution of HCAII-**23** mixture, and the IF change was recorded with increasing concentration of competitors. Titration curves could thus be obtained, and the K_D value of competitors could be calculated. In our experiment, we used foldamers as competitors and we opted not to add the foldamers to a solution of **23**-HCAII complex, but instead, we **added the protein to a solution of foldamer and 23** by preparing two solutions:

Solution 1 contained the foldamer and **23**

Solution 2 contained the protein, foldamer and **23** mixture (foldamer and **23** concentrations were the same as in ***solution 1***).

The ***solution 1*** was then titrated by ***solution 2,*** and the read emission spectra were used to calculate the K_D value of foldamers (competitors) by HypSpec software***.*** This experimental design was necessary to address the inner filter effect, which arises from two key factors. Firstly, since quinoline-based foldamers absorb at 373 nm (*i.e.* at the excitation wavelength of **23**), if high concentrations of foldamer were added to the solution of **23**-HCAII complex, the intensity of excitation light would be partly absorbed by the foldamers, leading to a decrease of emission light intensity of the **23** (primary inner filter effect).

Secondly, quinoline-based foldamers absorb in the wavelength range between 400-450 nm (UV spectrum of foldamer **3** is exemplified in **Figure S23**), overlapping with the emission spectrum of **23**. This inherent aromatic foldamer absorption might diminish the detected fluorescence intensity (secondary inner filter effect). By maintaining the foldamer and **23** concentrations constant during titration, this inner filter effect was kept constant, allowing changes in fluorescence to be attributed solely to the competitive interactions between the protein, **23**, and foldamers. We also worked at a low concentration of **23** and foldamers in the measurement and tested different concentration combinations to make sure the obtained values were reliable.

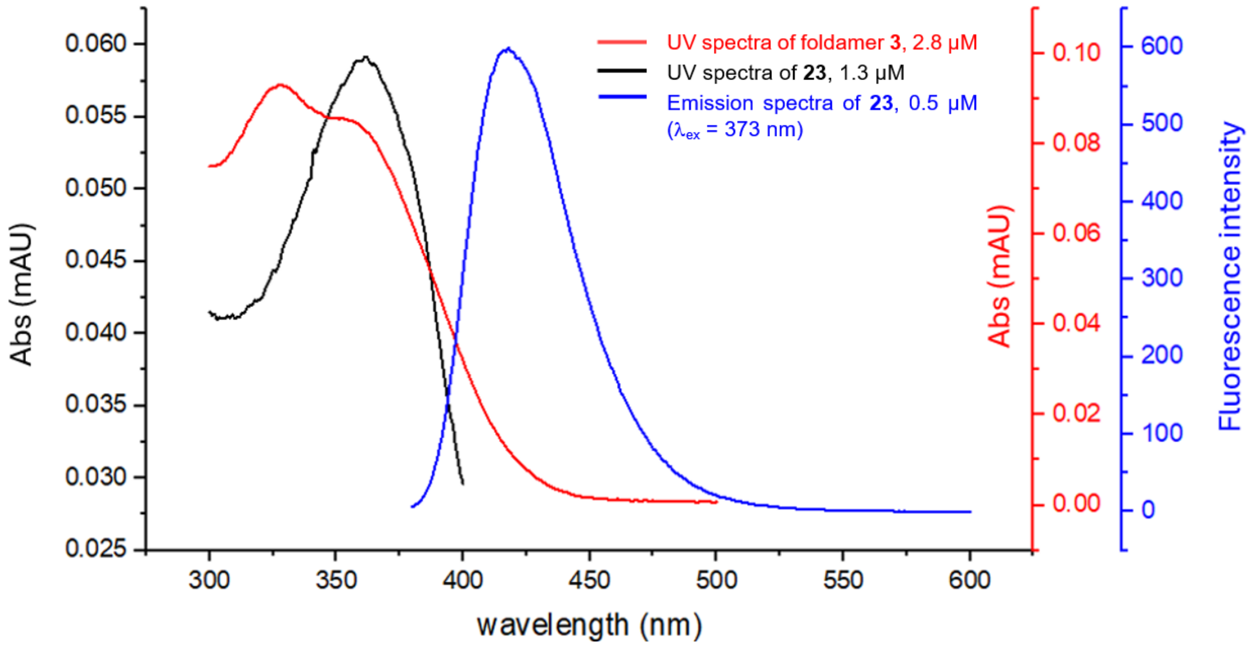

Figure S23. Overlay of UV-vis absorption spectra of foldamer **3** and probe **23**, fluorescence emission spectrum of **23** (λ_{ex} = 373 nm).

4.2.3 K_D measurement of HCAII complexes with AOFs 15-20 and ligand 22

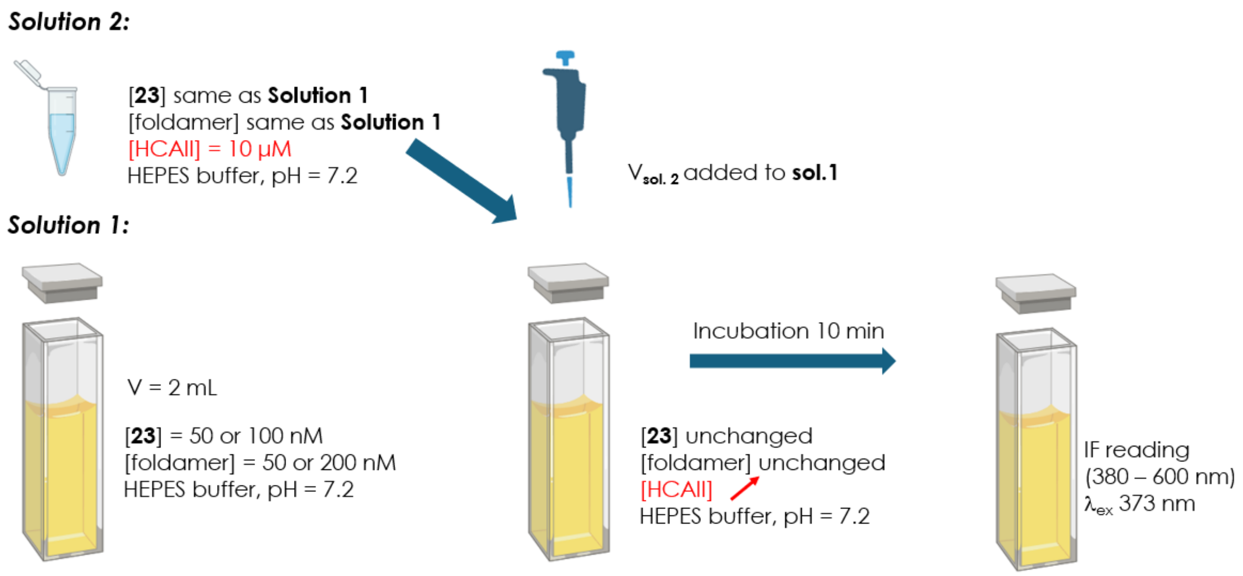

Figure S24. Schematic procedure of a competition experiment between **23** and foldamer (or ligand **22**).

1. ***Solution 1:*** **23** indicator stock solution and foldamer (or ligand **22**) stock solution were diluted in 50 mM HEPES buffer in the cuvette. The total volume was set at 2 mL.

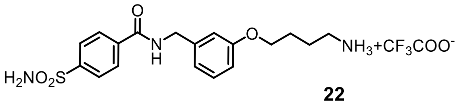

22

2. ***Solution 2***: Foldamer (or ligand **22**), **23** and protein (100 µM in HEPES buffer) stock solutions were diluted using 50 mM HEPES buffer. Concentration of foldamer and **23** were kept at 50 nM, (to remain identical to reference *solution 1*), final protein concentration was 20 µM; total volume was 100 µL. Using solution 2 allowed that the concentrations of foldamer and **23** were kept constant (no dilution) upon protein solution addition (λ_{ex} = 373 nm).

3. Aliquots of *solution 2* (1 to 3 µL) were added to *solution 1* and the IF was recorded from 380 nm to 600 nm (λ_{ex} = 373 nm).

4. As for curve fitting, a 1:1 binding model was applied. The K_D value of **23** (1.69 nM) was inserted as a constant. The spectra of all emitting species were recorded from 380 nm to 600 nm (λ_{ex} = 373 nm) in separate experiments and set as "known spectrum". The K_D values foldamer

were calculated considering all the wavelengths and the intensity of fluorescence at 419 nm is depicted.

4.3 BioLayer Interferometry

BLI measurements were carried out at 25 °C on an Octet R8 BLI Sartorius instrument, using streptavidin biosensors (SA). The buffer was 100 mM HEPES, 150 mM NaCl, 0.05% Tween, 0.1% DMSO, pH 7.4 (HEPES-D). At first and after a baseline in buffer for 120 sec, the biotinylated arylsulfonamide ligand **24** was loaded on the sensors (8 sensors, full column) with a loading at 4 μg/mL over 30 sec. The sensors were then washed with the buffer, a second baseline was recorded for 120 sec and then the association was performed with a range of HCAII concentrations from 50 nM to 0.78 nM for 240 sec before recording the dissociation over the same time in HEPES-D buffer. The K_D value was obtained after global curve fitting with the software embedded with the Octet R8 instrument.

5. Chemical Synthesis

5.1 General

Commercial reagents (suppliers: Abcr, Fisher Scientific, Merck, Sigma-Aldrich, TCI, BLDpharm or VWR) were used without further purification unless otherwise stated. LL Wang resin (100–200 mesh) was purchased from Sigma-Aldrich. Cl-MPA protide resin® was purchased from CEM-Germany. Peptide grade *N*,*N*-dimethylformamide (DMF) was purchased from Carlo Erba. Anhydrous chloroform, triethylamine (TEA) and *N*,*N*-diisopropylethylamine (DIPEA) were obtained via distillation over CaH_2 prior to use. Anhydrous tetrahydrofuran (THF) and dichloromethane (DCM) were obtained via an MBRAUN SPS-800 solvent purification system. Ultrapure water was collected on a Sartorius arium® pro VF ultrapure water system. Reactions were monitored by thin layer chromatography (TLC) on Merck silica gel 60-F254 plates and observed under UV light. Column chromatography purifications were carried out on Merck GEDURAN Si60 (40–63 µm). Nuclear magnetic resonance (NMR) spectra were recorded on an Avance III HD 400 MHz Bruker BioSpin spectrometer or an Avance III HD 500 MHz Bruker BioSpin spectrometer equipped with a broad band observe 5-mm BB-H&FD CryoProbeTM Prodigy. ^{1}H NMR measurements were performed at 25 °C unless stated otherwise. Water suppression was performed with excitation sculpting method. Processing was done with MestReNova (v.12.0.0-20080) NMR processing software from Mestrelab Research. Chemical shifts (δ) are reported in ppm and calibrated via residual solvent signals. Signal multiplicities are abbreviated as *s*, singlet; *d*, doublet; t, triplet; *q*, quartet, and *m*, multiplet. LC-MS spectra were recorded on a Bruker microTOF II in positive ionization mode. The instrument was calibrated in positive mode by direct infusion of a calibration solution (Agilent Technologies ESI-L Low Concentration Tuning Mix). The HPLC line was an Ultimate 3000 RP-HPLC system (ThermoFisher Scientific) equipped with a Nucleodur C18 gravity column (2 × 50 mm, 1.8 µm) at a flow rate of 0.33 mL/min. 0.2% formic acid and 0.02% TFA were added to the aqueous mobile phase (solvent A) and to acetonitrile (solvent B). The gradient was: 0-10 min, 10% to 100% solvent B at 50°C. The column eluent was monitored by UV detection at 214, 254, and 300 nm with a diode array detector. Analytical and semi-preparative reversed-phase (RP) high performance liquid chromatography (HPLC) were performed on a Thermo Fisher Scientific Ultimate 3000 HPLC System using MachereyNagel Nucleodur C18 Gravity columns (4 × 100 mm, 5 µm and 10 × 250 mm, 5 µm) or Macherey-Nagel Nucleodur C8 Gravity columns (4 × 50 mm, 5 µm and 10 × 100 mm, 5 µm) with different gradients composed of solvent A (0.1% TFA water) and B (0.1% TFA acetonitrile). Microwave-assisted solid phase foldamer synthesis (SPFS) was performed with a

CEM® Discover Bio manual microwave apparatus. The temperature within the reactor vessel was monitored with an optical fiber probe. Automated SPFS was done on a PurePep® Chorus synthesizer (Gyros Protein Technologies) by applying induction heating.

5.2 Experimental procedures for chemical synthesis and purification

5.2.1 Solid phase synthesis and purification of aromatic oligoamide foldamers (AOFs)

The loading of the first Fmoc-Q-OH monomer and loading determination were done according to reported protocols using Cl-MPA protide resin.[20] The Q, P, B monomers[21,22] were iteratively coupled on solid support as recently reported[23] using the PurePep® Chorus synthesizer. For ligand coupling see chapter 5.1.2. Cleavage from the resin and sidechain deprotection were performed simultaneously with a freshly prepared TFA solution containing triisopropylsilane (TIS) and water (TFA/TIS/H_2O, 95:2.5:2.5, v/v/v). After cleavage, the crude material was lyophilized and purified by semi-preparative RP-HPLC using a solvent mixture of A and B to furnish target foldamer sequences with purity over 95%.

5.2.2 Attachment of the Ligand and the DEG tail on resin-bound AOFs

This coupling step was optimized from the previously reported protocol.[24] The Fmoc-protected resin-bound 14mer (10 µmol scale) was first subjected to 20% piperidine in NMP for 5 min, washed with NMP (3 × 3 mL), and this step was repeated once. Then the resin was suspended in anhydrous THF (0.75 mL), followed by dry DIPEA (17 µL, 100 µmol, 10 equiv.). Triphosgene (15 mg. 50 µmol, 5 equiv.) was dissolved in anhydrous THF (0.75 mL), poured into the reaction vessel (RV), the RV was placed in the microwave and the resin was subsequently heated under microwave irradiations (50°C, 25 W, 5 min). The resin was next filtered off, washed with dry THF (5 × 3 mL) to remove any trace of triphosgene.

On the day preceding the ligand installation on solid phase, compound **45**[24](scheme 1) (24 mg, 50 µmol, 5 equiv.) was dissolved in a DCM/TFA mixture (1:1, v/v) to remove the Boc group and after solvent evaporation placed overnight under the vacuum line to remove traces of TFA. Then, the next day the resulting TFA salt **22** was dissolved in dry NMP (0.75 mL), followed by the addition of freshly distilled DIPEA (17 µL, 100 µmol, 10 equiv.), and the solution was poured in the RV containing the freshly activated isocyanate resin. The resin was heated up under microwave irradiations (50°C, 25 W, 15 min). The resin was next washed with anhydrous THF (5 × 3 mL), and this step was repeated once in the presence **22** and DIPEA.

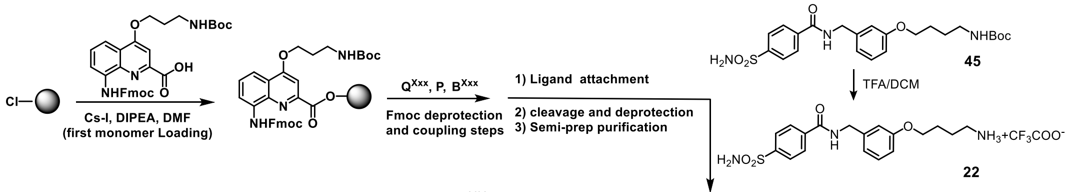

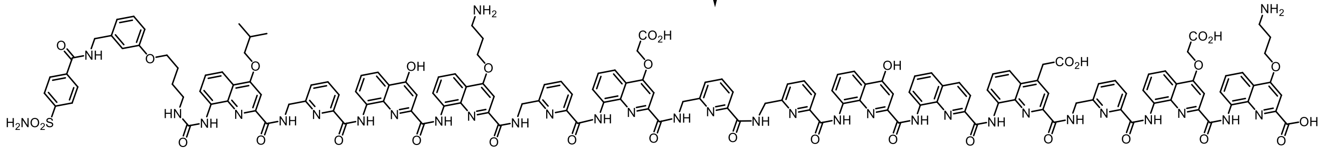

2

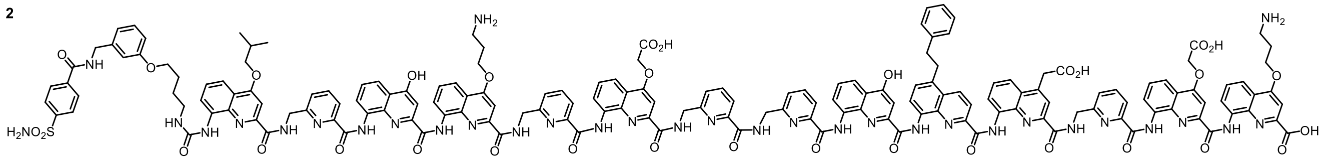

3

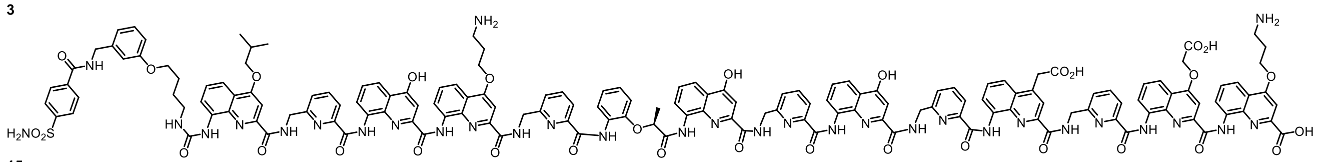

15

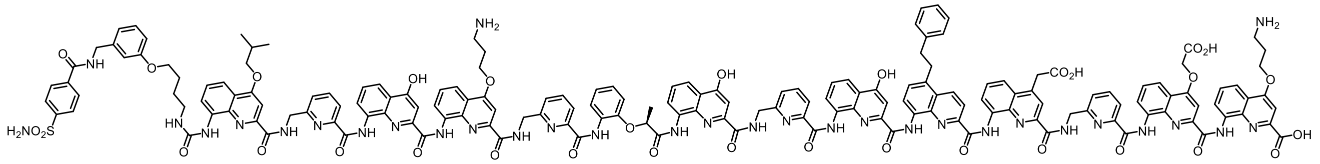

16

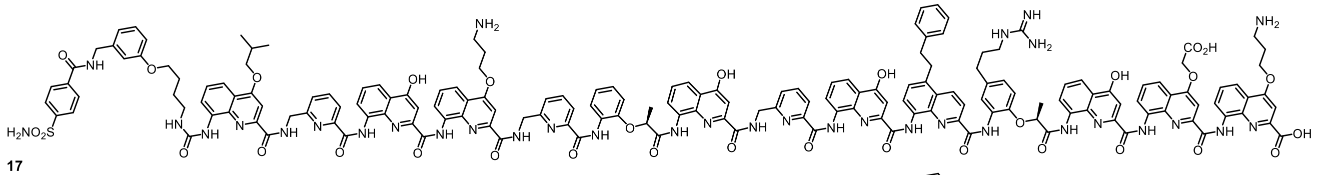

17

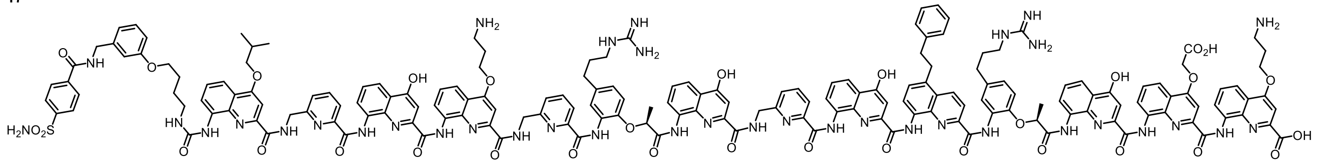

18

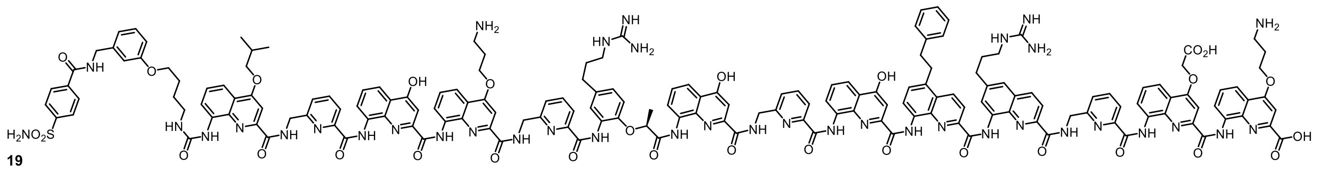

19

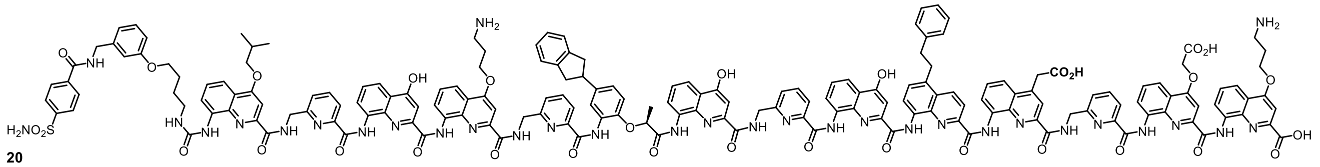

20

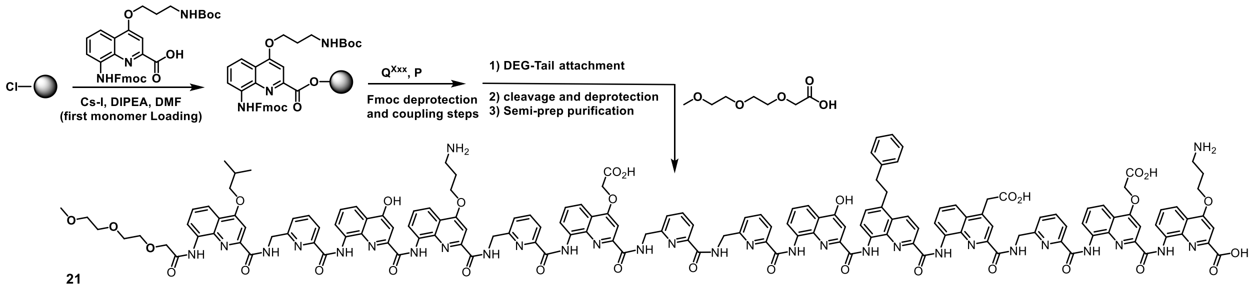

Scheme 1. Overview of the SPFS and chemical structures of all the synthesized AOF sequences (The final DEG tail group was installed via same coupling method of Q, B and P monomers).

5.3 AOF monomer and biotinylated HCAII ligand synthesis

Compound 25: (+)-Ethyl-D-lactate (1.1ml, 8.8 mmol, 1.0 equiv., enantiomer purity over 99%), triphenylphosphine (2.87 g, 10.56 mmol, 1.2 eq) and 4-bromo-2-nitrophenol (1,83 g, 8.8 mmol, 1.0 equiv.) were dissolved in THF (80 mL) under N_2 atmosphere. The mixture was cooled down to 0°C then DIAD (2.1 mL, 10.6 mmol, 1.2 equiv.) was added to the mixture slowly under the N_2 atmosphere. The reaction mixture was stirred at RT for three hours. The solvent was removed under vacuum and the crude was purified by silica gel column chromatography (CyHex/EtOAc, 9:1, v/v) to yield pale yellow crystalline powder (2.4 g, 7.92 mmol, 91%)

^{1}H-NMR (500MHz, $CDCl_3$): δ = 7.96 (d, J_1 = 4 Hz 1H), 7.58 (dd, J_1 = 8Hz, J_2 = 4Hz, 1H), 6.86 (d, *J* = 8 Hz, 1H), 4.80 (q, 1H), 4.21 (m, 2H), 1,68 (d, 2H), 1.25 (t, 3H). **^{13}C-NMR** (126 MHz, DMSO-*d*$_6$) δ 170.7, 150.3, 141.2, 136.6, 128.5, 117.6, 113.3, 74.9, 61.9, 18.4, 14.2. **HRMS** (ESI-) calcd. for $C_{11}H_{13}BrNO_5$ $[M\text{-}H]^-$ 316.9898, found: 316.9881.

Compound 26: Compound **25** (2.4 g, 7.92 mmol, 1.0 equiv.), *N*-Boc-propargylamine (1.84 g, 11.8 mmol, 1.5 equiv.) and TEA (2.76 mL, 2.5 equiv.) were dissolved in anhydrous THF (32 mL), and the solution was degassed 3 times by freeze pumping. Then CuI (114 mg, 396 µmol, 5 mol%) and $Pd(PPh_3)_4$ (396 µmol, 5 mol%) were added under Ar atmosphere and the reaction mixture was stirred at 60 °C for 18 hours. After reaction mixture dilution with H_2O, the mixture was extracted with DCM (3 ×), dried over $MgSO_4$ and solvents were removed under reduced pressure. The crude was purified further by silica gel column chromatography (CyHex/EtOAc 8:2 → 6:4, v/v), yielding **26** (2.5 g, 6.4 mmol, 80%) as a yellow oil.

^{1}H-NMR (500 MHz, $CDCl_3$) δ 7.87 (d, J = 2.1 Hz, 1H), 7.49 (dd, J = 8.7, 2.2 Hz, 1H), 6.87 (d, J = 8.7 Hz, 1H), 4.83 (q, J = 6.8 Hz, 1H), 4.76 (s, 1H), 4.21 (qq, J = 7.4, 3.7 Hz, 2H), 4.13 (p, J = 4.9,

4.1 Hz, 2H), 1.68 (d, J = 6.8 Hz, 3H), 1.60 (s, 2H), 1.47 (s, 9H), 1.23 (t, J = 7.1 Hz, 3H). **^{13}C NMR** (101 MHz, $CDCl_3$) δ 170.6, 155.3, 150.7, 140.3, 136.7, 128.8, 116.4, 115.6, 86.7, 80.3, 74.6, 61.8, 28.4, 18.3, 14.0. **HRMS** (ESI+) calcd. for $C_{19}H_{24}N_2O_7Na$ $[M+Na]^+$ 415.1481, found 415.1474

Compound 28: Compound **26** (2.4 g, 6.1 mmol, 1.0 equiv.) was suspended in DCM (5 mL) and TFA (5 mL) was added to the suspension. The solution was stirred for 60 min. Then the solvent was removed under reduced pressure and traces of TFA were finally removed by lyophilization. Compound **27** was directly used for the following process without further purification. The obtained TFA salts was dissolved in THF (82 mL) and DIPEA (4.25 mL, 24.4 mmol, 4 equiv.) was added to the mixture and the solution was cooled down to 0 °C. N,N'-Di-Boc-1H-pyrazole-1-carboxamidine (2.8 g, 9.1 mmol, 1.5 equiv.) was added and the resulting mixture was stirred overnight. The solvents were removed under reduced pressure, and the crude was dissolved in EtOAc and the organic phase was successively washed with 10% citric acid, saturated $NaHCO_3$, saturated NaCl and dried over Na_2SO_4. The crude was purified by silica gel column chromatography (CyHex/EtOAc 5:1, v/v) yielding **28** (2.0 g, 3.74 mmol, 60%) as a green solid.

^{1}H-NMR (500 MHz, $CDCl_3$): δ = 11.5 (s, 1H), 8.53 (t, 1H), 7.89 (d, J_1 = 4 Hz, 1H), 7.51 (dd, J_1 = 8.0Hz, J_2 =4.0Hz, 1H), 6.87 (d, J_1 = 8.0Hz, 1H), 4.84 (q, 1H), 4.45 (d, 2H), 4.21 (q, 2H), 1.68 (d, 3H), 1.51 (s, ,9H), 1.50 (s, ,9H), 1.23 (t, 3H) . **^{13}C NMR** (101 MHz, $CDCl_3$) δ 170.8, 151.3, 139.9, 137.3, 129.1, 115.7, 114.9, 84.5, 80.8, 74.4, 62.1, 30.3, 18.2, 14.1. **HRMS** (ESI+) calcd. for $C_{25}H_{35}N_4O_9$ $[M+H]^+$ 535.2404, found 535.2398.

Compound 29: Compound **28** (2.0 g, 3.74 mmol, 1.0 equiv.) was dissolved in THF (56 mL). After addition of a solution of LiOH (180 mg, 7.5 mmol, 2 equiv.) in H_2O (20 mL), the reaction mixture was stirred for 30 min at RT. Then, the mixture was acidified to approximately pH = 2 using 1 M HCl in H_2O. The resulting aqueous phase was extracted with DCM (3 ×) and dried over $MgSO_4$. After removing the solvents under reduced pressure, compound **29** was recovered quantitatively.

^{1}H-NMR (400 MHz, $CDCl_3$) δ 11.45 (s, 1H), 8.59 (s, 1H), 7.91 (d, J = 2.1 Hz, 1H), 7.54 (dd, J = 8.7, 2.1 Hz, 1H), 6.95 (d, J = 8.8 Hz, 1H), 5.06 (s, 3H), 4.88 (q, J = 6.8 Hz, 1H), 4.35 (s, 2H), 1.72 (d, J = 6.8 Hz, 3H), 1.50 (d, J = 2.6 Hz, 18H)**. ^{13}C-NMR**: (101 MHz, $CDCl_3$) δ 172.8, 162.8, 155.7, 153.0, 150.7, 139.8, 137.1, 129.2, 115.3, 85.2, 83.8, 81.3, 80.1, 74.5, 68.0, 31.5, 28.2, 28.0, 25.6, 18.2. **HRMS** (ESI+) calcd. for $C_{23}H_{31}N_4O_9$ $[M+H]^+$ 507.2091, found 507.2085.

Compound 30: Compound **29** (2.0 g, 3.74 mmol, 1.0 equiv.) and Na_2CO_3 (480 mg, 4.53 mmol, 1.2 equiv.) were dissolved in MeOH (56 mL). The solution was purged with N_2 for three times before adding Pd/C (200 mg, 10% w/w) and the N_2 was replaced by H_2 atmosphere. The reaction

mixture was stirred at room temperature for 17 h, filtered over celite and washed with MeOH. Solvents were evaporated under reduced pressure yielding compound **30** (1.8 g, 3.74 mmol) quantitatively.

^{1}H-NMR (400 MHz, DMSO-*d*$_6$) δ 11.48 (s, 1H), 8.29 (s, 1H), 6.57 (d, J = 8.0 Hz, 1H), 6.42 (d, J = 2.1 Hz, 1H), 6.24 (dd, J = 8.1, 2.1 Hz, 1H), 4.89 (s, 2H), 4.04 (q, J = 6.8 Hz, 1H), 3.24 (m, 2H), 2.38 (t, J = 7.7 Hz, 2H), 1.72 (m, 2H), 1.47 (s, 9H), 1.38 (s, 9H). 1.35 (d, 3H) **^{13}C-NMR** (101 MHz, DMSO-*d*$_6$) δ 175.8, 145.5, 139.7, 134.0, 116.0, 115.8, 114.5, 78.5, 49.1, 32.5, 30.8, 28.5, 28.1, 20.2. **HRMS** (ESI+) calcd. for $C_{23}H_{37}N_4O_7$ $[M+H]^+$ 481.2662, found 481.2657.

Compound 31: Compound **30** (1.8 g, 3.74 mmol, 1.0 equiv.) and $NaHCO_3$ (1.8 g, 18.7 mmol, 5.0 equiv.) were dissolved in H_2O (83 mL). Then, Fmoc-Cl (1.5 g, 4.8 mmol, 1.3 equiv.) dissolved in dioxane (83 mL) was added at 0 °C over 1 h. The reaction mixture was stirred at 0 °C for one additional hour and then at RT for 18 h. After the reaction mixture was acidified to approximately pH = 2 using 1 M HCl in H_2O, the aqueous phase was extracted with DCM (3×), dried over $MgSO_4$ and the solvents were removed under reduced pressure. The residue was purified by puriFlash®xs 520Plus purification system (line A: water, line B: ACN; 30% - 100% B 15min then 100%B 10min) to yield compound **31** (1.5 g, 2.13 mmol, 57%) as a white solid.

^{1}H-NMR (400 MHz, DMSO-*d*$_6$) δ 13.15 (s, 1H), 11.49 (s, 1H), 8.69 (s, 1H), 8.32 (t, J = 5.7 Hz, 1H), 7.92 (d, J = 7.5 Hz, 2H), 7.74 (dd, J = 7.3, 3.4 Hz, 2H), 7.51 (s, 1H), 7.43 (t, J = 7.4 Hz, 2H), 7.35 (td, J = 7.4, 1.2 Hz, 2H), 6.89 (d, J = 1.2 Hz, 2H), 4.76 (q, J = 6.8 Hz, 1H), 4.42 (dd, J = 7.3, 3.8 Hz, 2H), 4.32 (t, J = 7.0 Hz, 1H), 3.29 (m, 4H), 1.76 (p, J = 7.5 Hz, 2H), 1.56 (d, J = 6.8 Hz, 3H), 1.43 (d, 18H). **^{13}C-NMR** (101 MHz, DMSO-*d*$_6$) δ 174.1, 163.6, 155.7, 154.0, 152.5, 144.2, 141.2, 135.2, 128.2, 127.6, 125.7, 124.1, 120.6, 83.3, 78.6, 66.6, 47.0, 40.7, 40.5, 32.3, 30.7, 28.5, 28.1, 19.0. **HRMS** (ESI+) calcd. for $C_{38}H_{47}N_4O_9$ $[M+H]^+$703.3343, found 703.3339

Compound 32: The protocol was based on reported literature[25] and slightly modified. To a 250 mL two-neck flask flushed with a positive pressure of N_2, $Pd(OAc)_2$ (55 mg, 0.25 mmol, 5% mol), (1*H*-inden-2-yl)boronic acid (800 mg, 5 mmol, 1 equiv.), K_2CO_3 (2.8 g, 20 mmol, 4 equiv.) were added and the flask was again flushed with N_2. Compound **29** (1.58 g, 5 mmol, 1 equiv.) was dissolved in a mixture of toluene/ ethanol/ H_2O (37.5 mL: 15 mL: 7.5 mL, v/v/v) and degassed three times. The degassed solution was transferred to the two-neck flask and the mixture was heated to 80 °C overnight. The reaction mixture was diluted with water and extracted with DCM (3×) and dried over Na_2SO_4. The crude was purified first by silica gel chromatography and then

with a puriFlash®xs 520Plus purification system (line A: water, line B: ACN; 30% - 100% B 15min then 100%B 10min) to yield compound **32** (1.1 g, 3.15 mmol, 63%).

^{1}H-NMR (500 MHz, $CDCl_3$) δ 8.05 (d, J = 2.3 Hz, 1H), 7.72 (dd, J = 8.7, 2.3 Hz, 1H), 7.48 (dq, J = 7.3, 0.9 Hz, 1H), 7.41 (dt, J = 7.5, 0.9 Hz, 1H), 7.29 (td, J = 7.5, 1.2 Hz, 1H), 7.23 – 7.19 (m, 2H), 6.98 (d, J = 8.7 Hz, 1H), 4.86 (q, J = 6.8 Hz, 1H), 4.23 (qd, J = 7.1, 1.9 Hz, 2H), 3.76 (dd, J = 1.6, 0.8 Hz, 2H), 1.71 (d, J = 6.7 Hz, 3H), 1.26 (t, J = 7.1 Hz, 3H). **^{13}C-NMR** (126 MHz, $CDCl_3$) δ 171.0, 150.2, 144.9, 143.5, 143.0, 141.0, 130.6, 130.4, 127.8, 127.0, 125.5, 123.9, 122.6, 121.5, 116.4, 75.0, 61.8, 39.1, 18.5, 14.2. **HRMS** (ESI+) calcd. for $C_{20}H_{20}NO_5$ $[M+H]^+$ 354.1342, found 354.1291

Compound 33: Compound **32** (1.1 g, 3.1 mmol, 1 equiv.) was dissolved in THF (45 mL) and a LiOH (144 mg, 6.2 mmol, 2 equiv.) solution in water (15 mL) was added. After 1 h, the reaction mixture was acidified with 5% citric acid in water and the aqueous phase was extracted with DCM (3×). After drying with Na_2SO_4, the solvent was evaporated to yield compound **33** without further purification (0.9 g, 2.8 mmol, 90%).

^{1}H NMR (500 MHz, DMSO-*d*$_6$) δ 8.11 (d, *J* = 2.3 Hz, 1H), 7.91 (dd, *J* = 8.8, 2.3 Hz, 1H), 7.47 (d, *J* = 7.4 Hz, 1H), 7.40 (d, *J* = 7.7 Hz, 2H), 7.26 (td, *J* = 7.5, 1.2 Hz, 1H), 7.21 – 7.14 (m, 2H), 4.97 (d, *J* = 7.5 Hz, 1H), 3.84 (s, 2H), 1.50 (d, *J* = 6.8 Hz, 3H), **^{13}C NMR** (126 MHz, DMSO-d$_6$) δ 172.7, 150.1, 145.2, 144.5, 143.5, 140.6, 131.3, 129.0, 127.4, 127.0, 125.3, 124.2, 122.0, 121.5, 116.4, 74.1, 39.1, 18.6. **HRMS** (ESI+) calcd. for $C_{18}H_{15}NO_5$ $[M+Na]^+$ 348.0842, found 348.0896

Compound 34: Compound **33** (900 mg, 2.8 mmol, 1 equiv.) and Na_2CO_3 (300 mg, 1.0 equiv.) were dissolved in MeOH (40 mL), and the solution was purged three times with N_2 positive pressure. Pd/C (90 mg, 10%, w/w) was added and the N_2 was replaced by H_2 atmosphere. The reaction mixture was stirred at RT for 17 h, filtered over a celite pad and washed with MeOH. Solvents were evaporated under reduced pressure to furnish compound **34** (0.8 g) in quantitative yield without further purification.

Compound 35: Compound **34** (0.8 g 2.8 mmol, 1.0 equiv.) and $NaHCO_3$ (1.3 g, 14 mmol, 5.0 equiv.) were dissolved in H_2O (62 mL). Then, Fmoc-Cl (1.04 g, 3.64 mmol, 1.3 equiv.) in dioxane (62 ml) was added at 0 °C over 1 h. The reaction mixture was stirred at 0 °C for one additional hour and then at RT for 18 h. The reaction mixture was next acidified to approximately pH = 2 using 1 M HCl in H_2O, the aqueous phase was extracted with DCM (3x), dried over $MgSO_4$ and solvents were removed under reduced pressure. The residue was purified by puriFlash®xs

520Plus purification system (line A: water, line B: ACN; 50% - 100% B 15min then 100% B 10min) to yield compound **30** (0.65 g, 2.13 mmol, 45%) a white solid.

^{1}H-NMR (400 MHz, DMSO-*d*$_6$) δ 13.15 (s, 1H), 8.68 (s, 1H), 7.90 (d, J = 7.5 Hz, 2H), 7.73 (d, J = 7.0 Hz, 2H), 7.66 (s, 1H), 7.42 (t, J = 7.4 Hz, 2H), 7.38 – 7.27 (t, J = 7.5 Hz, 2H), 7.23 (dd, J = 5.4, 3.3 Hz, 2H), 7.19 – 7.07 (m, 2H), 6.99 (d, J = 8.5, 1H), 6.90 (d, J = 8.5 Hz, 1H), 4.79 (q, J = 6.8 Hz, 1H), 4.47 – 4.37 (m, 2H), 4.32 (q, J = 8.6, 7.1 Hz, 1H), 3.55 (p, J = 8.6 Hz, 1H), 3.24 (dd, J = 15.5, 8.1 Hz, 2H), 2.91 (dd, J = 15.5, 9.1 Hz, 2H), 1.56 (d, J = 6.8 Hz, 3H). **^{13}C-NMR** (101 MHz, DMSO-*d*$_6$) δ 207.0, 174.1, 154.0, 144.2, 143.0, 141.2, 138.8, 128.2, 127.6, 126.8, 125.8, 124.6, 122.8, 120.6, 115.0, 74.4, 66.6, 47.0, 44.9, 40.8, 31.2, 18.9. **HRMS** (ESI+) calcd. for $C_{33}H_{29}NO_5$ $[M+H]^+$ 519.2048, found 519.2008

Compound 37: Compound **36**[21] (2.1 g, 5.42 mmol, 1 equiv.) was dissolved in DMF (30 mL) and TEA (30 mL) was added. The reaction mixture was degassed by using freeze-thaw techniques. Then $Pd(PPh_3)Cl_2$ (76 mg, 0.11 mmol, 0.02 equiv.) and CuI (41 mg, 0.22 mmol, 0.04 equiv.) were added and the reaction mixture was again degassed twice using freeze-thaw techniques. Phenylacetylene (0.89 mL, 8.14 mmol, 1.5 equiv.) was added and the reaction mixture was heated up to 80 °C for 4 h. The reaction mixture was poured into a mixture of water and DCM, layers were then separated. Aqueous layer was extracted one more time with DCM. Combined organic layers were washed with water, brine (2 times), dried over $MgSO_4$, then the solvent was removed under reduced pressure. The crude was purified by silica gel chromatography in pure DCM to yield the target compound **37** (1.7 g, 4.17 mmol, 77%) as a yellow solid.

^{1}H-NMR (400 MHz, $CDCl_3$) δ 8.92 (d, J = 8.7 Hz, 1H), 8.38 (d, J = 8.7 Hz, 1H), 8.14 (d, J = 7.8 Hz, 1H), 7.92 (d, J = 7.8 Hz, 1H), 7.71 – 7.63 (m, 2H), 7.58 – 7.51 (m, 2H), 7.50 – 7.31 (m, 7H), 5.52 (s, 2H). **^{13}C-NMR** (101 MHz, $CDCl_3$) δ 164.5, 150.5, 147.8, 139.2, 136.4, 135.5, 132.1, 130.7, 130.2, 129.9, 128.8, 128.8, 128.5, 128.3, 126.2, 124.7, 123.2, 121.8, 99.2, 84.6, 67.9. **HRMS** (ESI+) calcd. for $C_{25}H_{16}N_2O_4$ $[M+H]^+$ 409.1188, found 409.1185

Compound 38: Pd/C (165 mg, 10% m/m) was added to compound **37** (1.65 g, 4.04 mmol, 1 equiv.) in EtOAc and DMF solvent mixture (18 mL/9 mL, 2:1) under N_2. N_2 was bubbled, followed by H_2 bubbling, then the H_2 balloon was placed and stirred at RT for 4 h. The reaction mixture was filtered through a celite pad, washing with EtOAc until the eluent was colorless. The solvent was removed to yield the target compound as brown solid without further purification.

^{1}**H-NMR** (400 MHz, DMSO-*d*$_6$) δ 8.55 (d, J = 8.8 Hz, 1H), 8.06 (d, J = 8.8 Hz, 1H), 7.29 – 7.22 (m, 5H), 7.21 – 7.13 (m, 1H), 6.81 (d, J = 7.8 Hz, 1H), 6.40 (s, 2H), 3.16 (dd, J = 9.5, 6.5 Hz, 2H), 2.89 (s, 2H). **HRMS** calculated for $C_{18}H_{15}N_2O_2$: 291.1134. $(M-H)^-$; Found :291.1137

Compound 39: 10% $NaHCO_3$ solution (71 mL) was added to the compound **38** in 1,4-dioxane (36 mL). Fmoc-Cl (1.36 g, 5.23 mmol, 1.3 equiv.) in 71 mL was added to the mixture dropwise in 0 °C. After adding, the solution was stirred in RT overnight. The reaction mixture was acidified using 5% citric acid, extracted with DCM (2 times). The combined organic layers were washed with water, brine (2 times), dried with Na_2SO_4. The solvent was evaporated, and crude was purified by silica gel chromatography with 5-10% MeOH in DCM mixture to yield 1.62 g target compound (4.07 mmol, 78%).

^{1}**H-NMR** (400 MHz, DMSO-*d*$_6$) δ 10.36 (s, 1H), 8.76 (d, J = 8.8 Hz, 1H), 8.20 (d, J = 8.8 Hz, 1H), 7.93 (d, J = 7.5 Hz, 2H), 7.77 (d, J = 7.4 Hz, 2H), 7.44 (td, J = 7.4, 1.2 Hz, 2H), 7.36 (td, J = 7.4, 1.2 Hz, 2H), 7.31 – 7.23 (m, 5H), 7.23 – 7.14 (m, 1H), 4.60 (d, J = 6.8 Hz, 2H), 4.44 (t, J = 6.8 Hz, 1H), 3.31 (t, J = 7.9 Hz, 4H), 2.94 (m, 2H). ^{13}**C-NMR** (126 MHz, DMSO-*d*$_6$) δ 165.6, 153.5, 145.8 – 144.8 (m), 143.7, 141.2, 140.8, 134.9, 134.0, 131.3, 128.9, 128.5, 128.2, 127.8, 127.6, 127.2, 125.9, 125.1, 120.4, 120.2, 115.8, 66.3, 46.6, 36.5, 32.9. **HRMS** (ESI-) calcd. for $C_{33}H_{25}N_2O_4$ $[M-H]^-$513.1814, found 513.1813

Compound 41: Compound **40** (1.62 g, 6.72 mmol, 1.3 equiv., prepared according to published protocol)[27], compound **36** (2.0 g, 5.17 mmol, 1 equiv.), DMF and Et_3N were mixed and degassed using freeze-thaw technic. $Pd(PPh_3)_2Cl_2$ (72.5 mg, 0.1 mmol, 0.02 equiv.) and CuI (39.4 mg, 0.21 mmol, 0.04 equiv.) were added and degassed twice further using freeze-thaw technic. The flask was carefully attached to the condenser under N_2 and the mixture was stirred at 80 °C for 3 to 4 hours. The reaction mixture was poured into a mixture of water and CH_2Cl_2. Aqueous phase was extracted by CH_2Cl_2. The combined organic phase was washed with water, brine (2x) then dried over MgSO4. After evaporation of CH2Cl2, the oily solid was triturated with EtOH, sonicated then put in fridge overnight. Sonicated again in the next morning, followed by a filtration and washed with cyclohexane. The target compound was obtained as a yellow solid (2.55 g, 90%).

^{1}**H NMR** (400 MHz, $CDCl_3$) δ 8.99 (d, *J* = 8.7 Hz, 1H), 8.41 (d, *J* = 8.7 Hz, 1H), 8.21 (d, *J* = 8.0 Hz, 1H), 8.16 (d, *J* = 8.0 Hz, 1H), 8.02 (s, 1H), 7.94 (d, *J* = 7.8 Hz, 1H), 7.80 (d, *J* = 7.8 Hz, 1H), 7.57-7.52 (m, 2H), 7.47-7.33 (m, 5H), 5.52 (s, 2H), 1.72 (s, 9H). ^{13}**C NMR** (100 MHz, $CDCl_3$) δ 164.4, 150.5, 149.0, 147.6, 139.2, 136.4, 135.5, 134.9, 130.5, 130.4, 130.1, 129.9, 128.8, 128.5,

128.3, 126.5, 125.8, 124.8, 123.8, 123.2, 120.0, 115.7, 102.1, 91.9, 88.3, 85.1, 67.9, 28.3. **HRMS** (ESI^+): *m/z* calcd for $C_{32}H_{26}N_3O_6$ $[M+H]^+$: 548.1822 found 548.1824.

Compound 42: Pd/C was added to the compound **41** (2.4 g, 4.38 mmol, 1 equiv.) dissolved in EtOAc (17 mL) /DMF(8.5 mL) solvent mixture under N_2. N_2 was bubbled, followed by H2 bubbling, then H2- ballon was replaced and stirred under RT or 4 h. The reaction mixture was filtered through a pad of celite, washing with EtOAc, solvent was then evaporated to yield target compound without further purification.

^{1}H NMR (400 MHz, DMSO-d_6) δ 13.08-12.64 (brs, 1H), 8.57 (d, *J* = 8.8 Hz, 1H), 8.07-8.02 (m, 2H,), 7.62 (d, *J* = 7.7 Hz, 1H), 7.45 (s, 1H), 7.37-7.29 (m, 2H), 7.26-7.21 (m, 1H), 6.84 (d, *J* = 7.7 Hz, 1H), 6.51-6.34 (brs, 2H), 3.30-3.22 (m, 2H), 3.01-2.93 (m, 2H, CH_2), 1.61 (s, 9H). **HRMS** (ESI^+): *m/z* calcd for $C_{25}H_{26}N_3O_4$ $[M+H]^+$: 432.1923 found 432.1912.

Compound 43: 10% $NaHCO_3$ solution (77 mL) was added to the compound **42** (1.89 g, 4.38 mmol, 1 equiv.) in 1,4-dioxane (39 mL). Fmoc-Cl (1.47 g, 5.69 mmol, 1.3 equiv.) in 77 mL was added to the mixture dropwise in 0 °C. After adding, the solution was stirred in RT overnight. The reaction mixture was acidified using 5% citric acid, extracted with DCM (2 times). The combined organic layers were washed with water, brine (2 times), dried with Na_2SO_4. The solvent was evaporated, and crude was purified by silica gel chromatography with 5-10% MeOH in DCM mixture to yield 1.4 g target compound (2.14 mmol, 49%).

^{1}H NMR (500 MHz, DMSO-d_6) δ 13.67-13.53 (brs, 1H), 10.47-10.35 (brs, 1H), 8.79 (d, *J* = 8.8 Hz, 1H), 8.39-8.17 (m, 2H), 8.04 (d, *J* = 8.1 Hz, 1H), 7.91 (d, *J* = 7.5 Hz, 2H), 7.78 (d, *J* = 7.5 Hz, 2H), 7.66-7.53 (m, 2H), 7.48-7.22 (m, 7H), 4.61 (d, *J* = 6.6 Hz, 2H), 4.45 (t, *J* = 6.6 Hz, 1H), 3.45-3.38 (m, 2H), 3.07-3.01 (m, 2H), 1.61 (s, 9H). **^{13}C NMR** (125 MHz, $CDCl_3$) δ 165.4, 153.5, 149.1, 145.0, 143.7, 140.8, 137.0, 135.1, 134.8, 134.1, 131.5, 130.1, 129.18, 127.8, 127.8, 127.2, 125.1, 124.3, 122.8, 122.5, 120.3, 120.2, 120.0, 119.3, 116.0, 114.7, 83.4, 66.3, 46.6, 30.7, 27.7, 25.7 ppm. **HRMS** (ESI^+): *m/z* calcd for $C_{40}H_{36}N_3O_6$ $[M+H]^+$: 654.2604 found 654.2598.

Compound 45: Compound **44** was synthesized according to the previous protocol.[26] Compound **44** (400 mg, 0.72 mmol, 1 equiv.) was dissolved in TFA/DCM mixture (20 mL, 1:1, v/v). After Boc-group removal, the sample was lyophilized without further purification. The resulting TFA salt was dissolved in a water / dioxane mixture (20 mL, 1:1, v/v) followed by the addition of DIPEA (500 μL, 2.88 mmol, 4 equiv.). The reaction mixture became cloudy. *tert*-Butyl (((tert-butoxycarbonyl)amino)(1H-pyrazol-1-yl)methylene)carbamate (336 mg, .0.8 mmol, 1.5 equiv.)

was then added and after 1 h the reaction mixture became clear. The reaction mixture was stirred overnight and after solvent evaporation the crude was purified by silica gel chromatography with 5-10% MeOH in DCM mixture to yield compound **45** (300 mg, 0.42 mmol, 58%).

^{1}H-NMR (500 MHz, DMSO-d_6) δ 13.53 (s, 1H), 11.45 (s, 1H), 10.47 (s, 1H), 8.47 (d, J = 8.6 Hz, 1H), 8.27 (t, J = 5.7 Hz, 1H), 8.17 (d, J = 8.5 Hz, 1H), 7.93 (d, J = 7.5 Hz, 2H), 7.77 (dd, J = 7.4, 1.1 Hz, 2H), 7.54 (d, J = 1.8 Hz, 1H), 7.44 (td, J = 7.5, 1.1 Hz, 2H), 7.36 (td, J = 7.4, 1.2 Hz, 2H), 4.60 (d, J = 6.9 Hz, 2H), 4.45 (t, J = 6.9 Hz, 1H), 3.37 – 3.36 (m, 2H), 2.78 (t, J = 7.5 Hz, 2H), 1.92 (p, J = 7.3 Hz, 2H), 1.41 (s, 9H), 1.36 (s, 9H). **^{13}C-NMR** (126 MHz, DMSO-d_6) δ 165.9, 155.7, 1574.0, 152.4, 145.1, 144.1, 143.7, 141.3, 138.4, 136.0, 129.8, 128.3, 127.7, 125.6, 121.2, 120.7, 119.8, 117.8, 83.2, 78.6, 66.9, 47.1, 33.7, 30.0, 28.4, 28.2, 28.1, 28.0. **HRMS** calcd. for $C_{39}H_{44}N_5O_8$: 710.3190. $(M+H)^+$ Found :710.3184

Compound 47: Compound **46** (2 g, 7.5 mmol, 1 equiv) was dissolved in a mixture of ethyl acetate and THF (92 mL, 1:1, v/v). TEA (41 mL) was added, and the reaction mixture was flushed with N_2. Pd/C (200 mg, 10% w/w) was next added, and the reaction mixture was stirred under H_2 pressure (1 bar) for 24 hours. The reaction mixture was filtered through a celite pad, washed with ethyl acetate and the solvent was removed under vacuum to yield compound **47** (1.42 g, 7 mmol, 94%) as orange oil.

^{1}H NMR (500 MHz, DMSO-d_6) δ 8.35 (d, *J* = 8.5 Hz, 1H), 8.03 (d, *J* = 8.5 Hz, 1H), 7.43 (t, *J* = 7.8 Hz, 1H), 7.13 (dd, *J* = 8.1, 1.3 Hz, 1H), 6.94 (dd, *J* = 7.6, 1.3 Hz, 1H), 6.08 (s, 2H), 3.95 (s, 3H). **^{13}C NMR** (126 MHz, DMSO-d_6) δ 165.3, 146.1, 143.9, 137.2, 136.4, 130.2, 129.7, 120.8, 113.3, 109.3, 52.5. **HRMS** calculated for $C_{11}H_{10}N_2O_2$: 203.0820 $(M+H)^+$ Found: 203.0766

Compound 48: Compound **47** (1.42 g, 7 mmol, 1 equiv.) was dissolved in 30 mL dioxane and mixed with LiOH (441 mg, 10.5 mmol, 1.5 equiv.) in 15 mL H_2O. The reaction was monitored by TLC. After reaction completion, 1 M aqueous HCl (10.7 mL) was added to quench the reaction. The mixture was then cooled down to 0 °C and $NaHCO_3$ (2.94 g, 35 mmol, 5 equiv.) was added. A solution of Fmoc-Cl (2.2 g, 8.4 mmol, 1.2 equiv.) in dioxane (93 mL) was added dropwise over 1 h. After reaction completion, 10% aqueous citric acid was added until pH = 4. The organic layer was separated by adding DCM and dried over $MgSO_4$. Solvent was removed under vacuum and the compound **48** was precipitated in MeOH to yield a yellow powder (2.17 g, 5.3 mmol, 76%)

^{1}H NMR (500 MHz, DMSO-d_6) δ 13.57 (s, 1H), 10.46 (s, 1H), 8.60 (d, *J* = 8.5 Hz, 1H), 8.35 (s, 1H), 8.21 (d, *J* = 8.5 Hz, 1H), 7.93 (d, *J* = 7.5 Hz, 2H), 7.78 (d, *J* = 7.5 Hz, 2H), 7.72 (dd, *J* = 8.2, 1.4 Hz, 1H), 7.67 (t, *J* = 7.9 Hz, 1H), 7.44 (t, *J* = 7.4 Hz, 2H), 7.36 (td, *J* = 7.4, 1.1 Hz, 2H), 4.63

(d, J = 6.8 Hz, 2H), 4.45 (t, J = 6.8 Hz, 1H). **^{13}C NMR** (126 MHz, DMSO-d_6) δ 165.7, 153.9, 145.7, 144.1, 141.2, 139.0, 136.9, 136.1, 129.9, 129.7, 128.2, 127.6, 125.5, 121.4, 121.0, 120.6, 116.6, 66.8, 47.0. **HRMS** (ESI+) calcd. for $C_{25}H_{18}N_2O_4$ $[M+H]^+$ 411.1344, found 411.1334.

Compound 24: Compound **22**[24] (12.0 mg, 32 µmol, 1.2 equiv.) was dissolved in a 5 mL round bottle flask with 500 µL DMF, followed by DIPEA (9.3 µL, 53 µmol, 2 equiv.). Biotin-PEG$_{12}$-NHS ester (25.0 mg,27 µmol, 1.0 equiv., purchased from Iris) was dissolved in 500 µL DMF and added to the reaction mixture. The reaction was monitored by HPLC and further purified by semi-prep HPLC to yield target compound as white powder (15.0 mg, 12.5 µmol, 47%)

^{1}H NMR (500 MHz, DMSO-d_6) δ 9.20 (t, J = 6.0 Hz, 1H), 8.07 – 8.01 (m, 2H), 7.94 – 7.87 (m, 2H), 7.84 (q, J = 5.7 Hz, 2H), 7.49 (s, 2H), 7.26 – 7.19 (m, 1H), 6.91 – 6.85 (m, 2H), 6.83 – 6.77 (m, 1H), 6.42 (s, 1H), 6.36 (s, 1H), 4.46 (d, J = 5.9 Hz, 2H), 4.33 – 4.27 (m, 1H), 4.12 (dd, J = 7.8, 4.4 Hz, 1H), 3.94 (t, J = 6.4 Hz, 2H), 3.58 (t, J = 6.5 Hz, 2H), 3.52 – 3.43 (m, 43H), 3.38 (t, J = 5.9 Hz, 2H), 3.18 (q, J = 5.9 Hz, 2H), 3.08 (qd, J = 6.6, 5.8, 3.6 Hz, 3H), 2.81 (dd, J = 12.4, 5.1 Hz, 1H), 2.57 (d, J = 12.4 Hz, 1H), 2.29 (t, J = 6.4 Hz, 2H), 2.06 (t, J = 7.8 Hz, 2H), 1.74 – 1.63 (m, 2H), 1.65 – 1.55 (m, 1H), 1.63 - 1.56(m, 1H), 1.55 – 1.39 (m, 2H), 1.36 – 1.21 (m, 2H). **HRMS** (ESI+) calcd. for $C_{55}H_{90}N_6O_{19}S_2$ $[M+2H]^{2+}$ 602.2924, found 602.2994

5.4 Foldamer synthesis on solid support

Compoun 2

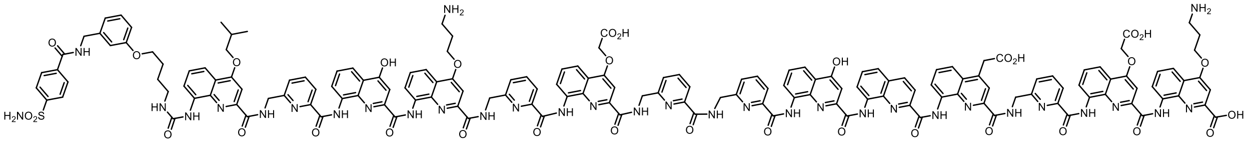

The scale was 22 μmol after first monomer loading determination. Target AOF was assembled on Cl-MPA protide resin using SPFS (**Method 5.1.1, 5.1.2**). 7 μmol of resin was used for ligand installation, after TFA cleavage, compound **2** was purified by semi-prep HPLC and recovered as yellow solid (4 mg, 1.3 μmol, 19%)

^{1}H NMR (300 MHz, DMSO-d_6) δ 12.10 (s, 1H), 11.73 (s, 1H), 11.23 (s, 1H), 10.99 (s, 1H), 10.93 (s, 1H), 10.74 (s, 1H), 10.63 (s, 1H), 10.22 (s, 1H), 9.97 (s, 1H), 9.12 (t, J = 6.1 Hz, 1H), 8.89 (s, 1H), 8.66 (s, 1H), 8.40 (s, 1H), 8.25 (dd, J = 24.6, 8.0 Hz, 3H), 8.04 – 7.95 (m, 2H), 7.95 (s, 1H), 7.88 (dd, J = 8.5, 6.7 Hz, 3H), 7.77 – 7.67 (m, 4H), 7.71 – 7.61 (m, 1H), 7.64 – 7.45 (m, 6H), 7.38 (s, 4H), 7.34 (d, J = 7.6 Hz, 1H), 7.26 – 7.15 (m, 2H), 7.12 (s, 8H), 7.13 – 6.96 (m, 4H), 6.96 – 6.57 (m, 7H), 6.53 (s, 1H), 6.44 (s, 1H), 6.34 (s, 1H), 6.21 (s, 1H), 5.39 – 5.32 (m, 1H), 4.82 (d, J = 16.0 Hz, 1H), 4.68 (d, J = 15.9 Hz, 1H), 4.45 – 4.33 (m, 7H), 4.21 (s, 1H), 3.97 (d, J = 16.2 Hz, 3H), 3.78 – 3.68 (m, 3H), 3.05 (s, 4H), 2.67 (s, 5H), 2.13 (s, 2H), 1.74 (d, J = 16.1 Hz, 1H), 1.42 (s, 3H), 1.26 (s, 6H), 1.13 – 1.03 (m, 6H). **HRMS** (ESI+) calcd. for $C_{160}H_{137}N_{33}O_{33}S$ $[M+2H]^{2+}$1539.9883, found 1540.1506

Compound 3

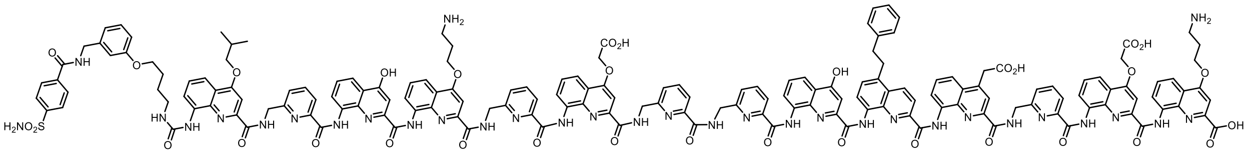

The scale was 15 μmol after first monomer loading determination. Target AOF was assembled on Cl-MPA protide resin using SPFS (**Method 5.1.1, 5.1.2**). 30 mg of crude was obtained after TFA cleavage, and the target compound was purified by semi-prep HPLC and recovered as a yellow solid (3.4 mg, 1.06 μmol, 7.1%).

^{1}H NMR (500 MHz, DMSO-d_6) δ 12.03 (s, 1H), 11.73 (s, 1H), 11.23 (s, 1H), 10.95 (s, 2H), 10.90 (s, 1H), 10.72 (s, 1H), 10.56 (s, 1H), 10.19 (s, 1H), 9.95 (s, 1H), 9.10 (t, *J* = 6.0 Hz, 1H), 8.89 (s, 1H), 8.66 (s, 1H), 8.49 (d, *J* = 8.7 Hz, 1H), 8.39 (s, 1H), 8.21 (dd, *J* = 17.2, 7.5 Hz, 2H), 7.98 (s,

1H), 7.98 (d, J = 8.5 Hz, 2H), 7.92 (s, 1H), 7.90 – 7.82 (m, 3H), 7.76 – 7.67 (m, 3H), 7.67 – 7.57 (m, 3H), 7.57 (s, 1H), 7.52 (m, 2H), 7.46 (m, 4H), 7.42 – 7.29 (m, 3H), 7.22 (m, 1H), 7.17 – 7.04 (m, 5H), 7.03 – 6.96 (m, 2H), 6.83 (d, J = 7.6 Hz, 1H), 6.78 (d, J = 7.6 Hz, 1H), 6.70 (dd, J = 19.5, 5.3 Hz, 3H), 6.60 (dd, J = 8.2, 2.5 Hz, 1H), 6.53 (s, 1H), 6.41 (s, 1H), 6.31 (s, 1H), 6.25 (s, 1H), 4.78 (d, J = 15.9 Hz, 1H), 4.65 (d, J = 16.0 Hz, 1H), 4.36 (d, J = 5.9 Hz, 2H), 4.32 (s, 4H), 4.19 (s, 1H), 3.96 (s, 3H), 3.87 (s, 1H), 3.72 (t, J = 6.5 Hz, 2H), 3.22 (s, 12H), 3.08 (t, J = 7.3 Hz, 1H), 3.03 (s, 5H), 3.01 (d, J = 5.5 Hz, 1H), 2.54 (s, 34H), 2.46 (s, 2H), 1.88 (s, 1H), 1.73 (d, J = 15.3 Hz, 1H), 1.63 (q, J = 5.8 Hz, 1H), 1.42 (dq, J = 12.8, 6.7, 5.3 Hz, 2H), 1.24 (d, J = 9.3 Hz, 8H), 1.17 (t, J = 7.3 Hz, 3H), 1.04 (dd, J = 6.7, 2.3 Hz, 5H), 0.96 (s, 3H), 0.85 (t, J = 6.7 Hz, 1H). **HRMS** (ESI+) calcd. for $C_{168}H_{143}N_{33}O_{33}S$ $[M+2H]^{2+}$ 1593.0225, found 1592.9842

Compound 15

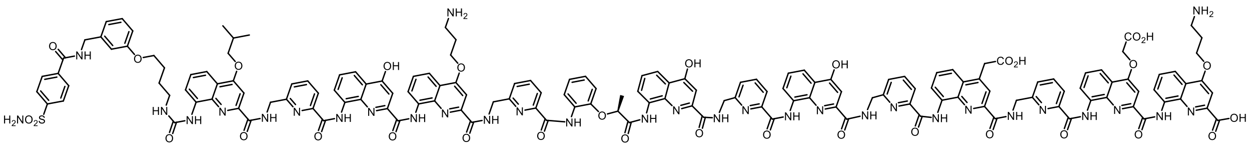

The scale was 13 µmol after first monomer loading determination. Target AOF was assembled on Cl-MPA protide resin using SPFS (**Method 5.1.1, 5.1.2**). 25 mg crude was obtained after TFA cleavage, and target compound was purified by semi-prep HPLC and recovered as a yellow solid (1.1 mg, 0.37 µmol, 2.8%).

^{1}H NMR (500 MHz, DMSO-d_6) δ 13.32 (s, 1H), 12.66 (s, 1H), 12.16 (s, 1H), 11.67 (s, 1H), 11.48 (s, 1H), 11.35 (s, 1H), 11.26 (s, 1H), 11.12 (s, 1H), 10.97 (s, 1H), 10.80 (s, 1H), 10.48 (s, 1H), 10.33 (s, 1H), 9.05 (t, J = 6.0 Hz, 1H), 8.93 (s, 1H), 8.71 (s, 1H), 8.18 (d, J = 7.6 Hz, 1H), 8.13 (s, 1H), 7.91 (d, J = 8.4 Hz, 3H), 7.86 – 7.77 (m, 12H), 7.73 (d, J = 8.3 Hz, 3H), 7.68 (d, J = 9.1 Hz, 6H), 7.62 (d, J = 8.0 Hz, 1H), 7.56 (d, J = 7.7 Hz, 2H), 7.50 – 7.39 (m, 6H), 7.34 – 7.23 (m, 4H), 7.18 (s, 1H), 7.08 (d, J = 15.6 Hz, 4H), 7.02 (t, J = 7.8 Hz, 1H), 6.72 (d, J = 7.6 Hz, 1H), 6.67 (s, 1H), 6.56 (dd, J = 8.2, 2.5 Hz, 1H), 6.40 (s, 1H), 6.35 (s, 1H), 6.28 (s, 2H), 6.16 (s, 1H), 6.07 (s, 1H), 5.86 (s, 1H), 4.79 (d, J = 15.9 Hz, 1H), 4.68 (d, J = 16.4 Hz, 1H), 4.38 (s, 2H), 4.29 (d, J = 5.9 Hz, 2H), 4.15 (s, 1H), 3.97 (s, 2H), 3.92 (d, J = 14.9 Hz, 1H), 3.68 (t, J = 6.4 Hz, 3H), 3.44 (s, 1H), 3.07 (s, 6H), 2.99 – 2.94 (m, 3H), 2.75 (s, 1H), 2.70 (s, 2H), 2.18 (s, 3H), 2.15 – 2.04 (m, 1H), 2.05 (s, 2H), 1.97 – 1.89 (m, 1H), 1.39 (t, J = 7.5 Hz, 3H), 1.22 (d, J = 7.8 Hz, 3H), 1.18 (p, J = 5.7, 5.1 Hz, 4H), 1.11 (t, J = 7.3 Hz, 1H), 1.04 – 0.98 (m, 7H), 0.78 (t, J = 6.8 Hz, 1H), -0.27 (s, 3H). **HRMS** (ESI+) calcd. for $C_{157}H_{136}N_{32}O_{32}S$ $[M+2H]^{2+}$ 1507.4932, found 1507.5003

Compound 16

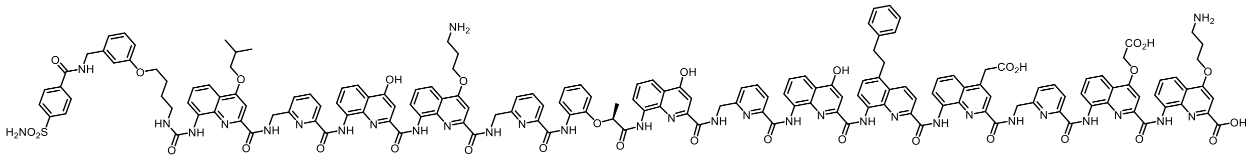

The scale was 15 µmol after first monomer loading determination. Target AOF was assembled on Cl-MPA protide resin using SPFS (**Method 5.1.1, 5.1.2**). 33 mg crude was obtained after TFA cleavage, Target compound was purified by semi-prep HPLC and recovered as yellow solid (3.7 mg, 1.17 µmol, 7.8%)

^{1}H NMR (500 MHz, DMSO-d_6) δ 11.46 (s, 1H), 11.36 (s, 1H), 11.20 (s, 1H), 11.14 (s, 1H), 10.92 (s, 1H), 10.87 (s, 1H), 10.69 (s, 1H), 10.61 (s, 1H), 10.44 (s, 1H), 9.12 (t, *J* = 6.3 Hz, 1H), 8.88 (s, 1H), 8.60 (s, 1H), 8.48 (d, *J* = 10.5 Hz, 1H), 8.18 (d, *J* = 9.0 Hz, 1H), 8.07 (d, *J* = 8.9 Hz, 2H), 7.97 – 7.90 (m, 5H), 7.84 (d, *J* = 8.3 Hz, 3H), 7.77 (s, 6H), 7.74 (d, *J* = 9.3 Hz, 1H), 7.73 – 7.66 (m, 2H), 7.60 (m, 3H), 7.49 (m, 2H), 7.47 (s, 5H), 7.43 (s, 2H), 7.37 (m, 3H), 7.30 (s, 1H), 7.21 (d, *J* = 7.0 Hz, 2H), 7.14 (dd, *J* = 15.9, 8.3 Hz, 3H), 7.07 (d, *J* = 8.9 Hz, 4H), 6.99 (d, *J* = 12.6 Hz, 3H), 6.88 (dd, *J* = 15.8, 8.8 Hz, 2H), 6.75 (d, *J* = 8.9 Hz, 3H), 6.68 (s, 2H), 6.59 (t, *J* = 11.6 Hz, 2H), 6.52 (d, *J* = 9.6 Hz, 2H), 6.43 (s, 1H), 6.33 (d, *J* = 6.9 Hz, 2H), 6.27 – 6.18 (m, 3H), 6.06 (t, *J* = 8.7 Hz, 1H), 5.72 – 5.66 (m, 1H), 4.84 (m, 1H), 4.69 (d, *J* = 17.0 Hz, 1H), 4.40 (s, 1H), 4.32 (d, *J* = 6.6 Hz, 1H), 1.37 (q, *J* = 10.5, 9.0 Hz, 3H), 1.21 (s, 6H), 1.16 (t, *J* = 7.2 Hz, 1H), 1.05 (d, *J* = 7.8 Hz, 8H), 0.81 (d, *J* = 8.9 Hz, 1H), -0.52 (d, 3H). **HRMS** (ESI+) calcd. for $C_{168}H_{144}N_{32}O_{32}S$ $[M+2H]^{2+}$1578.5274, found 1578.5383

Compound 17

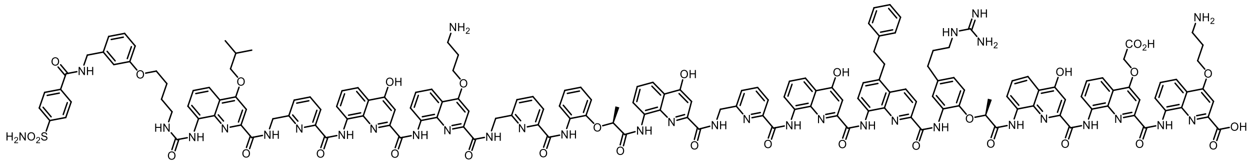

The scale was 15 µmol after first loading monomer determination. Target AOF was assembled on Cl-MPA protide resin using SPFS (**Method 5.1.1, 5.1.2**). 25 mg crude was obtained after TFA cleavage. AOF was purified by semi-prep HPLC and recovered as a yellow solid (1.36 mg, 0.42 µmol, 2.8%).

^{1}H NMR (500 MHz, DMSO-d_6) δ 11.55 (s, 1H), 11.44 (s, 1H), 11.26 (s, 2H), 10.90 (s, 1H), 10.64 (s, 1H), 10.26 (s, 1H), 9.66 (s, 1H), 9.35 (s, 1H), 9.10 (s, 2H), 9.01 (s, 3H), 8.80 (s, 1H), 8.67 (s,

1H), 8.26 (s, 3H), 7.98 (d, *J* = 8.2 Hz, 3H), 7.87 (d, *J* = 8.4 Hz, 4H), 7.82 (s, 3H), 7.58 – 7.48 (m, 6H), 7.45 (s, 5H), 7.34 (s, 5H), 7.19 (s, 6H), 7.08 (d, *J* = 7.3 Hz, 3H), 6.78 (d, *J* = 7.6 Hz, 3H), 6.62 (d, *J* = 11.6 Hz, 6H), 6.26 (s, 4H), 6.12 (s, 3H), 6.03 (s, 3H), 5.32 (t, *J* = 5.0 Hz, 4H), 4.49 (s, 3H), 4.36 (d, *J* = 6.1 Hz, 3H), 3.30 (s, 21H), 2.54 (s, 19H), 2.02 – 1.96 (m, 16H), 1.46 (d, *J* = 7.2 Hz, 10H), 1.37 (d, *J* = 16.3 Hz, 3H), 1.28 (d, *J* = 4.1 Hz, 3H), 1.17 (t, *J* = 7.4 Hz, 8H), 1.08 (d, *J* = 6.7 Hz, 9H), 0.85 (t, *J* = 6.8 Hz, 12H), -0.59 (s, 3H). **HRMS** (ESI+) calcd. for $C_{172}H_{154}N_{34}O_{32}S$ $[M+2H]^{2+}$ 1621.5645, found 1621.5794

Compound 18

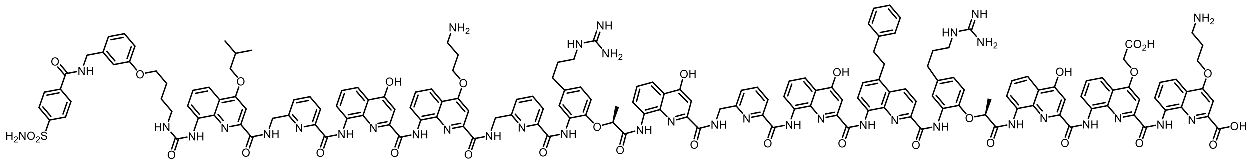

The scale was 12.5 µmol after first monomer loading determination. Target AOF was assembled on Cl-MPA protide resin using SPFS (**Method 5.1.1, 5.1.2**). 31 mg crude was obtained after TFA cleavage AOF was purified by semi-prep HPLC and recovered as yellow solid (1.43 mg, 0.43 µmol, 3.4%).

^{1}H NMR (500 MHz, DMSO-*d*$_6$) δ 11.75 (s, 1H), 11.64 (s, 1H), 11.48 (s, 1H), 11.42 (s, 1H), 11.36 (s, 1H), 11.29 (s, 1H), 11.20 (s, 1H), 10.91 (s, 1H), 10.80 (s, 1H), 10.21 (s, 1H), 9.26 (s, 1H), 9.06 (t, *J* = 5.9 Hz, 1H), 8.93 (s, 1H), 8.71 (s, 1H), 8.60 (s, 1H), 8.48 (d, *J* = 7.6 Hz, 1H), 8.31 (d, *J* = 7.6 Hz, 1H), 8.20 (m, 2H), 8.13 (d, *J* = 6.9 Hz, 1H), 8.07 (d, *J* = 8.5 Hz, 1H), 8.00 (s, 1H), 7.94 – 7.88 (m, 2H), 7.81 (d, *J* = 2.1 Hz, 2H), 7.82 – 7.70 (m, 3H), 7.67 (s, 1H), 7.62 – 7.53 (m, 2H), 7.45 (m, 13H), 7.29 (m, 6H), 7.16 (s, 1H), 7.14 – 6.96 (m, 5H), 6.91 (d, *J* = 7.5 Hz, 1H), 6.85 (d, *J* = 7.7 Hz, 2H), 6.82 – 6.69 (m, 6H), 6.67 (s, 1H), 6.56 (d, *J* = 8.4 Hz, 1H), 6.53 (d, *J* = 7.7 Hz, 2H), 6.49 (s, 1H), 6.37 (s, 1H), 6.27 (s, 2H), 6.10 (s, 1H), 6.05 (s, 2H), 5.94 (s, 1H), 5.85 (d, *J* = 8.2 Hz, 1H), 5.26 (t, *J* = 5.0 Hz, 1H), 5.07 (s, 4H), 4.41 (s, 3H), 4.29 (d, *J* = 5.9 Hz, 2H), 3.98 (s, 4H), 3.69 (s, 1H), 3.44 (s, 3H), 2.92 (s, 6H), 2.63 (s, 1H), 2.47 (s, 1H), 2.18 (s, 4H), 2.11 (dd, *J* = 13.9, 6.8 Hz, 1H), 2.06 (s, 3H), 2.01 (s, 1H), 1.97 (s, 5H), 1.96 – 1.87 (m, 2H), 1.51 (d, *J* = 7.5 Hz, 1H), 1.40 (s, 4H), 1.19 (d, *J* = 23.2 Hz, 12H), 1.11 (t, *J* = 7.3 Hz, 1H), 1.03 (dd, *J* = 6.6, 1.5 Hz, 7H), 0.82 – 0.75 (m, 2H), -0.58 (d, *J* = 5.9 Hz, 6H). **HRMS** (ESI+) calcd. for $C_{176}H_{163}N_{37}O_{32}S$ $[M+2H]^{2+}$1671.1094, found 1671.1189

Compound 19

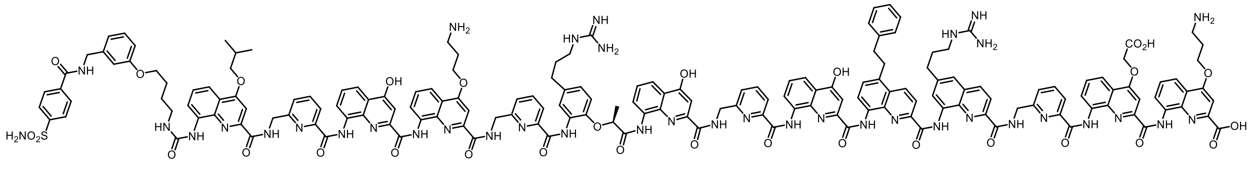

The scale was 12.5 µmol after first monomer loading determination. Target AOF was assembled on Cl-MPA protide resin using SPFS **Method 5.1.1, 5.1.2**). 36 mg crude was obtained after TFA cleavage. AOF was purified by semi-prep HPLC and recovered as yellow solid (3 mg, 0.91 µmol, 7.3%).

^{1}H NMR (500 MHz, Acetonitrile-d_3) δ 11.14 (s, 1H), 11.02 (s, 1H), 10.50 (s, 1H), 10.30 (s, 1H), 8.81 (s, 1H), 8.52 (s, 2H), 8.41 (s, 3H), 7.80 (dd, *J* = 15.8, 6.9 Hz, 10H), 7.68 (d, *J* = 13.0 Hz, 4H), 7.60 – 7.51 (m, 4H), 7.46 (s, 7H), 7.46 (t, *J* = 7.7 Hz, 4H), 7.42 – 7.31 (m, 4H), 7.14 – 6.91 (m, 8H), 6.86 – 6.75 (m, 4H), 6.65 (s, 2H), 6.58 (q, *J* = 9.3, 8.8 Hz, 4H), 5.97 (d, *J* = 15.9 Hz, 3H), 5.51 (d, *J* = 10.3 Hz, 1H), 5.22 (s, 1H), 3.58 (s, 2H), 3.09 (qd, *J* = 7.9, 5.3 Hz, 6H), 2.30 (s, 3H), 2.19 (s, 6H), 1.52 (s, 5H), 1.20 (q, *J* = 8.8, 7.9 Hz, 10H), 1.08 (d, *J* = 6.2 Hz, 4H), 1.05 (q, *J* = 6.5 Hz, 11H), -0.85 (s, 3H). **HRMS** (ESI+) calcd. for $C_{174}H_{160}N_{38}O_{30}S$ $[M+2H]^{2+}$ 1648.6043, found 1648.6123

Compound 20

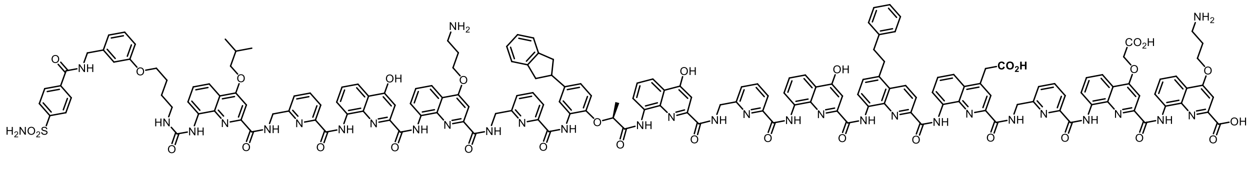

The scale was 13 µmol after first monomer loading determination. Target AOF was assembled on Cl-MPA protide resin using SPFS (**Method 5.1.1, 5.1.2**). 32 mg crude was obtained after TFA cleavage. AOF was purified by semi-prep HPLC and recovered as yellow solid (2.5 mg, 0.76 µmol, 6%).

^{1}H NMR (500 MHz, DMSO-d_6) δ 12.02 (s, 1H), 11.72 (s, 1H), 11.64 (s, 1H), 11.34 (s, 1H), 11.30 (s, 1H), 11.07 (s, 1H), 10.94 (d, *J* = 16.0 Hz, 2H), 10.73 (d, *J* = 16.2 Hz, 2H), 10.57 (s, 1H), 9.53 (s, 1H), 9.17 (t, *J* = 6.0 Hz, 1H), 9.06 (s, 1H), 8.87 (s, 1H), 8.55 (d, *J* = 8.7 Hz, 1H), 8.38 (d, *J* = 7.9 Hz, 1H), 8.30 (d, *J* = 7.3 Hz, 1H), 8.23 (d, *J* = 7.4 Hz, 1H), 8.04 – 7.98 (m, 3H), 7.92 (d, *J* = 13.8 Hz, 4H), 7.92 – 7.86 (m, 3H), 7.81 (s, 7H), 7.77 (d, *J* = 16.6 Hz, 1H), 7.73 (d, *J* = 8.9 Hz, 3H), 7.64 (ddd, *J* = 26.4, 14.3, 7.4 Hz, 6H), 7.55 (d, *J* = 8.0 Hz, 1H), 7.55 – 7.50 (m, 2H), 7.50 – 7.42

(m, 7H), 7.38 (s, 7H), 7.42 – 6.99 (m, 15H), 6.99 – 6.91 (m, 2H), 6.87 (d, *J* = 7.3 Hz, 1H), 6.83 (s, 1H), 6.80 (d, *J* = 14.4 Hz, 5H), 6.69 (dd, *J* = 25.8, 8.9 Hz, 2H), 6.60 (d, *J* = 7.7 Hz, 1H), 6.50 (s, 1H), 6.44 (s, 1H), 6.40 (d, *J* = 7.8 Hz, 1H), 6.36 (d, *J* = 7.0 Hz, 2H), 5.81 (s, 1H), 4.85 (d, *J* = 16.1 Hz, 1H), 4.73 (d, *J* = 15.9 Hz, 1H), 4.61 (s, 1H), 4.40 (d, *J* = 5.9 Hz, 3H), 4.32 (s, 1H), 4.17 (s, 1H), 4.01 (d, *J* = 15.0 Hz, 1H), 3.95 (s, 2H), 3.87 (d, *J* = 17.2 Hz, 3H), 3.50 (s, 1H), 3.24 (d, *J* = 14.9 Hz, 1H), 3.19 – 3.13 (m, 2H), 3.09 (dd, *J* = 7.3, 4.7 Hz, 1H), 3.01 (s, 2H), 2.95 (s, 2H), 2.86 – 2.78 (m, 2H), 2.69 (s, 1H), 2.21 – 2.07 (m, 2H), 2.07 (s, 5H), 2.04 – 1.95 (m, 1H), 1.60 (s, 2H), 1.44 (d, *J* = 7.4 Hz, 1H), 1.42 (s, 3H), 1.23 (s, 7H), 1.18 (t, *J* = 7.3 Hz, 1H), 1.06 (d, *J* = 6.6 Hz, 1H), 1.01 (d, *J* = 6.8 Hz, 6H), 0.88 – 0.82 (m, 1H), -0.20 (s, 3H). **HRMS** (ESI+) calcd. for $C_{177}H_{152}N_{32}O_{32}S$ $[M+2H]^{2+}$ 1636.558, found 1636.5659

Compound 21

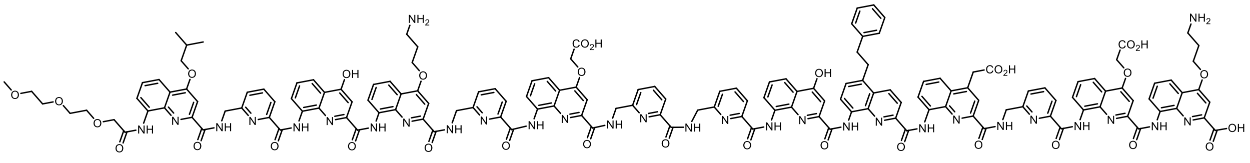

The scale was 15 μmol after first monomer loading determination. Target compound was prepared on Cl-protide resin using SPPS (**Method 5.1.1, 5.1.2**). 39 mg crude was obtained after TFA cleavage, 7 mg target compound was obtained purified by semi-prep HPLC as yellow solid (2.4 μmol, 16%)

1H NMR (500 MHz, DMSO-d6) δ 13.33 (s, 1H), 12.03 (s, 1H), 11.39 (s, 1H), 11.33 (s, 1H), 11.14 (s, 1H), 10.94 (s, 1H), 10.87 (s, 1H), 10.67 (s, 1H), 10.59 (s, 1H), 10.51 (s, 1H), 10.08 (s, 1H), 9.85 (s, 1H), 9.30 (s, 1H), 8.47 (d, J = 8.6 Hz, 1H), 8.37 (s, 1H), 8.27 – 8.19 (m, 2H), 7.95 – 7.87 (m, 3H), 7.89 – 7.82 (m, 4H), 7.79 (q, J = 6.7, 6.1 Hz, 1H), 7.72 (d, J = 11.7 Hz, 4H), 7.71 – 7.62 (m, 2H), 7.65 – 7.52 (m, 4H), 7.55 – 7.47 (m, 4H), 7.47 (t, J = 7.5 Hz, 2H), 7.41 (t, J = 7.3 Hz, 1H), 7.38 – 7.23 (m, 7H), 7.17 (dd, J = 14.8, 7.9 Hz, 5H), 7.10 (t, J = 6.9 Hz, 1H), 7.10 – 7.01 (m, 6H), 7.01 – 6.89 (m, 5H), 6.81 (q, J = 8.0, 7.2 Hz, 4H), 6.68 – 6.63 (m, 2H), 6.54 (dd, J = 18.0, 7.3 Hz, 2H), 6.40 (d, J = 12.2 Hz, 1H), 6.28 (s, 1H), 6.17 (s, 1H), 5.16 (s, 1H), 4.77 (d, J = 15.8 Hz, 1H), 4.65 (d, J = 15.8 Hz, 1H), 4.37 (d, J = 13.1 Hz, 2H), 4.31 (d, J = 7.5 Hz, 2H), 4.22 (d, J = 14.8 Hz, 1H), 3.98 (d, J = 7.9 Hz, 1H), 3.96 – 3.86 (m, 2H), 3.65 (d, J = 15.0 Hz, 1H), 3.50 (s, 2H), 3.19 – 3.13 (m, 2H), 3.05 – 2.99 (m, 1H), 2.80 – 2.63 (m, 4H), 2.70 (s, 3H), 2.22 (dq, J = 20.2, 6.9 Hz, 2H), 2.16 – 2.04 (m, 2H), 1.78 (d, J = 24.4 Hz, 1H), 1.69 (d, J = 15.5 Hz, 1H), 1.24 (d, J = 12.1 Hz, 1H), 1.23 (s, 2H), 1.17 (t, J = 7.3 Hz, 1H), 1.08 (t, J = 6.9 Hz, 6H). HRMS (ESI+) calcd. for $C_{156}H_{134}N_{30}O_{32}$ $[M+2H]^{2+}$1470.9948, found 1471.0129

5.5 NMR spectra and RP-HPLC chromatograms of new compounds

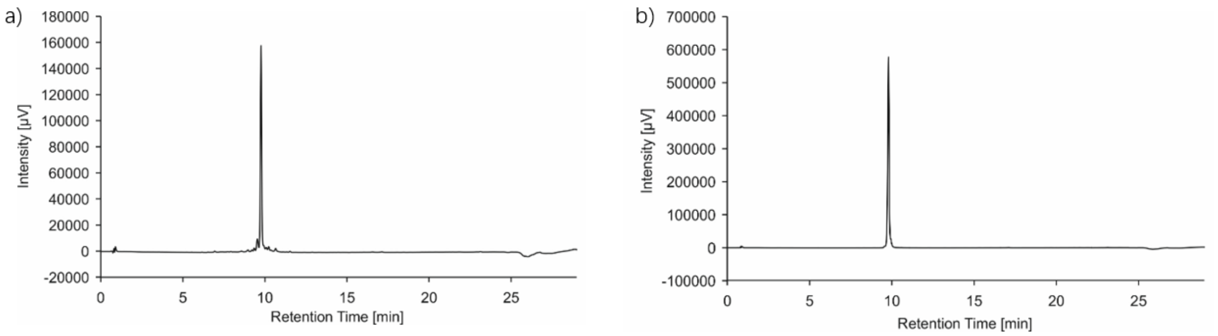

Figure S25. Analytical data of compound **2**. RP-HPLC chromatograms (a) after cleavage from the resin (C18, 5 to 100 B% over 23 min, 50 °C, λ = 254 nm) and (b) after purification (C18, 10 to 100 B% over 10 min, 50 °C, λ = 254 nm); A: 0.1% TFA water, B: 0.1% TFA acetonitrile

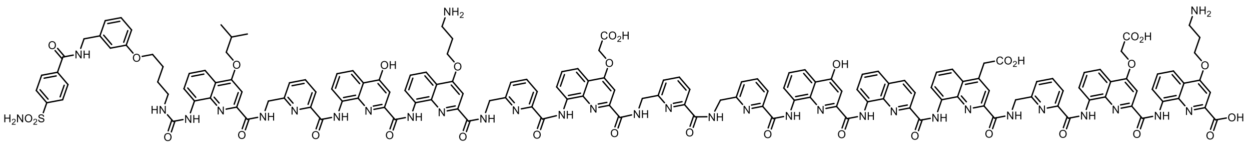

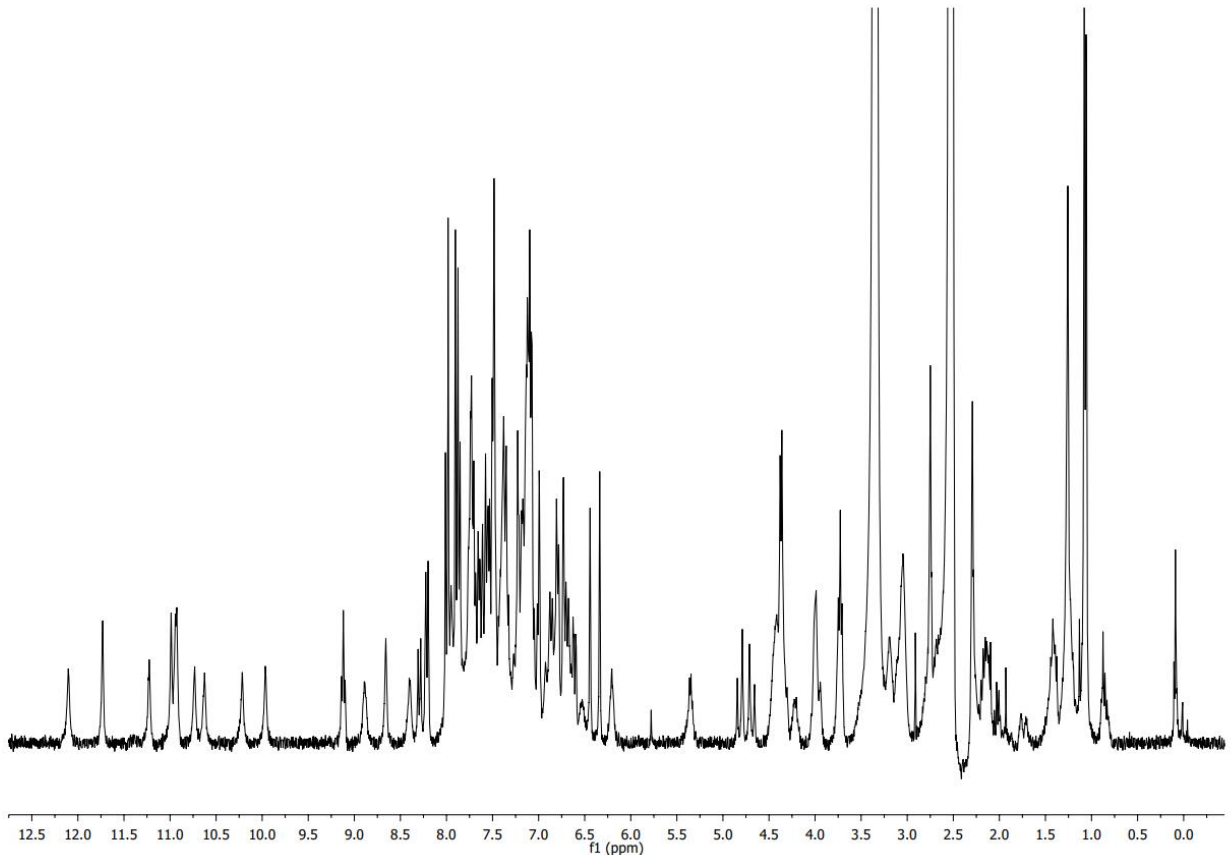

^{1}H NMR spectrum (300 MHz, DMSO-d_6, 25 °C)

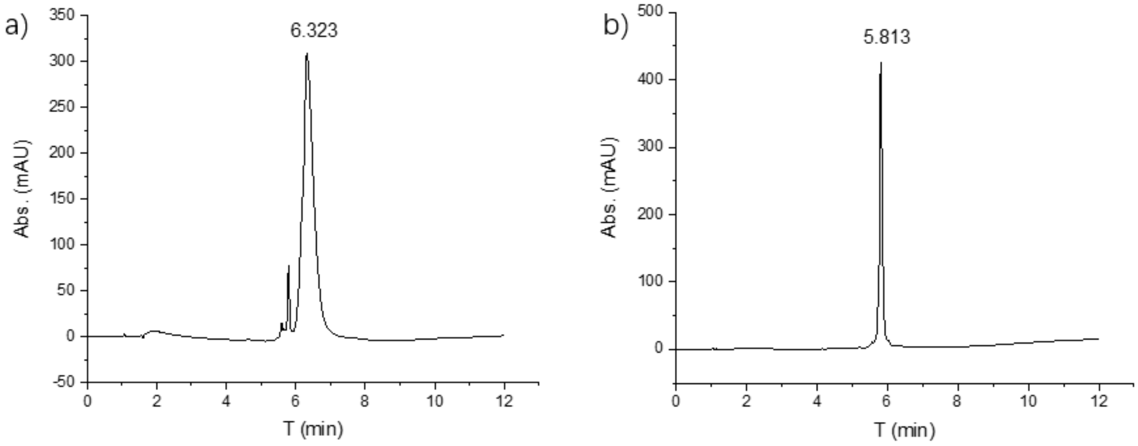

Figure S26. Analytical data of compound **3**. RP-HPLC chromatograms (a) after cleavage from the resin (C18, 10 to 100 B% over 10 min, 50 °C, λ = 300 nm) and (b) after purification (C18, 10 to 100 B% over 10 min, 50 °C, λ = 254 nm); A: 0.1% TFA water, B: 0.1% TFA acetonitrile

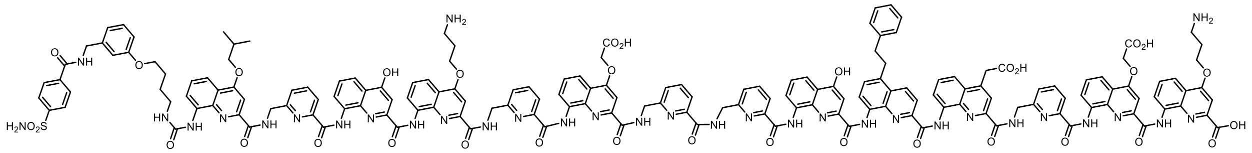

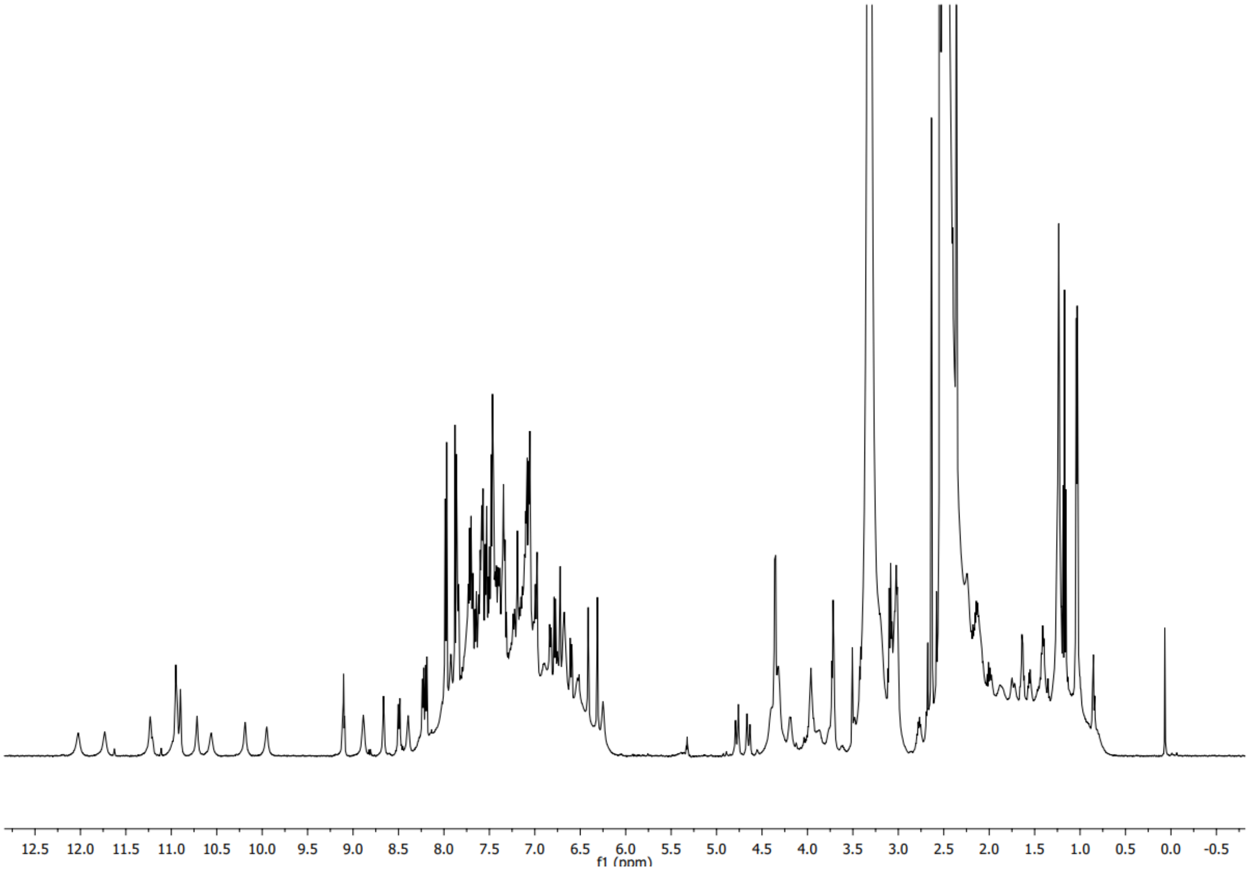

^{1}H NMR spectrum (500 MHz, DMSO-d_6, 25 °C)

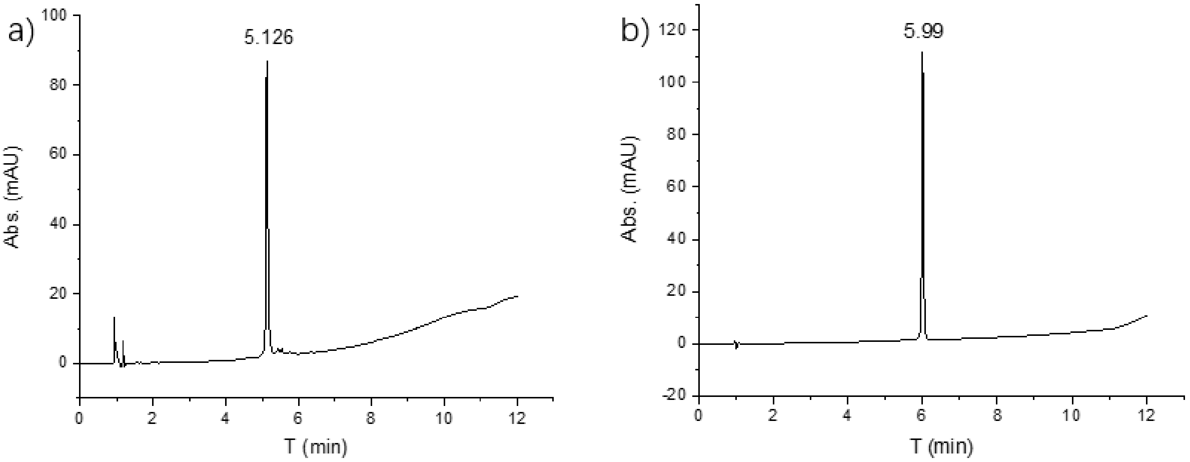

Figure S27. Analytical data of compound **15**. RP-HPLC chromatograms (a) after cleavage from the resin (C18, 10 to 100 B% over 10 min, 50 °C, λ = 254 nm) and (b) after purification (C18, 20 to 70 B% over 10 min, 50 °C, λ = 254 nm); A: 0.1% TFA water, B: 0.1% TFA acetonitrile.

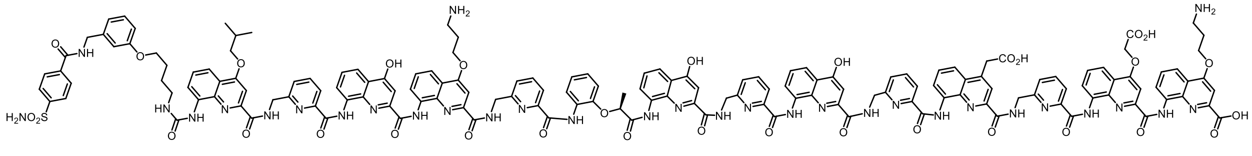

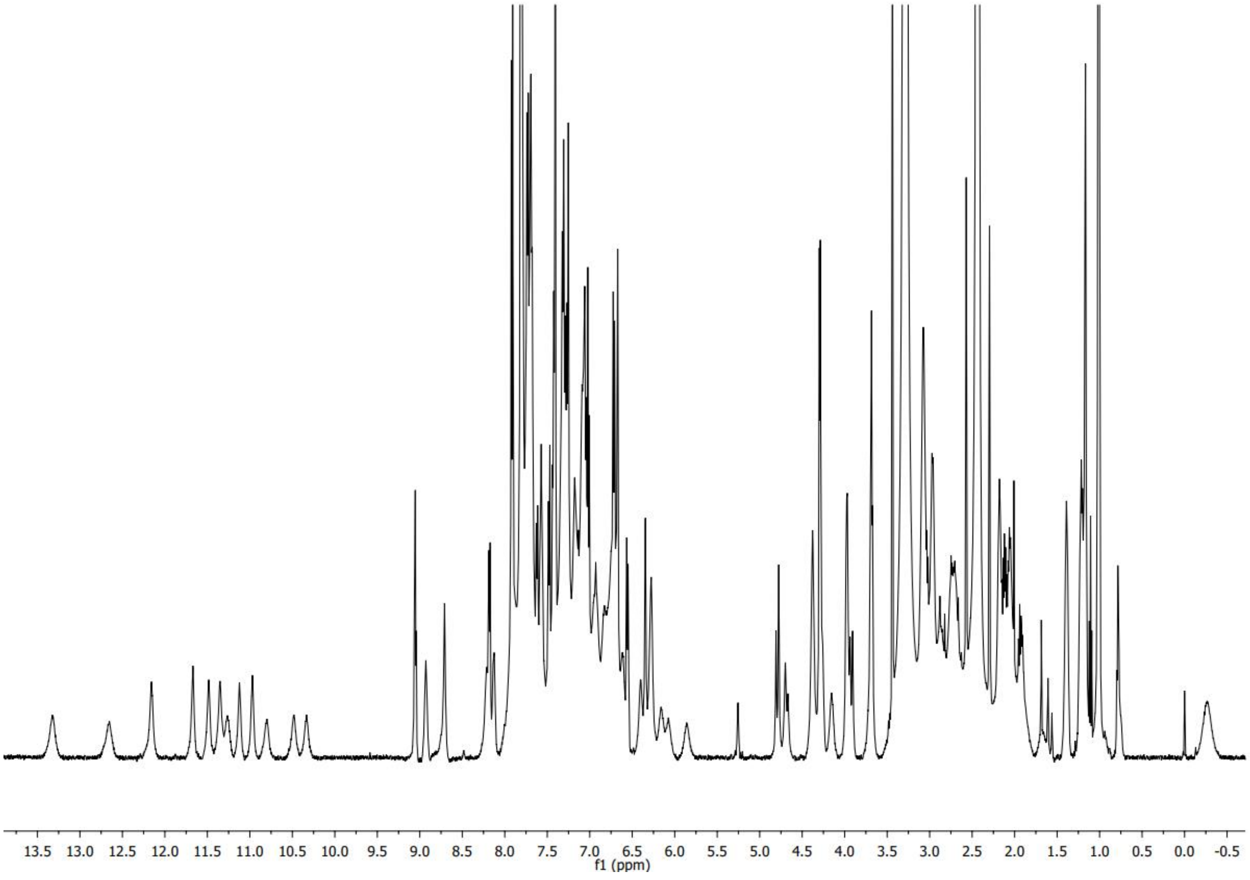

^{1}H NMR spectrum (500 MHz, DMSO-d_6, 25 °C)

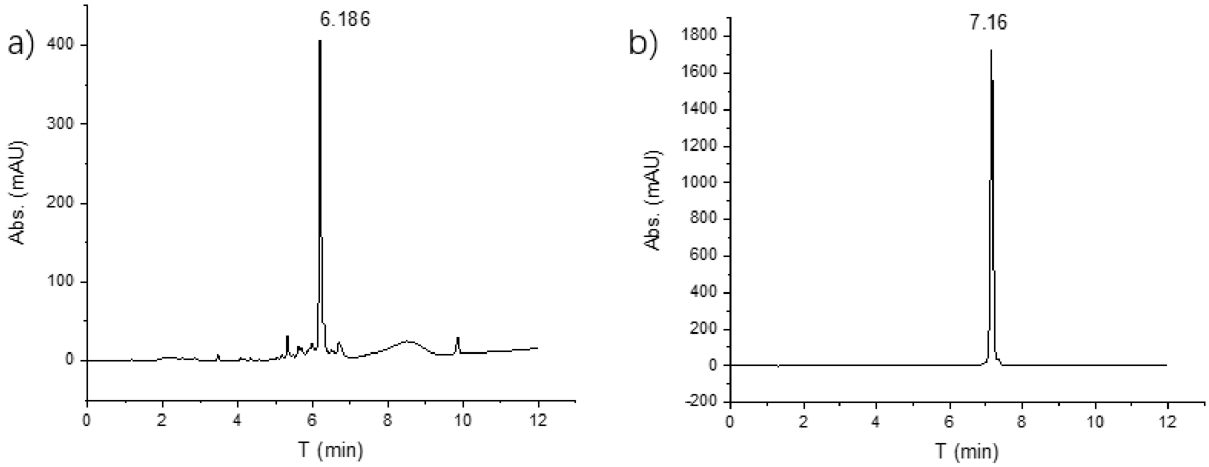

Figure S28. Analytical data of compound **16**. RP-HPLC chromatograms (a) after cleavage from the resin (C18, 10 to 100 B% over 10 min, 50 °C, λ = 254 nm) and (b) after purification (C18, 20 to 70 B% over 10 min, 50 °C, λ = 254 nm); A: 0.1% TFA water, B: 0.1% TFA acetonitrile.

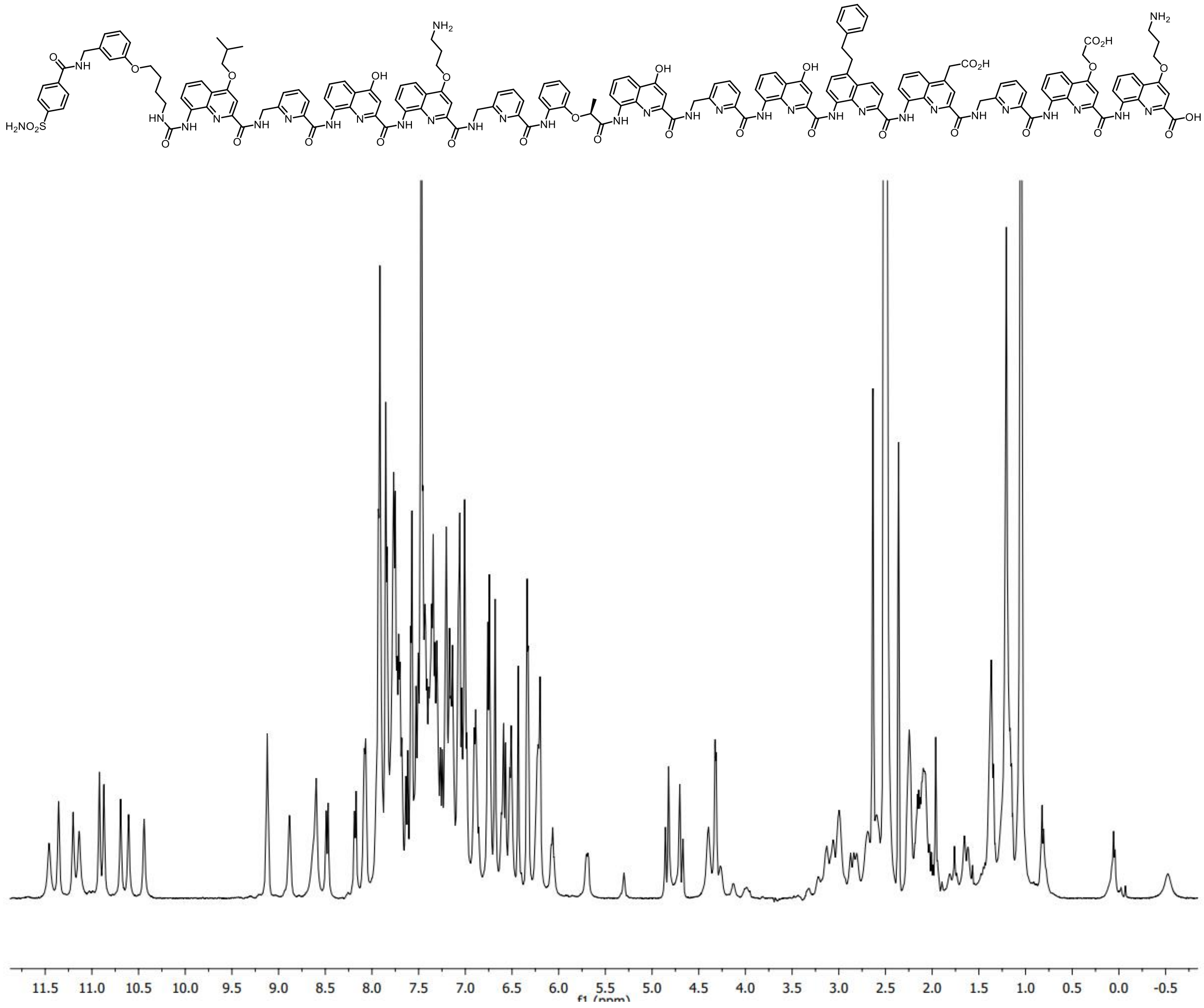

^{1}H NMR spectrum (500 MHz, DMSO-d_6/H_2O (9:1), 25 °C)

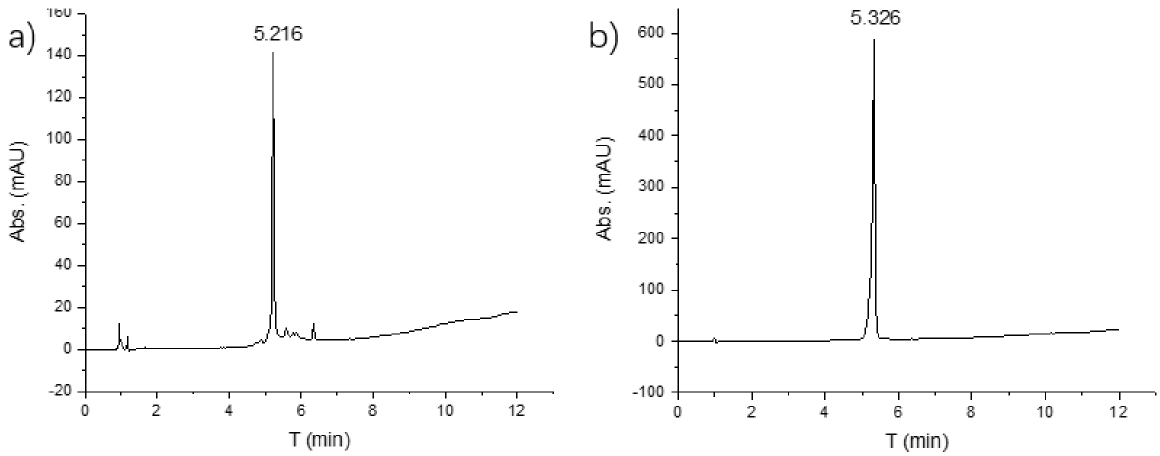

Figure S29. Analytical data of compound **17**. RP-HPLC chromatograms (a) after cleavage from the resin (C18, 10 to 100 B% over 10 min, 50 °C, λ = 254 nm) and (b) after purification (C18, 10 to 100 B% over 10 min, 50 °C, λ = 254 nm); A: 0.1% TFA water, B: 0.1% TFA acetonitrile.

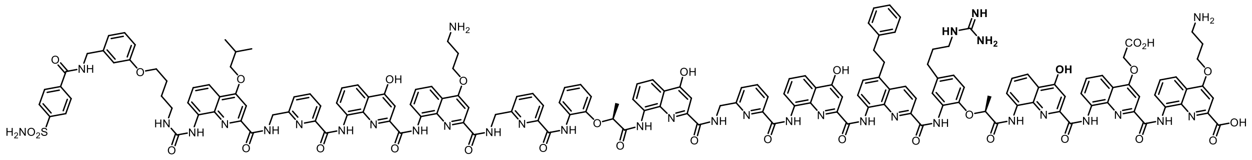

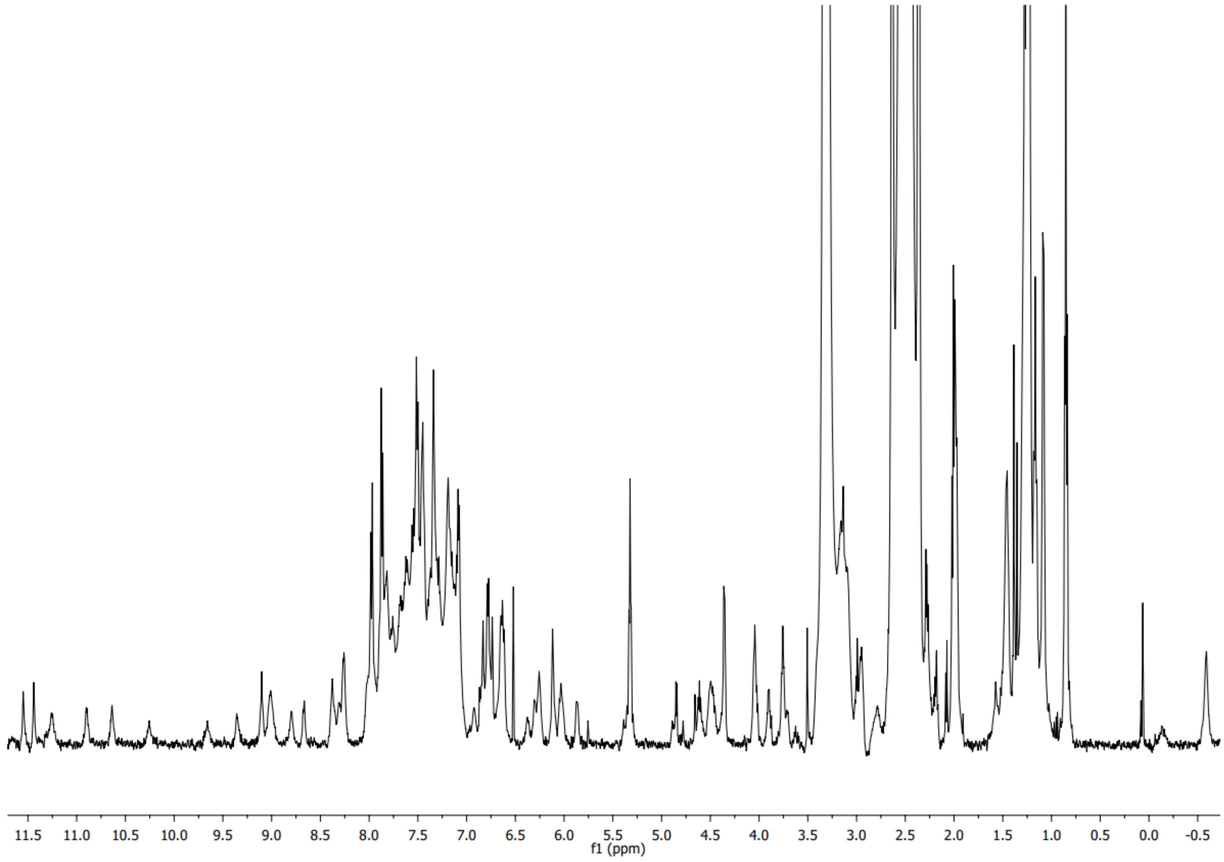

^{1}H NMR spectrum (500 MHz, DMSO-d_6, 25 °C)

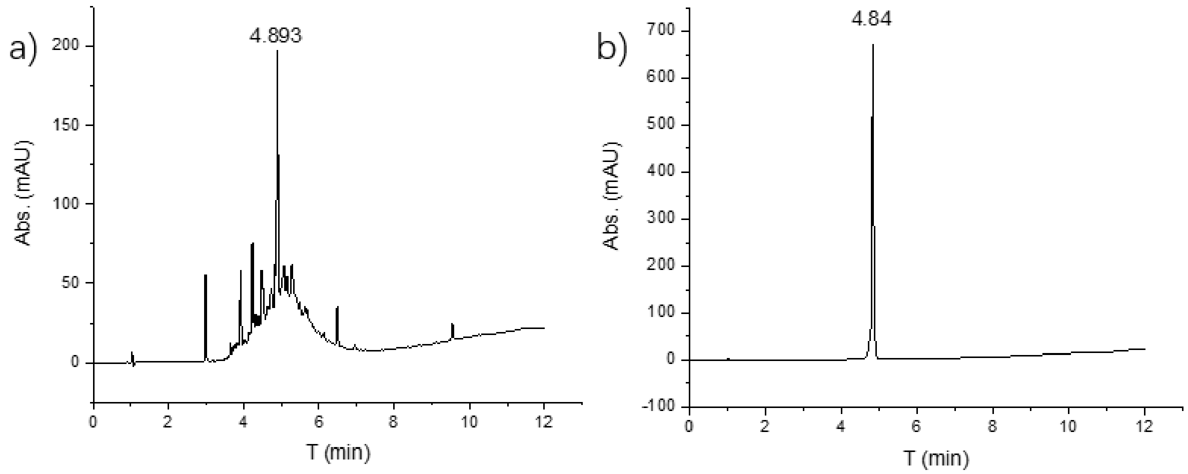

Figure S30. Analytical data of compound **18**. RP-HPLC chromatograms (a) after cleavage from the resin (C18, 10 to 100 B% over 10 min, 50 °C, λ = 254 nm) and (b) after purification (C18, 10 to 100 B% over 10 min, 50 °C, λ = 254 nm); A: 0.1% TFA water, B: 0.1% TFA acetonitrile.

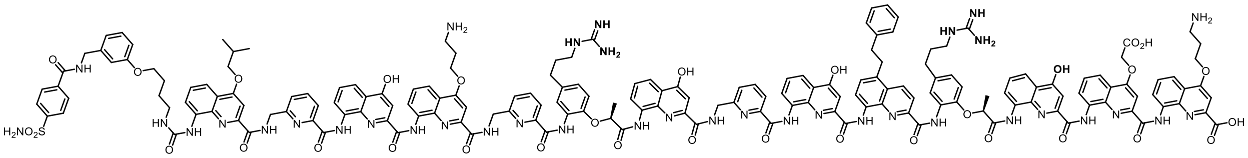

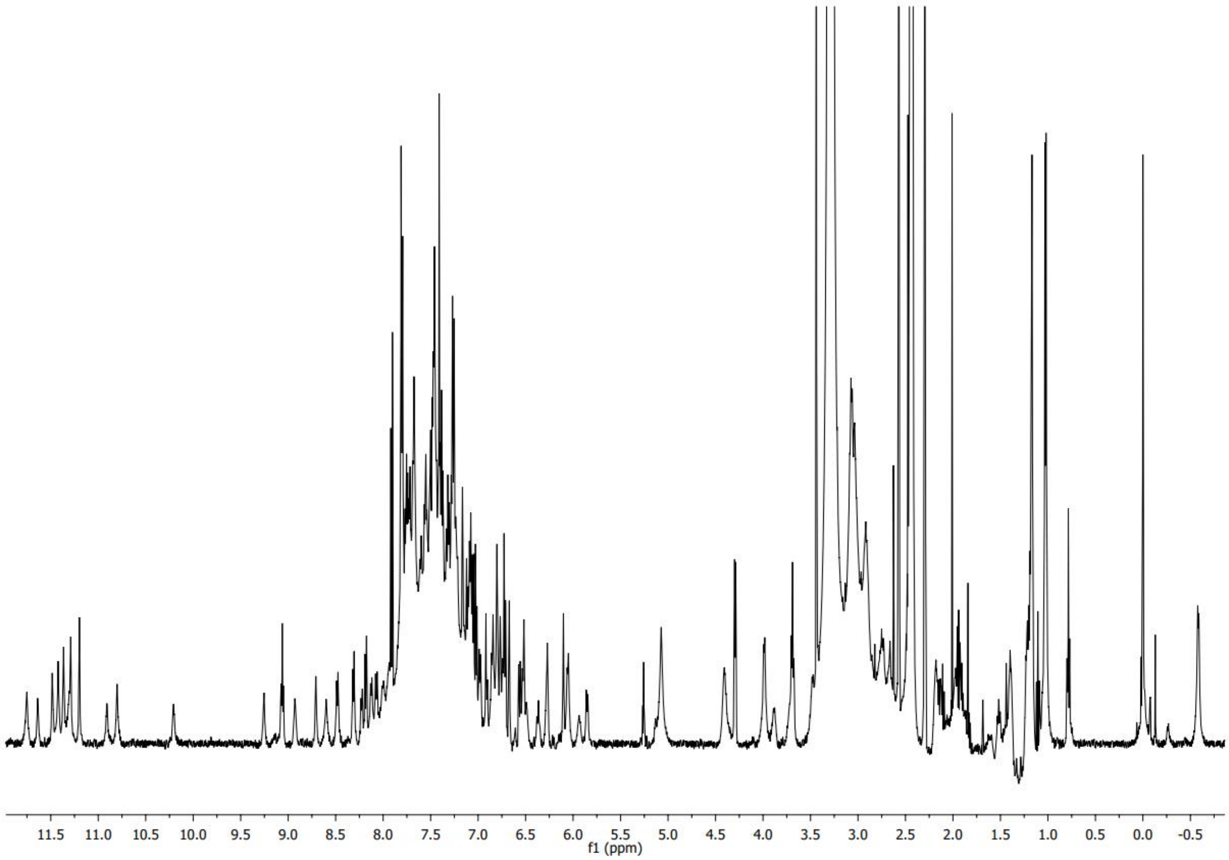

^{1}H NMR spectrum (500 MHz, DMSO-d_6, 25 °C)

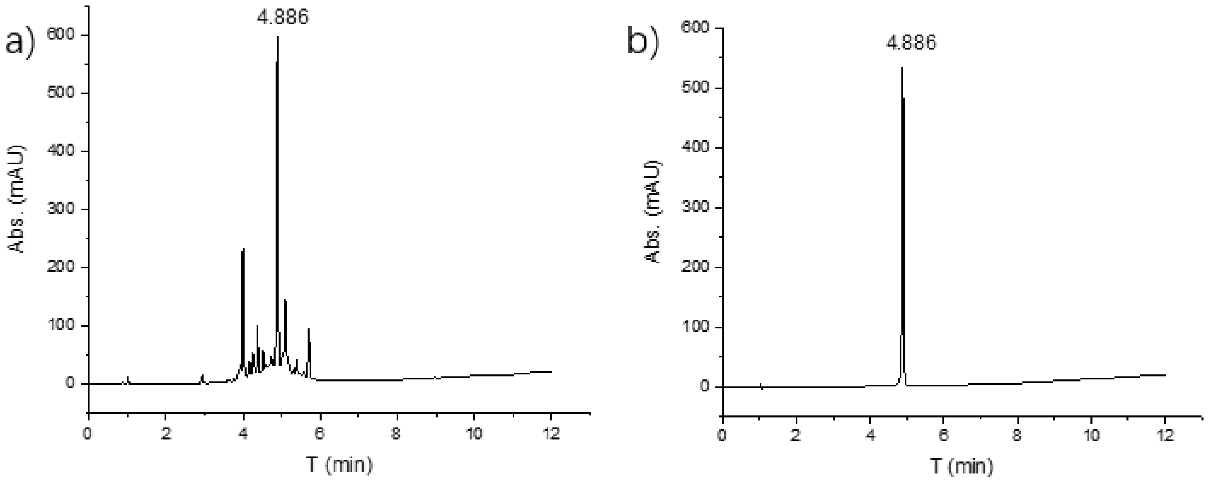

Figure S31. Analytical data of compound **19**. RP-HPLC chromatograms (a) after cleavage from the resin (C18, 10 to 100 B% over 10 min, 50 °C, λ = 254 nm) and (b) after purification (C18, 10 to 100 B% over 10 min, 50 °C, λ = 254 nm); A: 0.1% TFA water, B: 0.1% TFA acetonitrile.

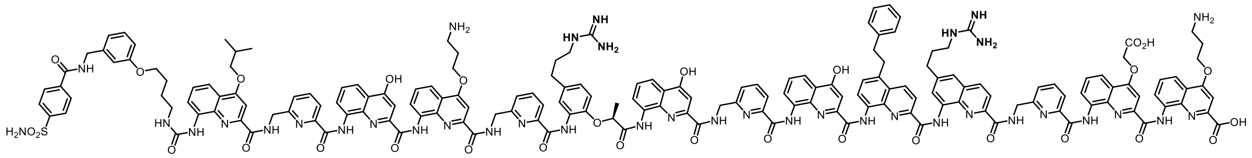

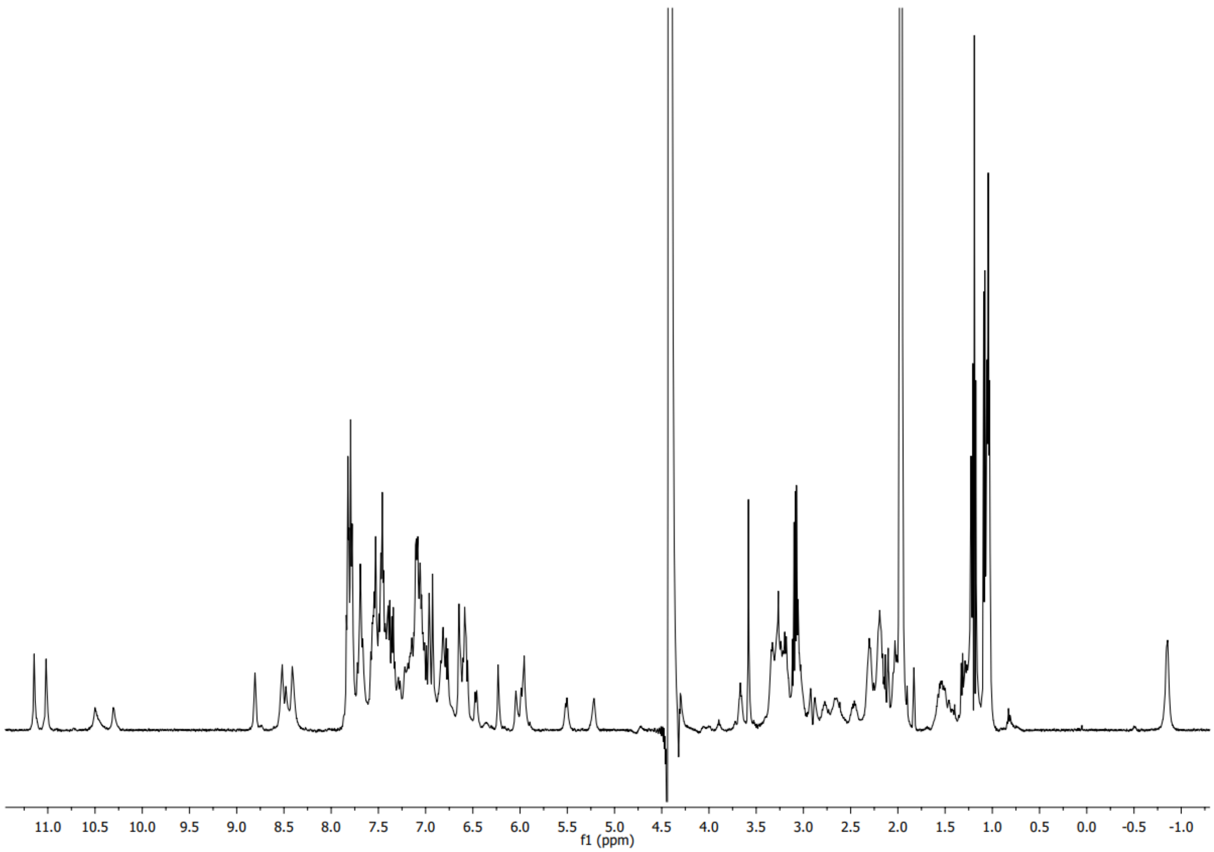

^{1}H NMR spectrum with water suppression (500 MHz, H_2O/CD_3CN (1:3, v/v), 25 °C).

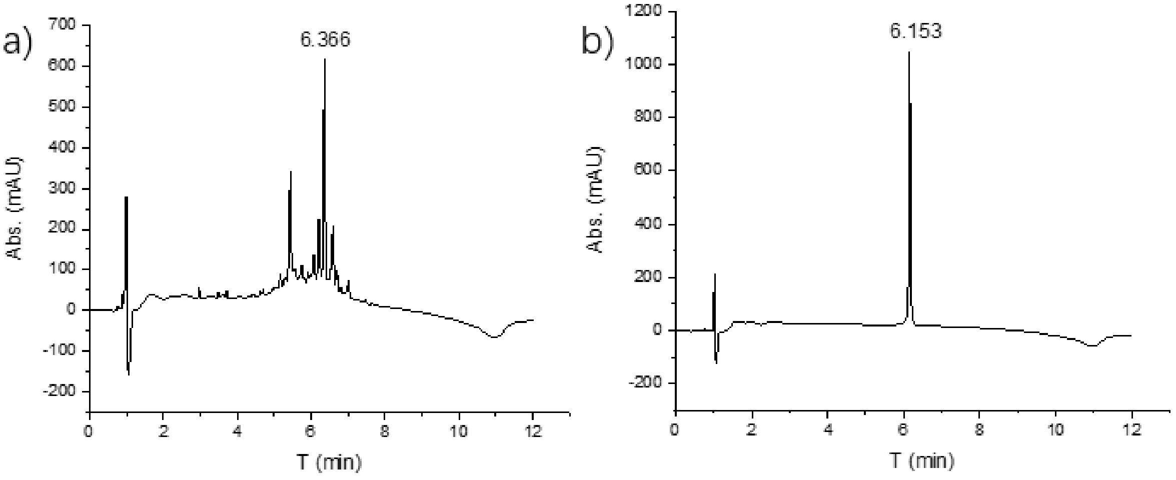

Figure S32. Analytical data of compound **20**. RP-HPLC chromatograms (a) after cleavage from the resin (C18, 10 to 100 B% over 10 min, 50 °C, λ = 254 nm) and (b) after purification (C18, 10 to 100 B% over 10 min, λ = 254 nm); A: 0.1% TFA water, B: 0.1% TFA acetonitrile.

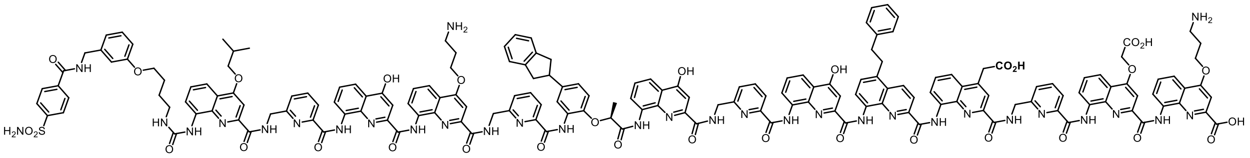

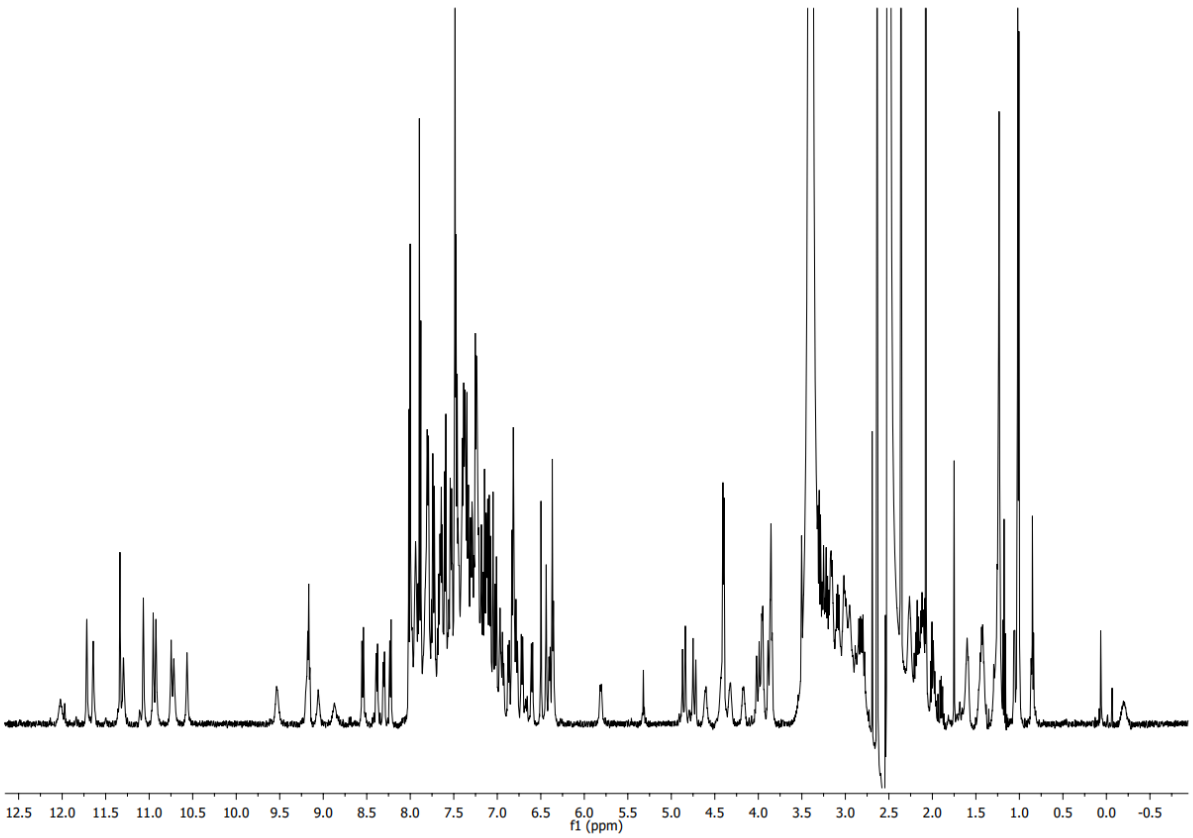

^{1}H NMR spectrum (500 MHz, DMSO-d_6, 25 °C)

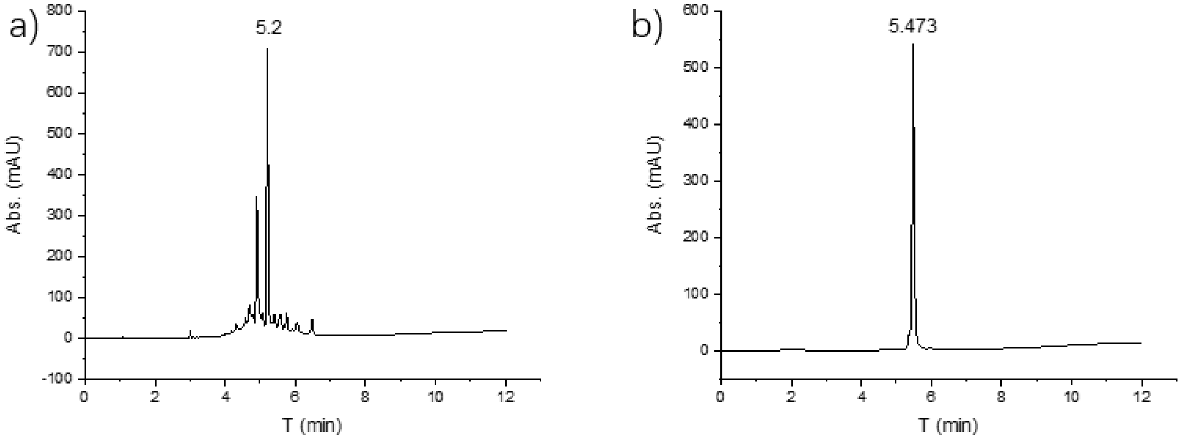

Figure S33. Analytical data of compound **21**. RP-HPLC chromatograms (a) after cleavage from the resin (C18, 10 to 100 B% over 10 min, 50 °C, λ = 254 nm) and (b) after purification (C18, 10 to 100 B% over 10 min, λ = 254 nm); A: 0.1% TFA water, B: 0.1% TFA acetonitrile

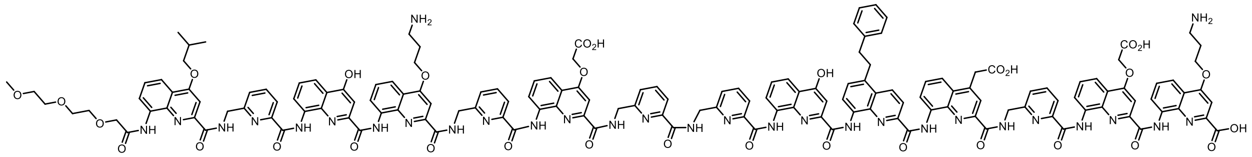

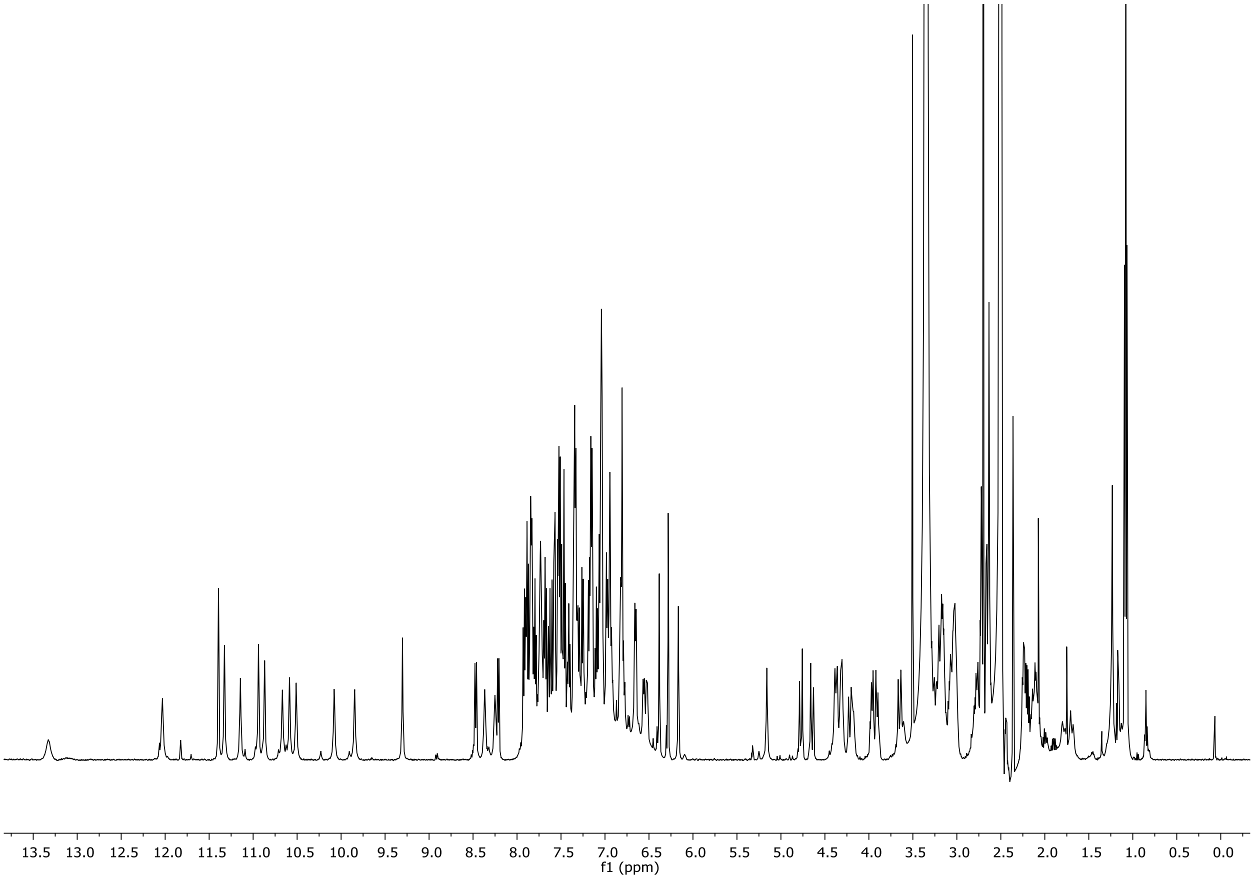

^{1}H NMR spectrum (500 MHz, DMSO-d_6, 25 °C)

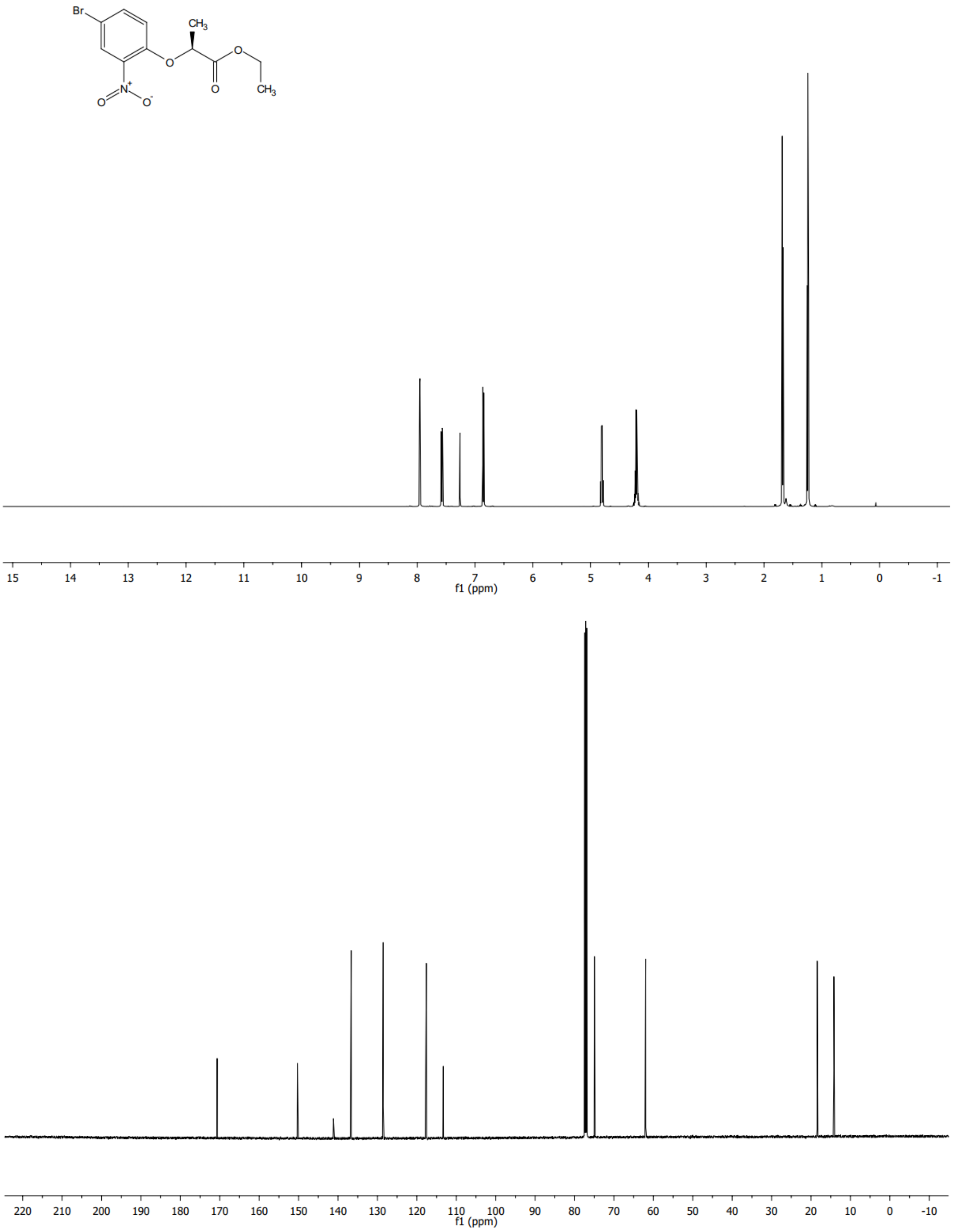

Figure S34. NMR spectra of **25**: ^{1}H NMR (500 MHz, DMSO-*d*6) and ^{13}C NMR (126 MHz, DMSO-*d*6).

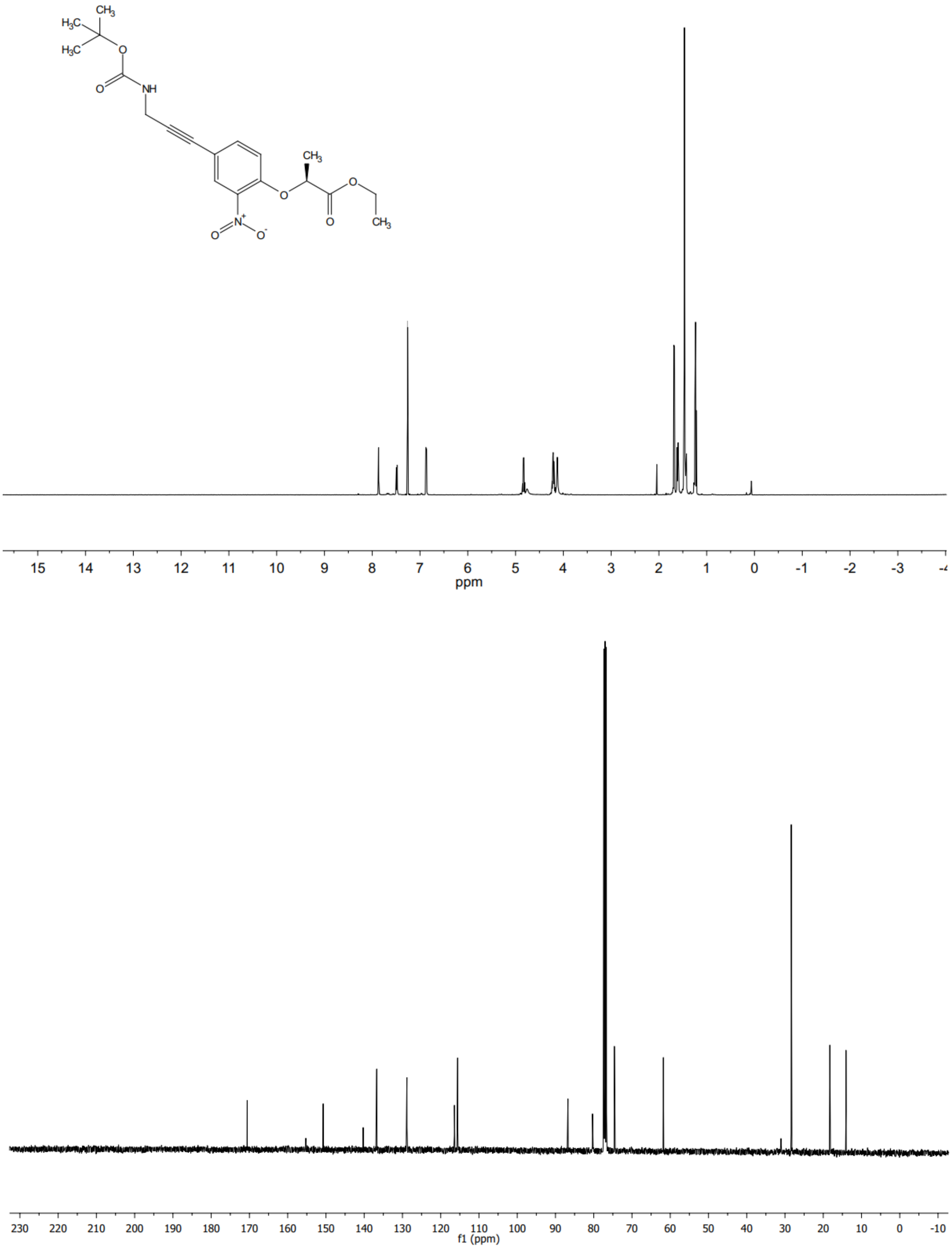

Figure S35. NMR spectra of **26**: ^{1}H NMR (400 MHz, $CDCl_3$) and ^{13}C NMR (101 MHz, $CDCl_3$)

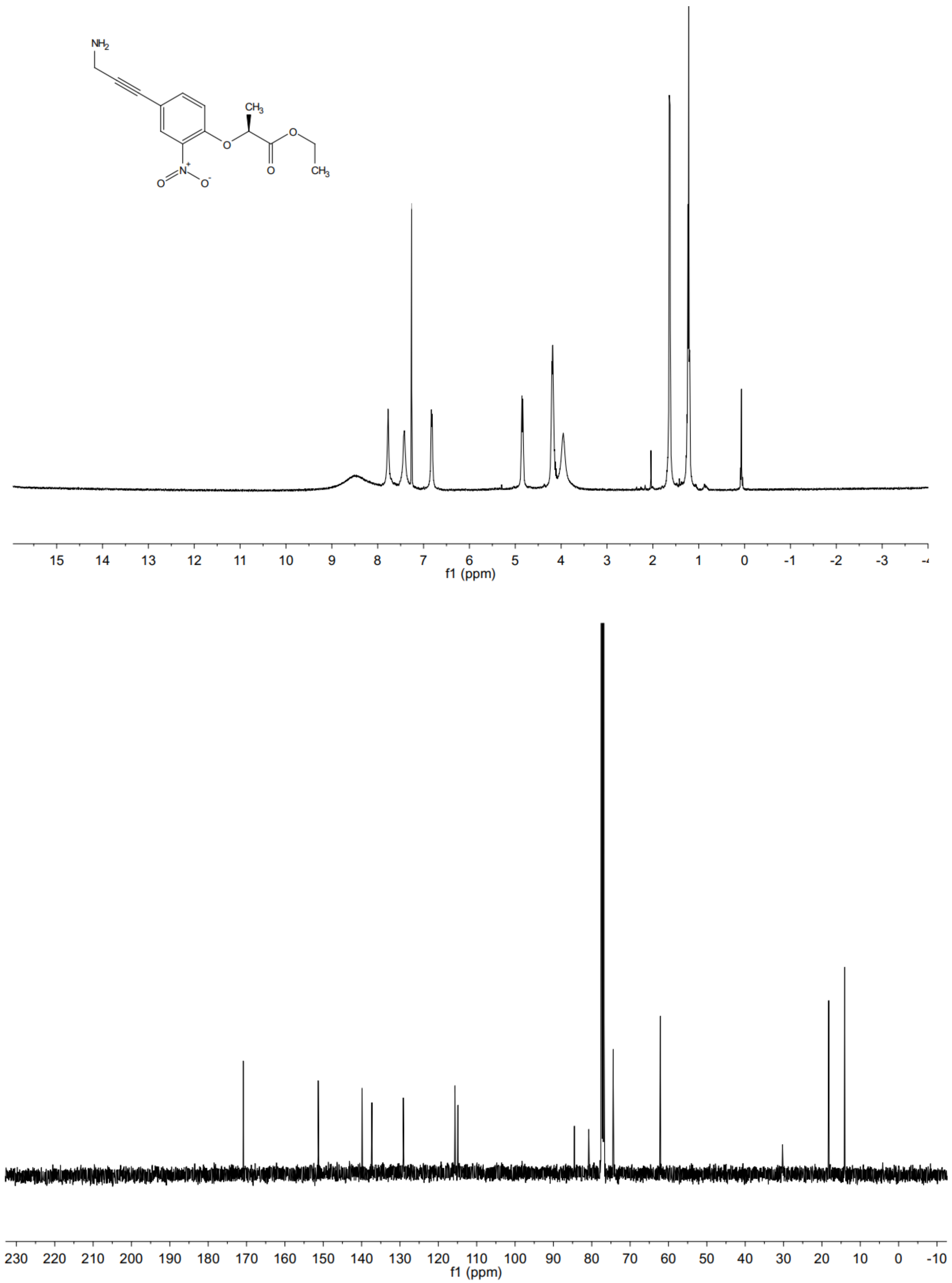

Figure S36. NMR spectra of **27**: ^{1}H NMR (400 MHz, $CDCl_3$) and ^{13}C NMR (101 MHz, $CDCl_3$).

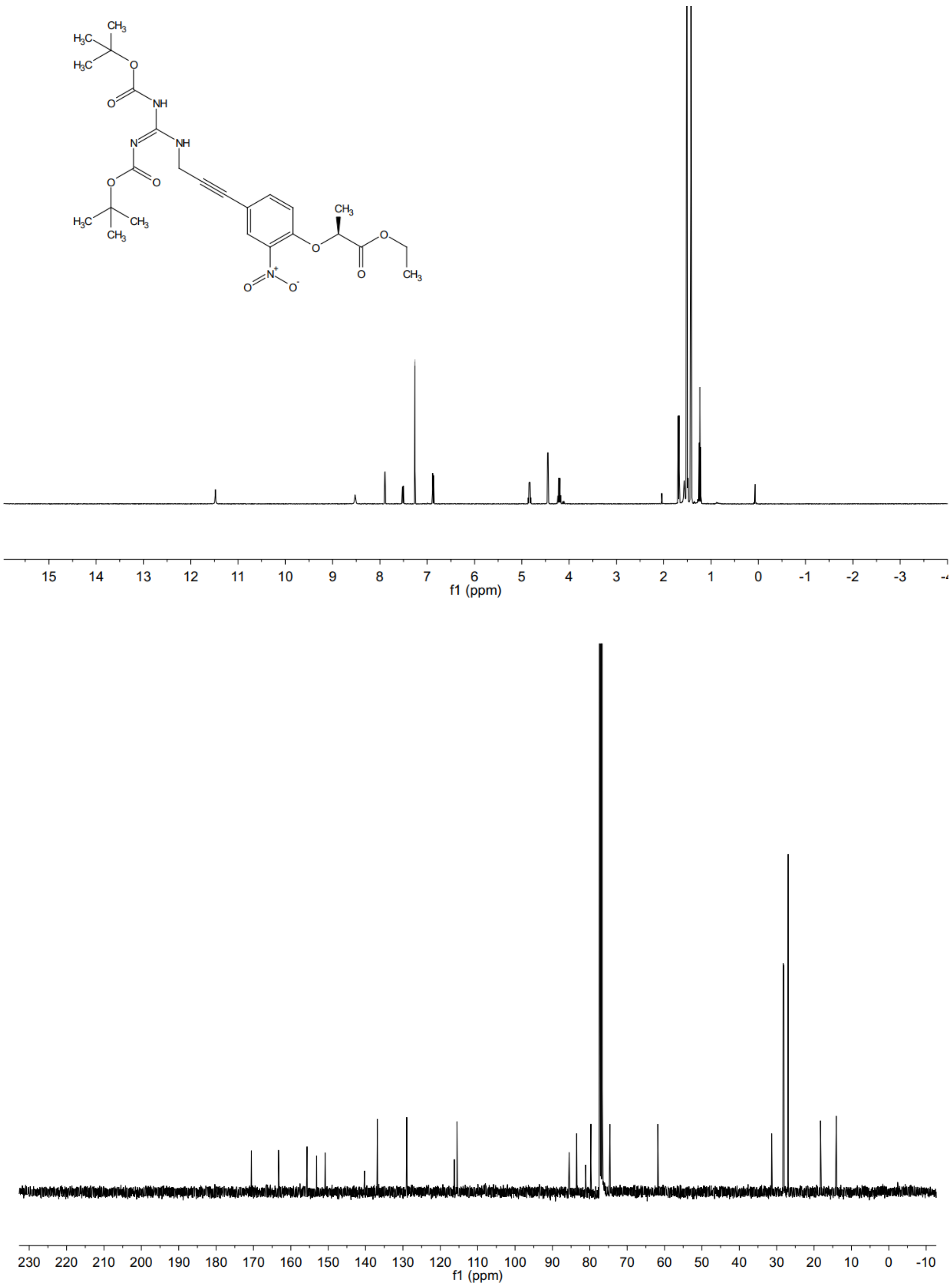

Figure S37. NMR spectra of **28**: ^{1}H NMR (400 MHz, $CDCl_3$) and ^{13}C NMR (101 MHz, $CDCl_3$).

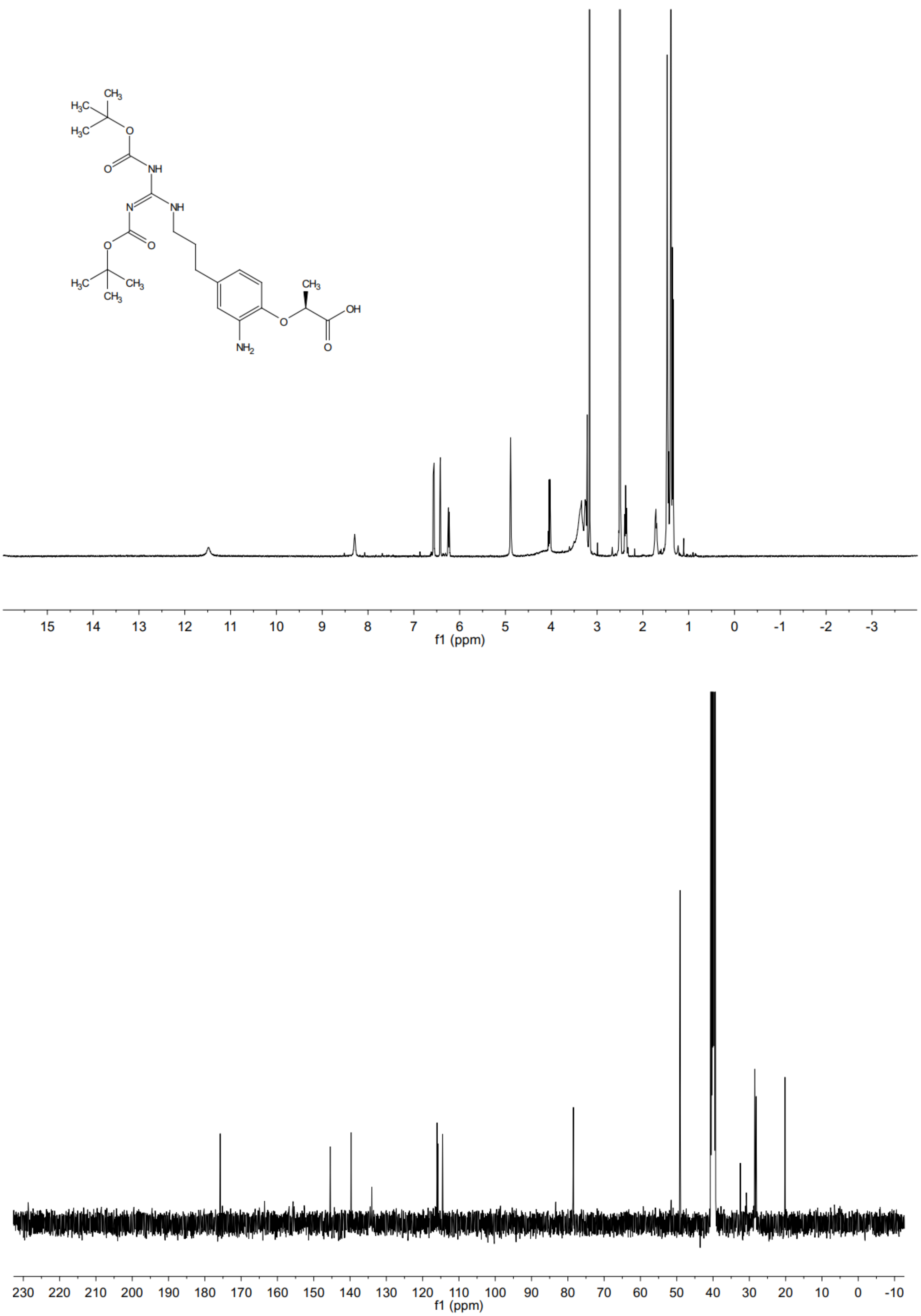

Figure S38. NMR spectra of **29**: ^{1}H NMR (400 MHz, $CDCl_3$) and ^{13}C NMR (101 MHz, $CDCl_3$).

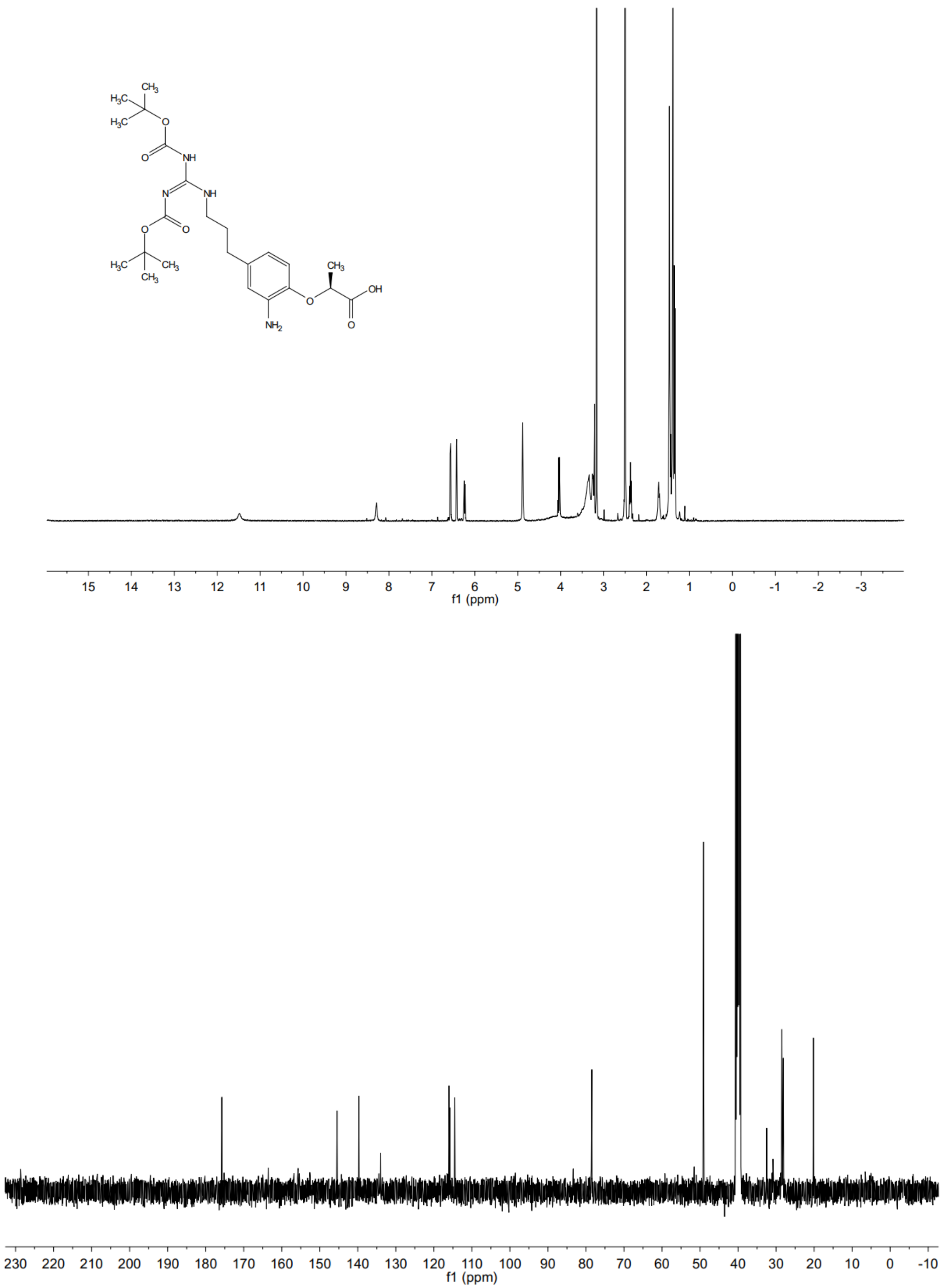

Figure S39. NMR spectra of **30**: ^{1}H NMR (400 MHz, DMSO-d_6) and ^{13}C NMR (101 MHz, DMSO-d_6)

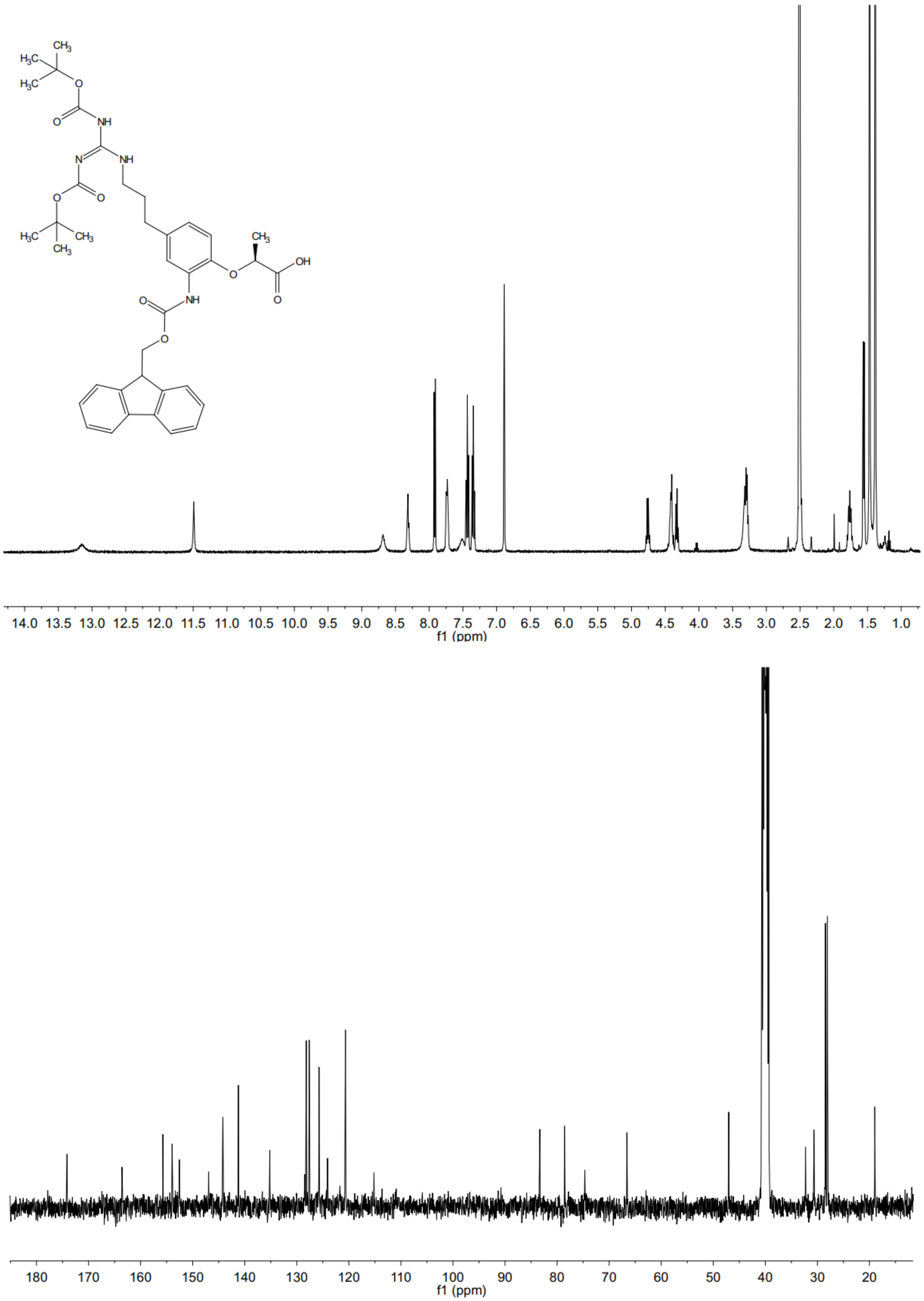

Figure S40. NMR spectra of **31**: ^{1}H NMR (400 MHz, DMSO-d_6) and ^{13}C NMR (101 MHz, DMSO-d_6)

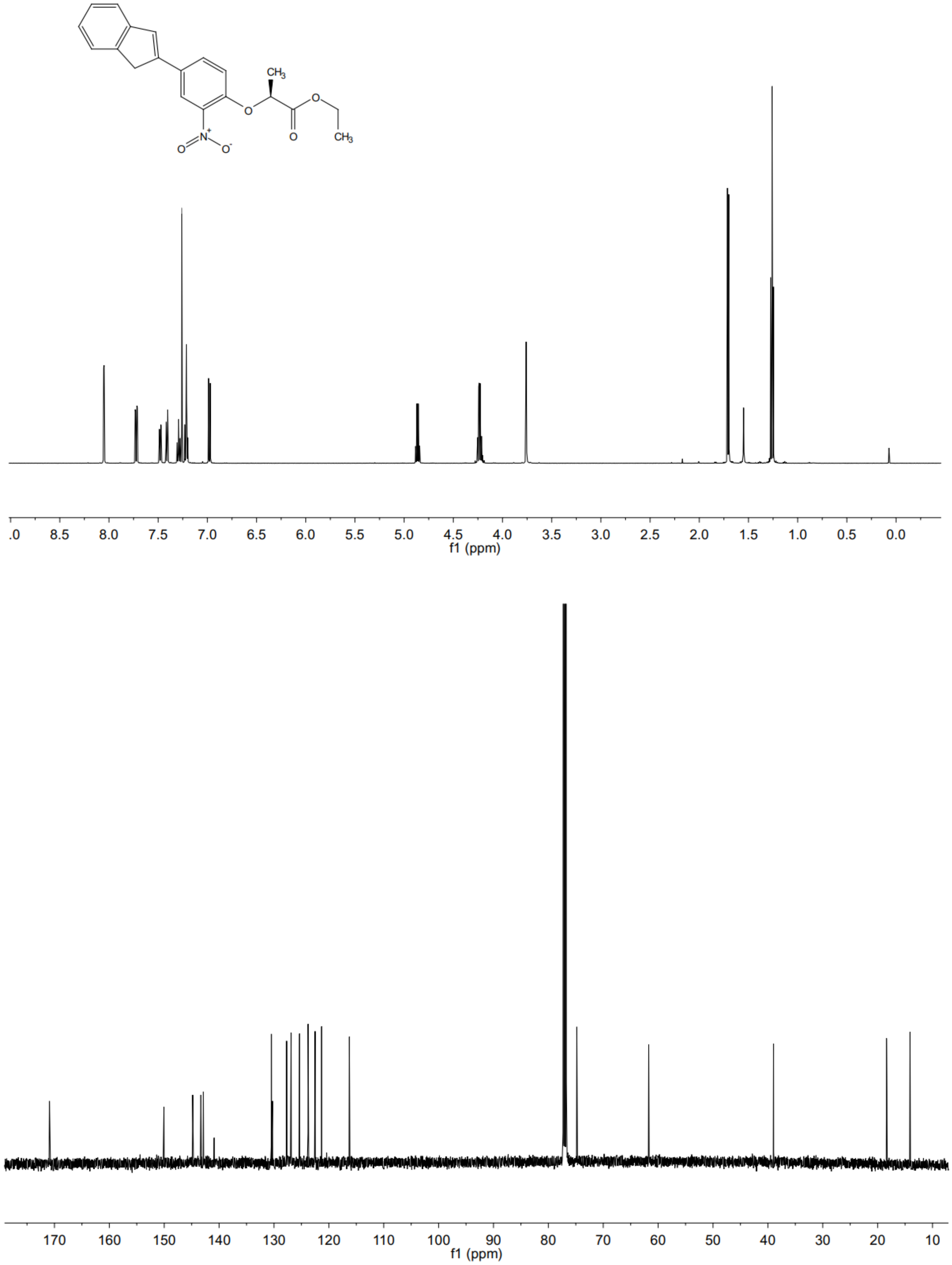

Figure S41. NMR spectra of **32**: ^{1}H NMR (400 MHz, $CDCl_3$) and ^{13}C NMR (101 MHz, $CDCl_3$).

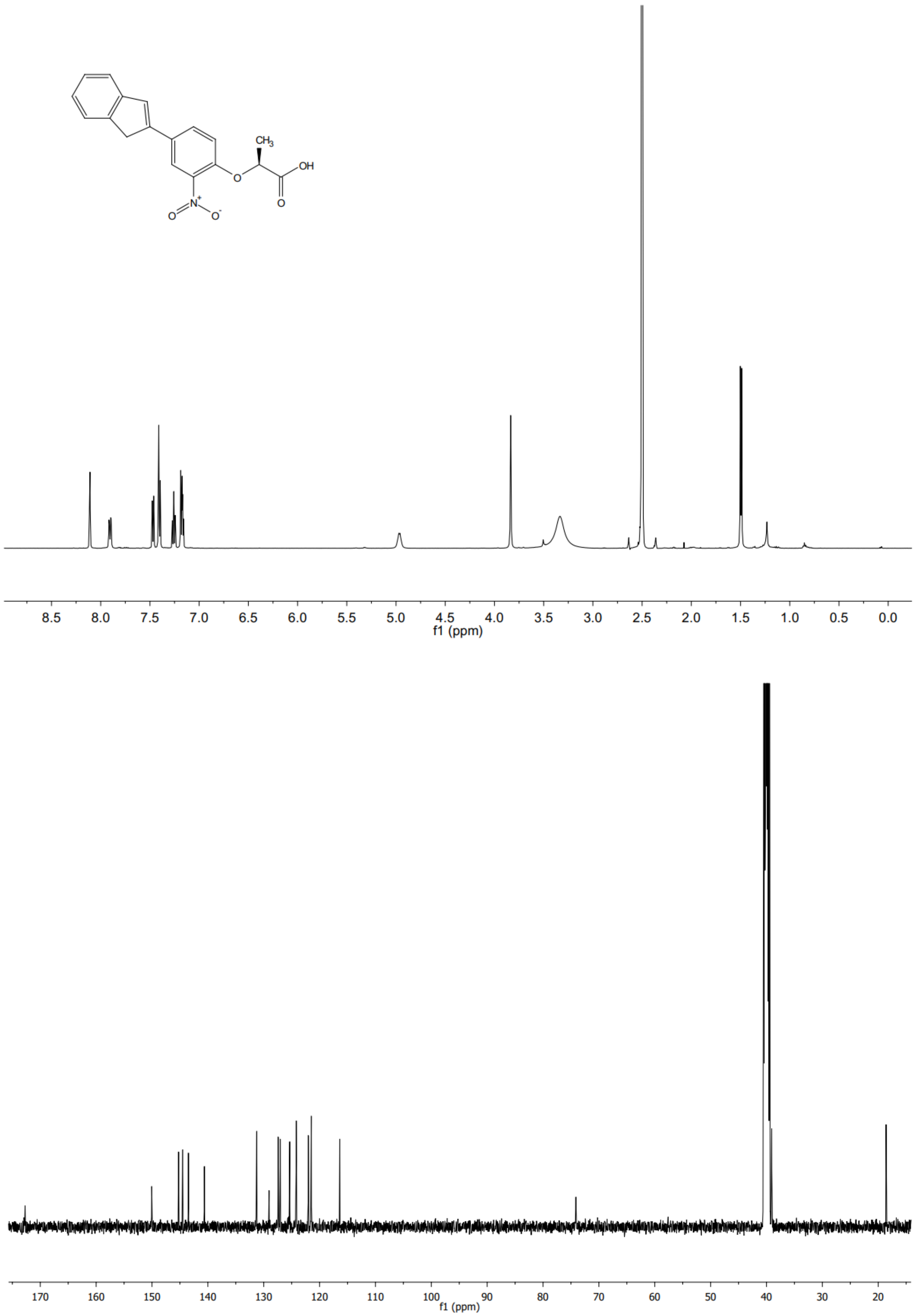

Figure S42. NMR spectra of **33**: ^{1}H NMR (400 MHz, DMSO-d_6) and ^{13}C NMR (101 MHz, DMSO-d_6).

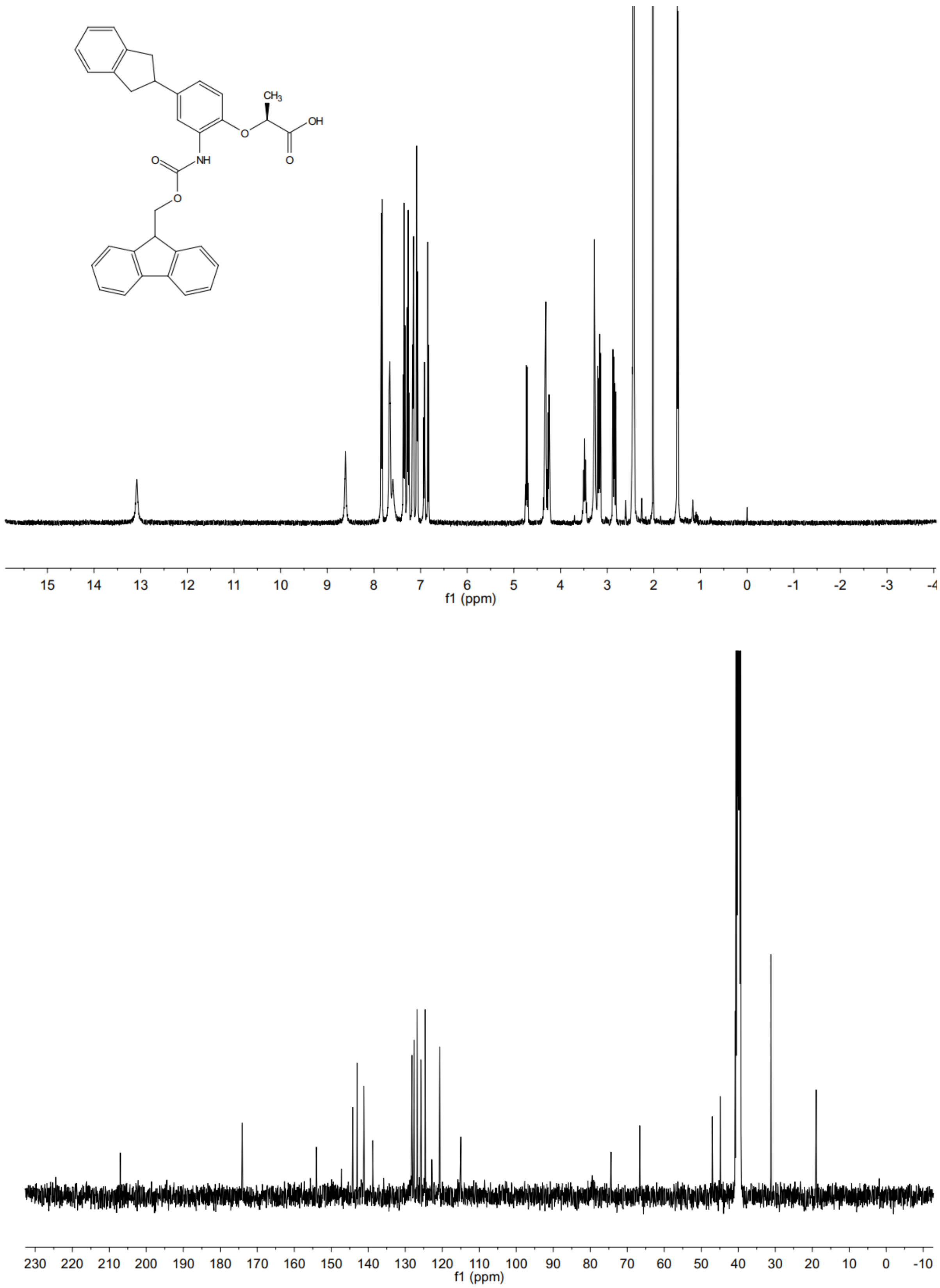

Figure S43. NMR spectra of **35**: ^{1}H NMR (400 MHz, DMSO-d_6) and ^{13}C NMR (101 MHz, DMSO-d_6).

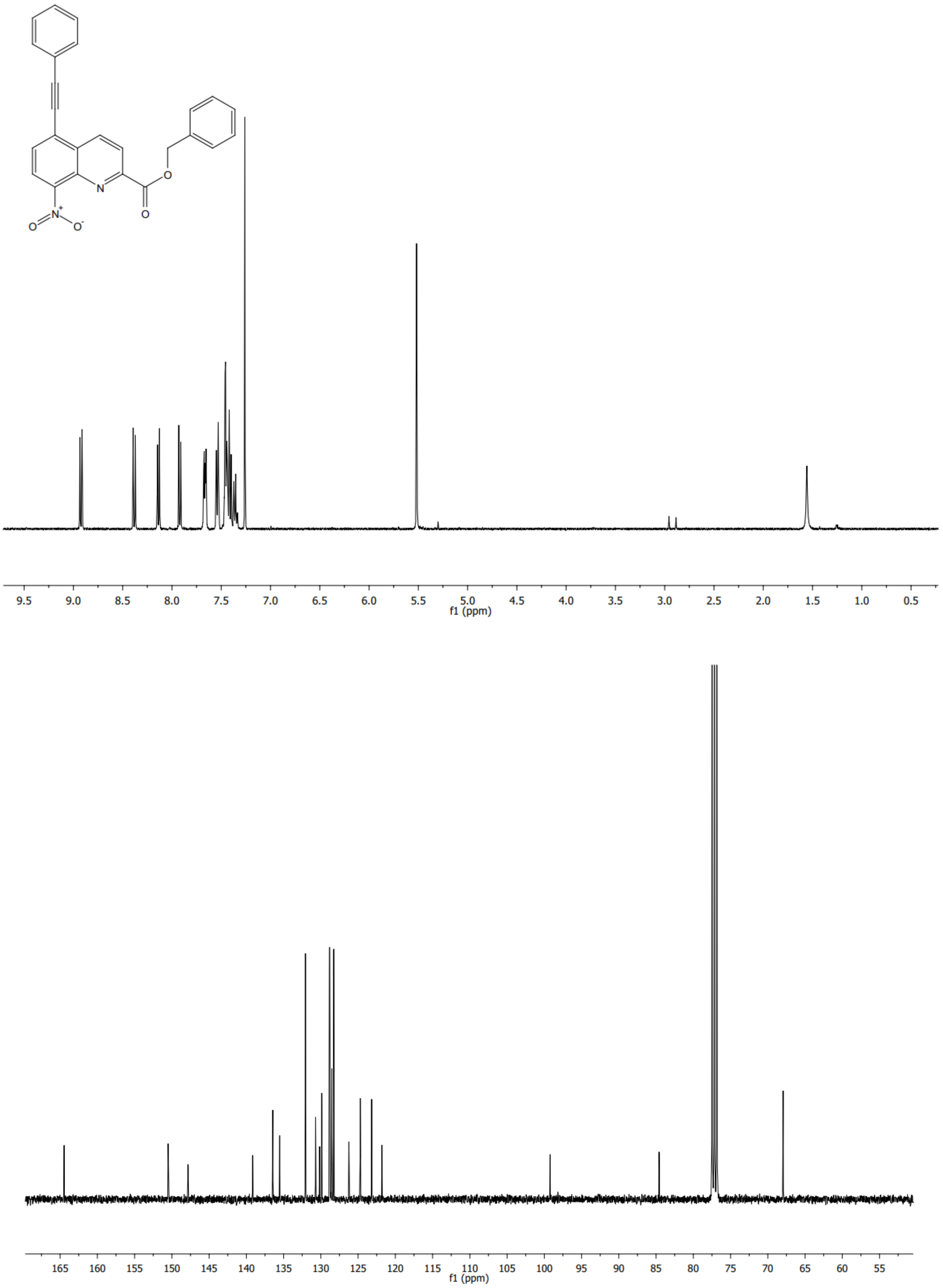

Figure S44. NMR spectra of **37**: ^{1}H NMR (400 MHz, $CDCl_3$) and ^{13}C NMR (101 MHz, $CDCl_3$).

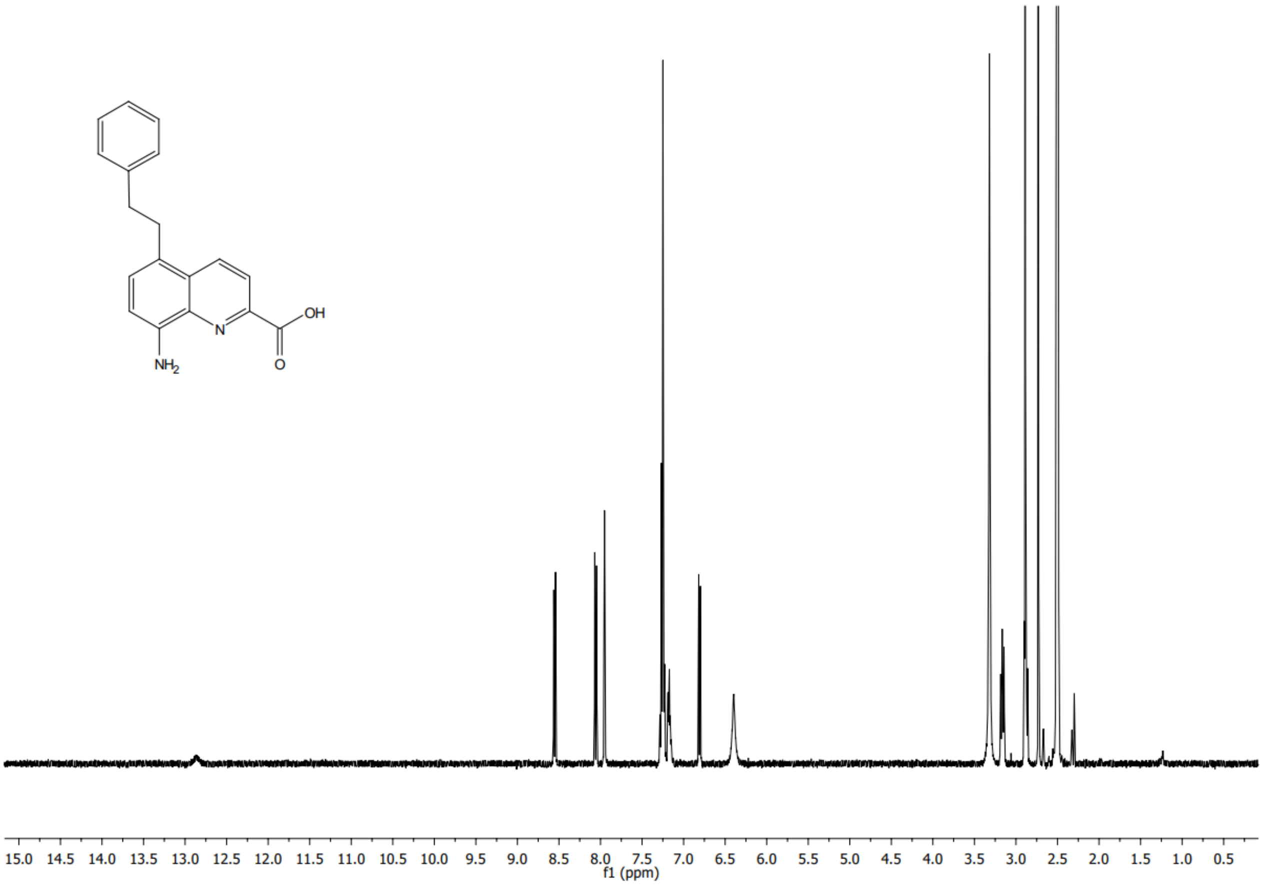

Figure S45. NMR spectra of **38**: ^{1}H NMR (400 MHz, DMSO-d_6).

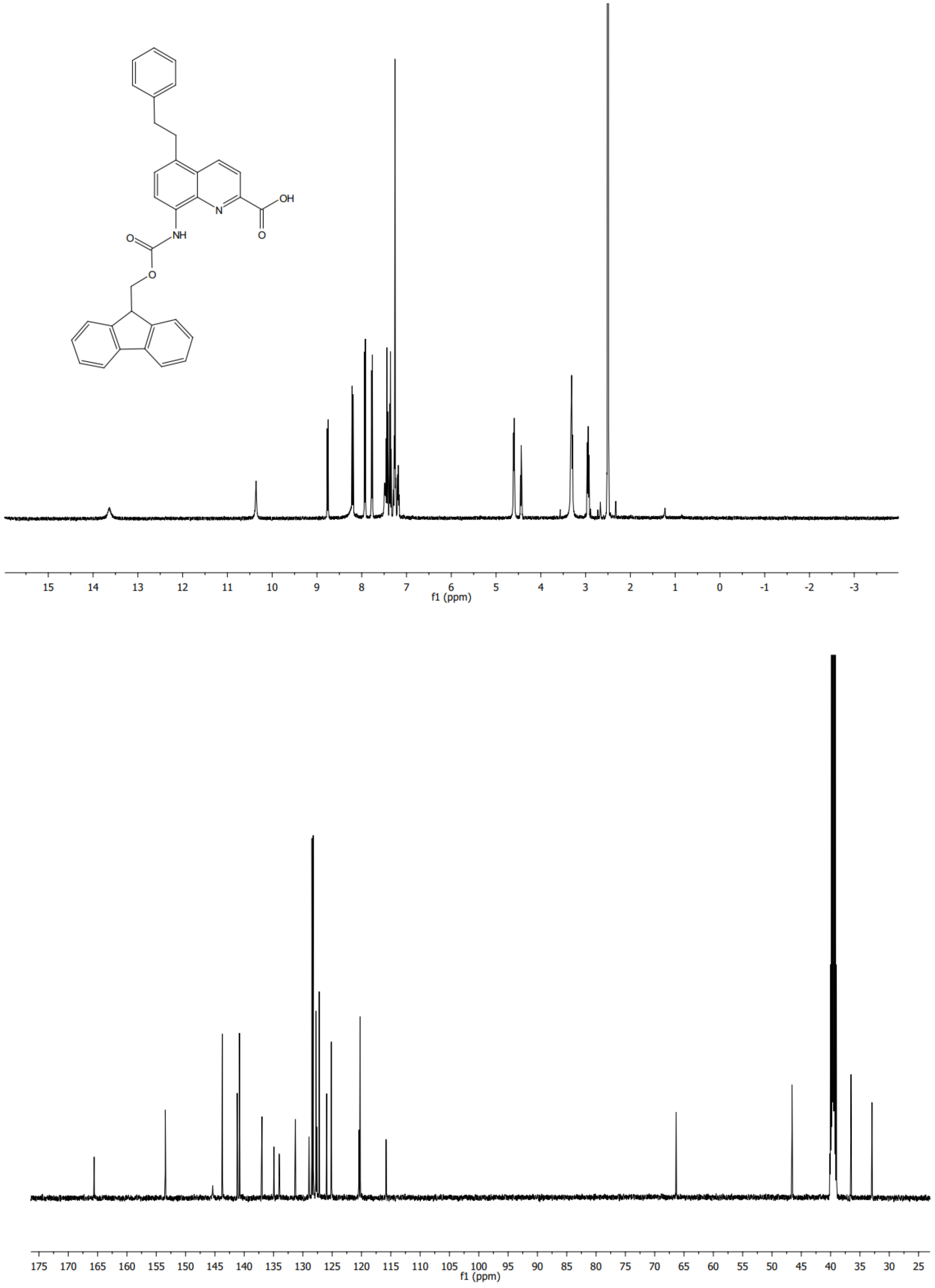

Figure S46. NMR spectra of **39**: ^{1}H NMR (400 MHz, DMSO-d_6) and ^{13}C NMR (101 MHz, DMSO-d_6).

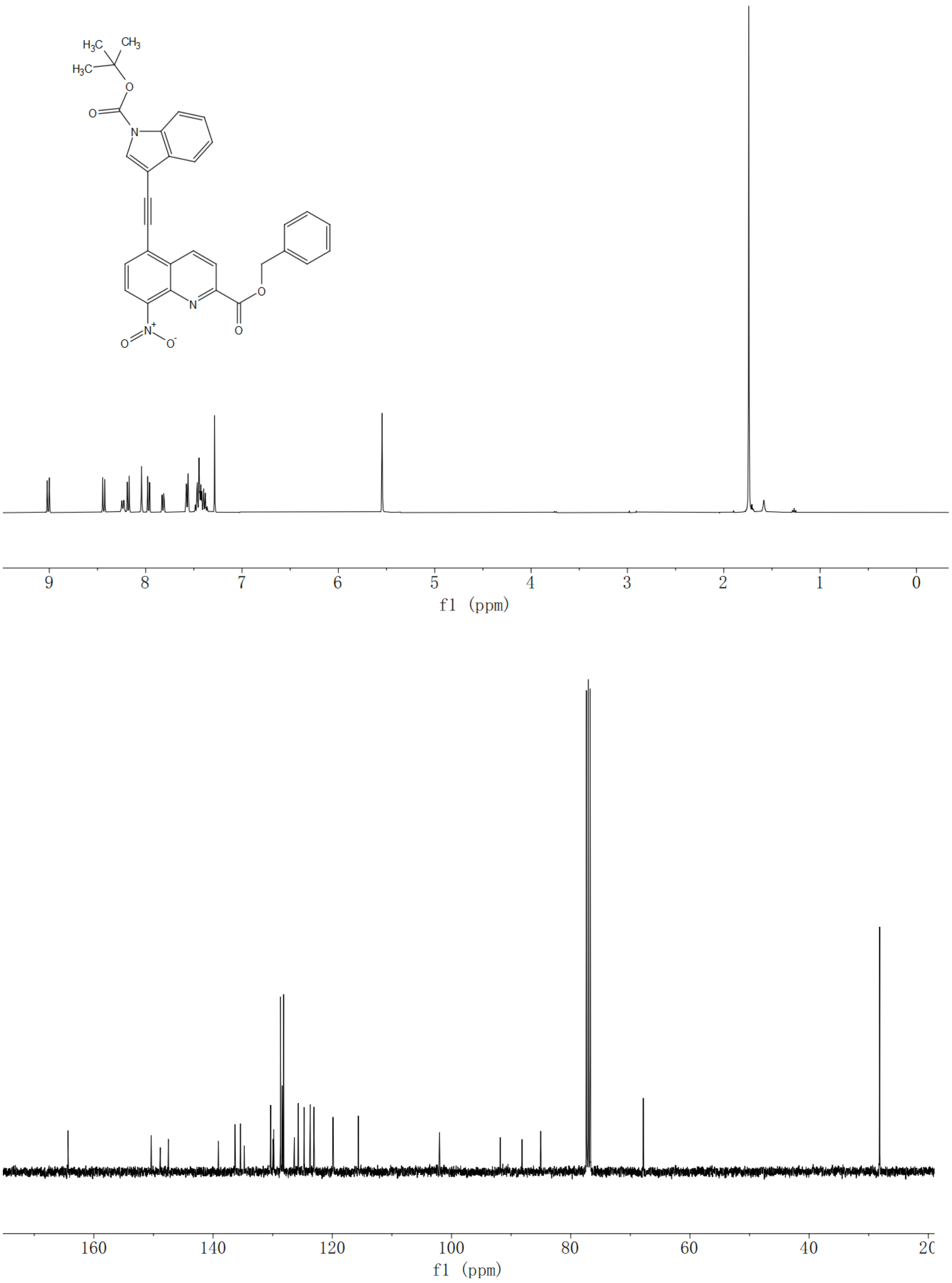

Figure S47 NMR spectra of **41**: ^{1}H NMR (400 MHz, $CDCl_3$) and ^{13}C NMR (101 MHz, $CDCl_3$).

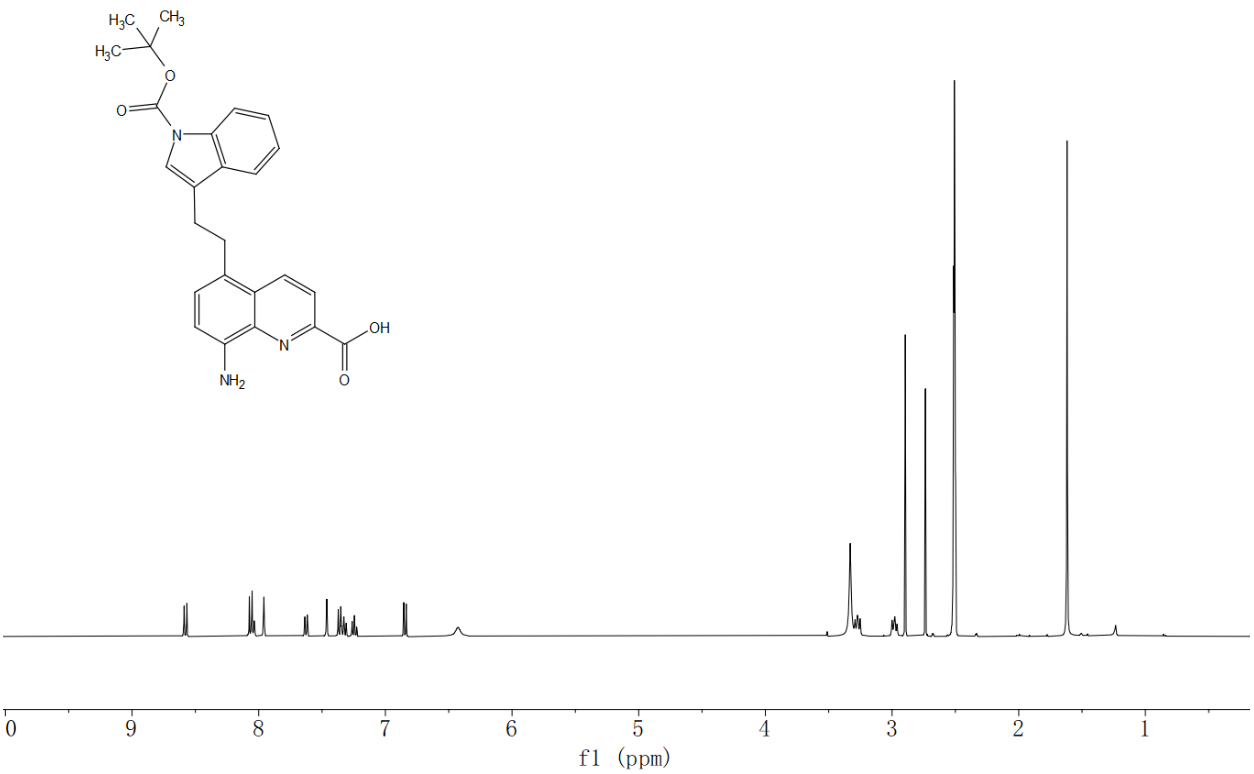

Figure S48 NMR spectra of **42**: ^{1}H NMR (400 MHz, DMSO-d_6)

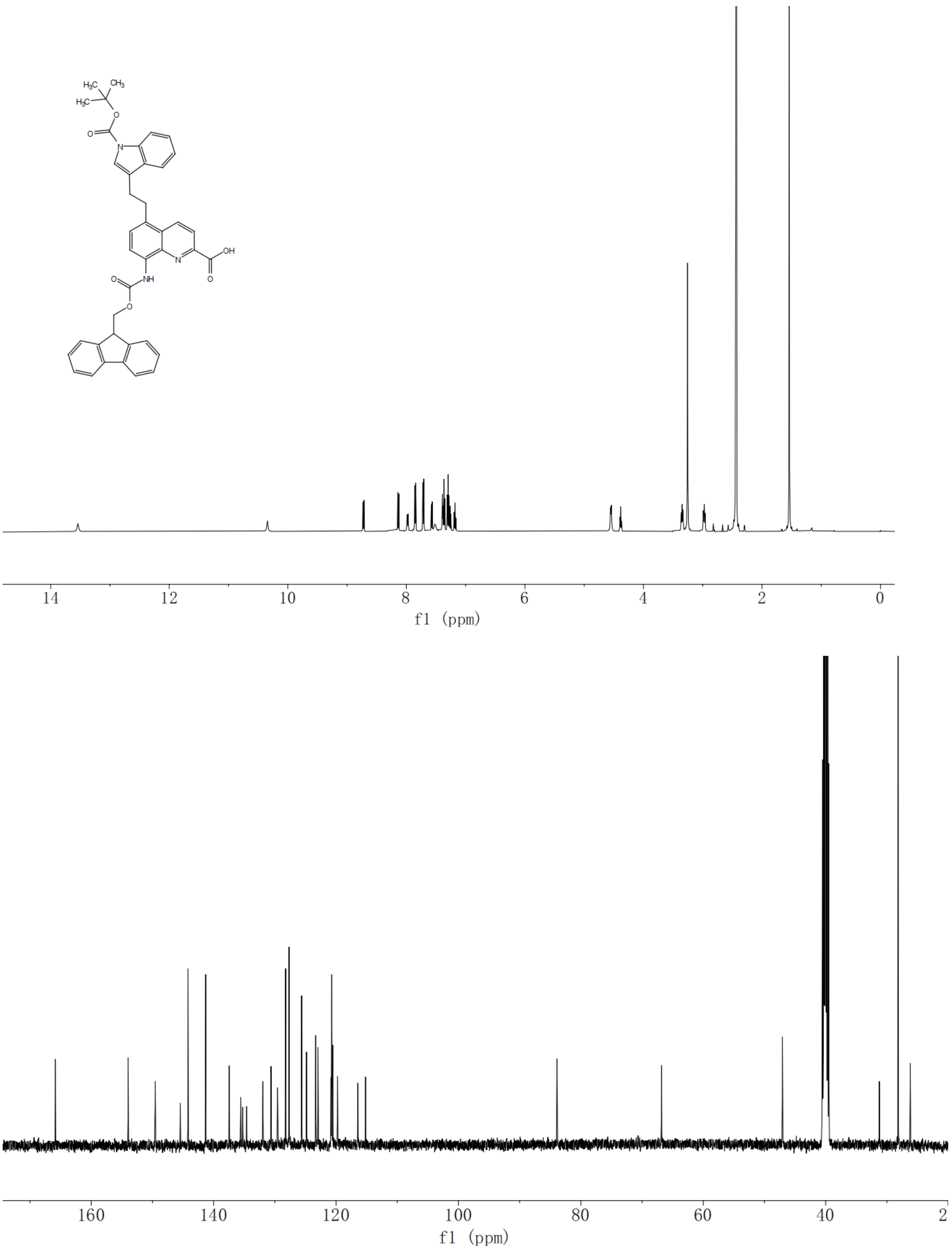

Figure S49 NMR spectra of **43**: ^{1}H NMR (400 MHz, DMSO-d_6) and ^{13}C NMR (101 MHz, DMSO-d_6).

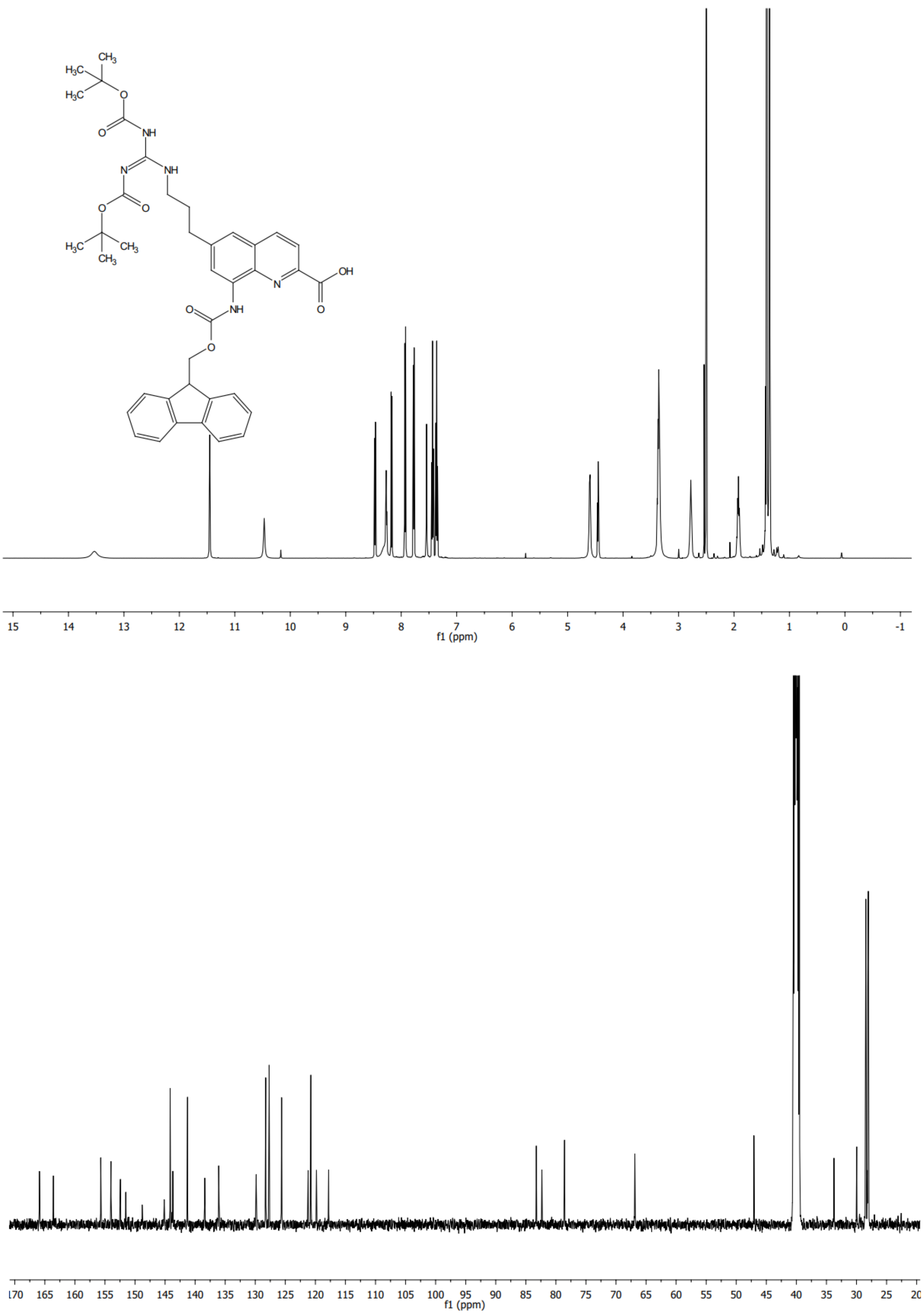

Figure S50. NMR spectra of **45**: ^{1}H NMR (400 MHz, DMSO-d_6) and ^{13}C NMR (101 MHz, DMSO-d_6).

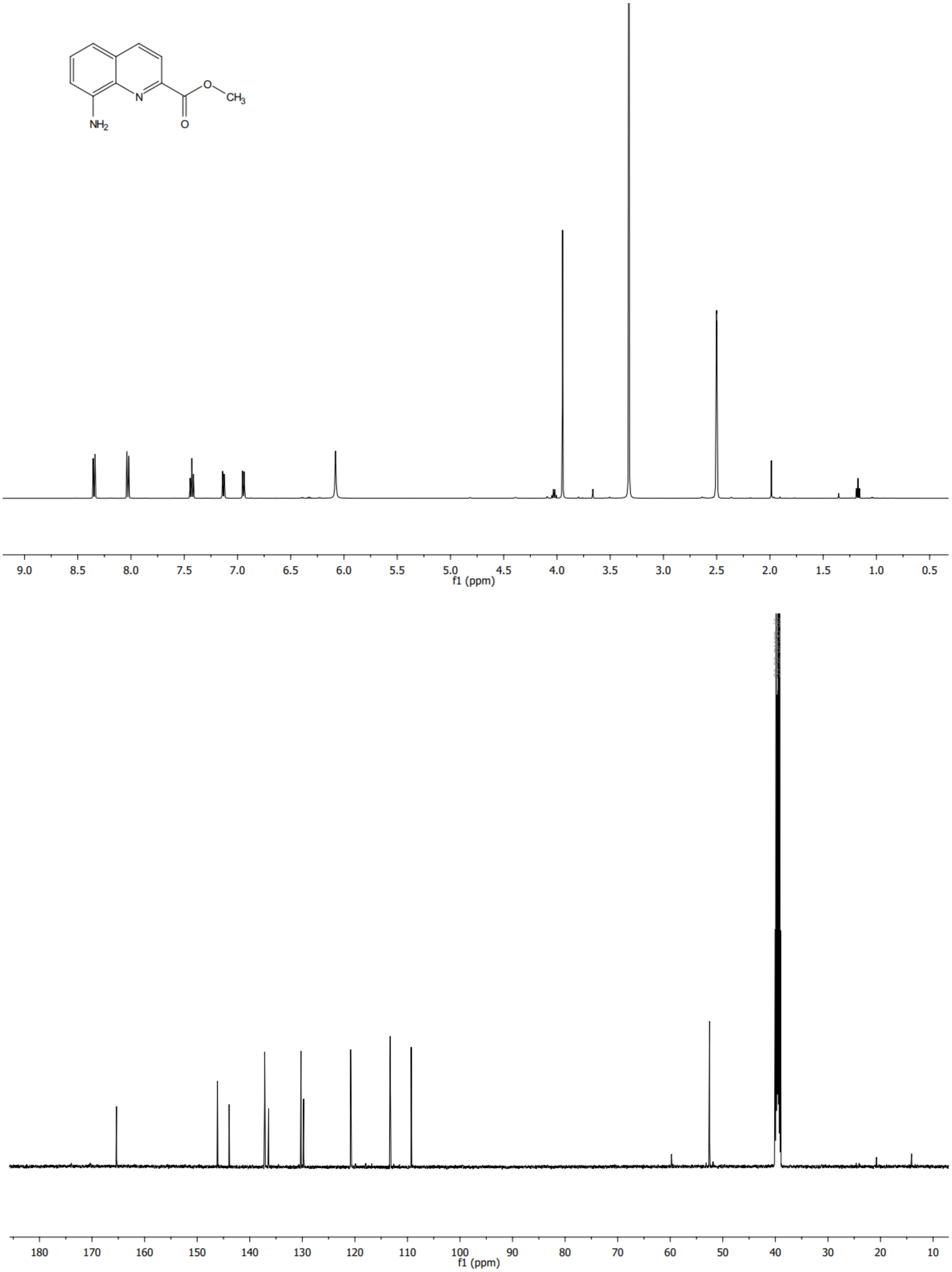

Figure S51. NMR spectra of **47**: ^{1}H NMR (500 MHz, DMSO-d_6) and ^{13}C NMR (126 MHz, DMSO-d_6).

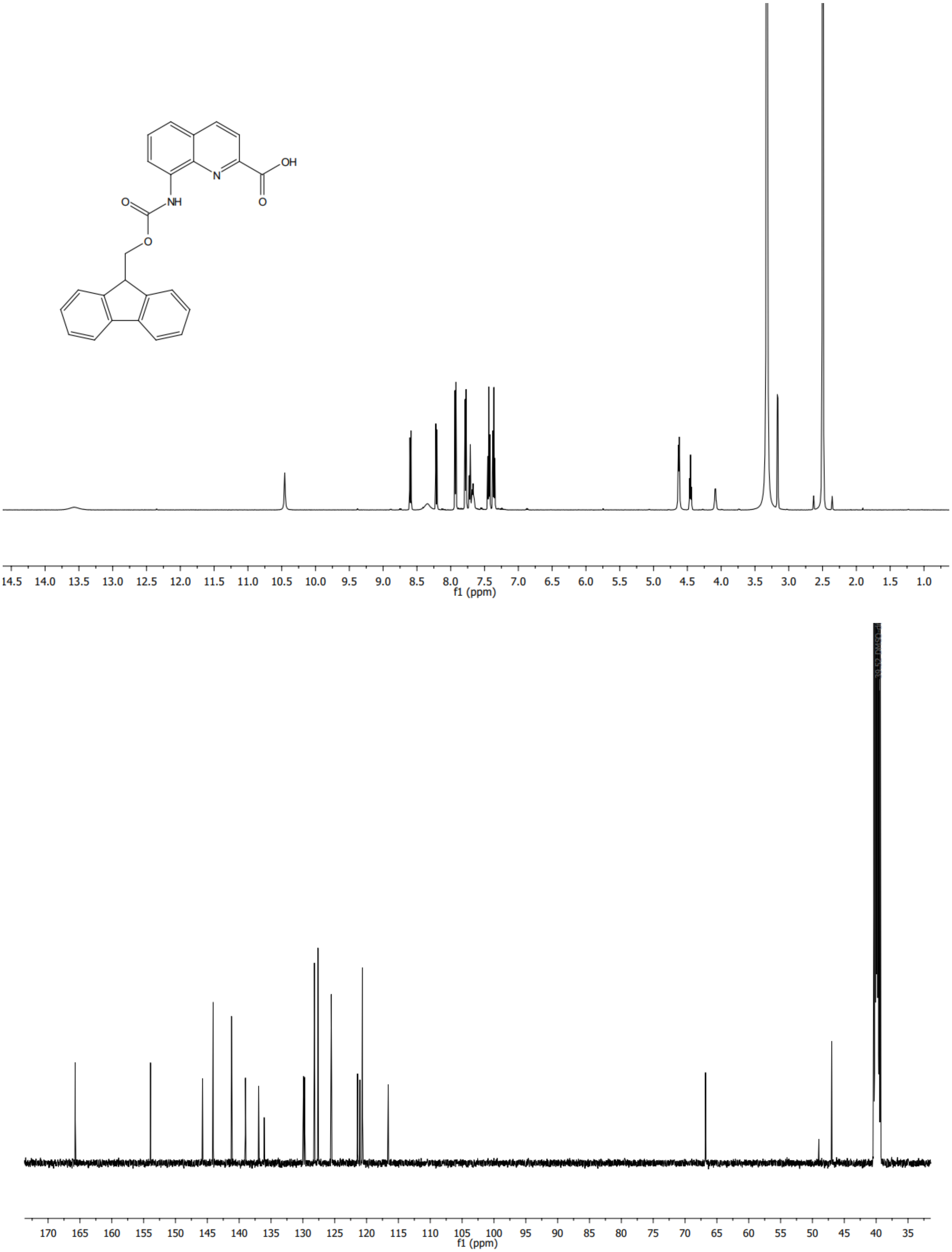

Figure S52. NMR spectra of **48**: ^{1}H NMR (500 MHz, DMSO-d_6) and ^{13}C NMR (101 MHz, DMSO-d_6)

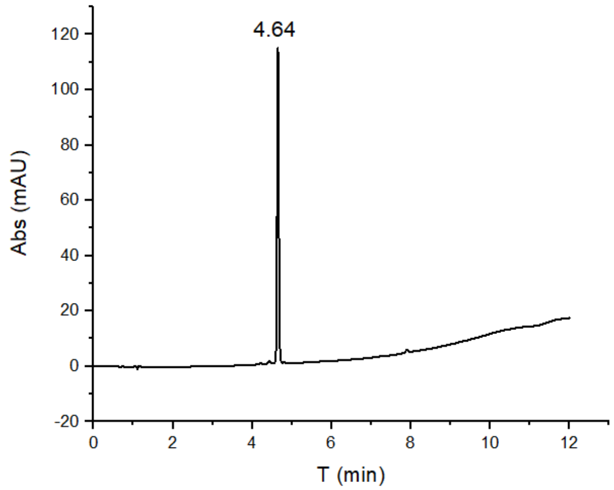

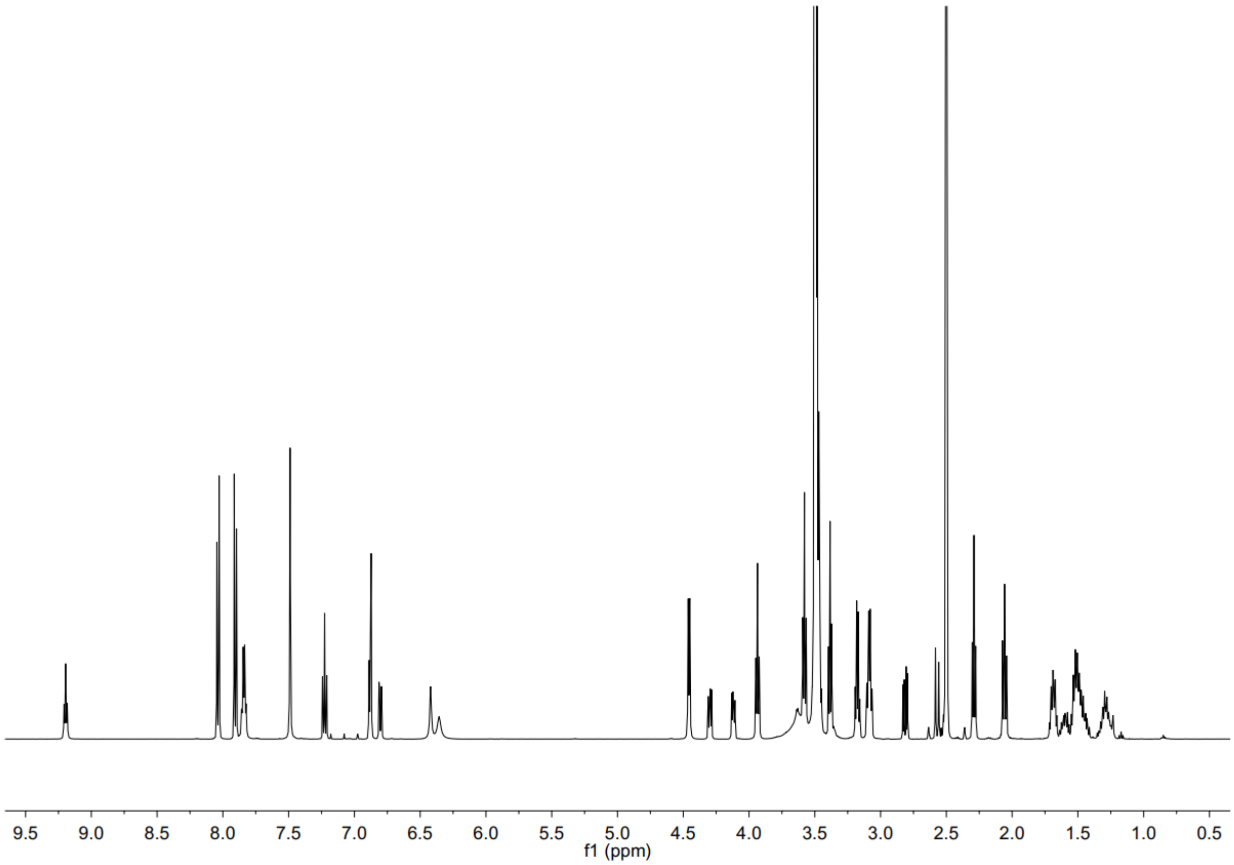

Figure S53. HPLC profile of purified compound **24** (10 to 100 B% over 10 min, 50 °C, λ = 254 nm) and ^{1}H NMR spectrum (500 MHz, DMSO-d_6, 25 °C)

6. References

1. J. Katigbak, H. Li, D. Rooklin and Y. Zhang, *J. Chem. Inf. Model.*, 2020, **60**, 1494.

2. O. Trott and A. J. Olson, *J. Comput. Chem.*, 2010, **31**, 455.

3. D. A. A. Case, H.M.; Belfon, Kellon A.A.; Ben-Shalom, Ido Y.; Berryman, Joshua T.; Brozell, Scott R.; Cerutti, David S.; Cheatham, III, Thomas E.; Cisneros, G. Andrés; Cruzeiro, Vinícius Wilian D.; Darden, Thomas A.; Duke, Robert E.; Giambaşu, George Madalin; Gilson, Michael K.; Gohlke, Holger; Götz, Andreas W.; Harris, Robert C.; Izadi, Saeed; Измайлов, Сергей Александрович; Kasavajhala, Koushik; Kaymak, Mehmet Cagri ; King, Edward; Kovalenko, Andriy F.; Kurtzman, Tom; Lee, Tai-Sung; Le Grand, Scott; Li, Pengfei; Lin, Charles; Liu, Jian; Luchko, Tyler; Luo, Ray; Machado, Matias; Man, Viet; Manathunga, Madushanka; Merz, Kenneth; Miao, Yinglong; Михайловский, Олег Владимирович; Monard, Gérald; Nguyen, Hai; O'Hearn, Kurt A.; Onufriev, Alexey; Pan, Feng; Pantano, Sergio; Qi, Ruxi; Rahnamoun, Ali; Roe, Daniel R.; Roitberg, Adrian; Sagui, Celeste; Schott-Verdugo, Stephan; Shajan, Akhil; Shen, Jana; Simmerling, Carlos; Скрынников, Николай Русланович; Smith, Jamie; Swails, Jason; Walker, Ross C.; Wang, Junmei; Wang, Jinan; Wei, Haixin; Wolf, Romain M.; Wu, Xiongwu; Xiong, Yeyue; Xue, Yi; York, Darrin; Zhao, Shiji; Kollman, Peter A., *Amber 2022*, University of California, Sanfrancisco, 2022.

4. Z. Liu, A. M. Abramyan and V. Pophristic, *J. Chem.*, 2015, **39**, 3229.

5. F. Y. Dupradeau, A. Pigache, T. Zaffran, C. Savineau, R. Lelong, N. Grivel, D. Lelong, W. Rosanski and P. Cieplak, *Phys. Chem. Chem. Phys.*, 2010, **12**, 7821.

6. J. A. Maier, C. Martinez, K. Kasavajhala, L. Wickstrom, K. E. Hauser and C. Simmerling, *J. Chem. Theory Comput.*, 2015, **11**, 3696.

7. J. Wang, R. M. Wolf, J. W. Caldwell, P. A. Kollman and D. A. Case, *J. Comput. Chem.*, 2004, **25**, 1157.

8. M. Jewginski, L. Fischer, C. Colombo, I. Huc and C. D. Mackereth, *Chembiochem.*, 2016, **17**, 727.

9. W. Kabsch, *Acta Crystallogr D Biol Crystallogr*, 2010, **66**, 125; W. Kabsch, *Acta Crystallogr. D Biol. Crystallogr.*, 2010, **66**, 133.

10. A. J. McCoy, R. W. Grosse-Kunstleve, P. D. Adams, M. D. Winn, L. C. Storoni and R. J. Read, *J. Appl. Crystallogr.*, 2007, **40**, 658.

11. P. S. Reddy, B. Langlois d'Estaintot, T. Granier, C. D. Mackereth, L. Fischer and I. Huc, *Chem. – Eur. J.*, 2019, **25**, 11042

12. L. A. Woods, O. Dolezal, B. Ren, J. H. Ryan, T. S. Peat and S.-A. Poulsen, *J. Med. Chem.*, 2016, **59**, 2192.

13. G. N. Murshudov, A.A. Vagin and E.J. Dodson, *Acta Crystallogr. D Biol. Crystallogr.*, 1997, **D53**, 240.

14. P. D. Adams, P. V. Afonine, G. Bunkoczi, V. B. Chen, I. W. Davis, N. Echols, J. J. Headd, L. W. Hung, G. J. Kapral, R. W. Grosse-Kunstleve, A. J. McCoy, N. W. Moriarty, R. Oeffner, R. J. Read, D. C. Richardson, J. S. Richardson, T. C. Terwilliger and P. H. Zwart, *Acta Crystallogr. D Biol. Crystallogr.*, 2010, **66**, 213.

15. P. Emsley, B. Lohkamp, W. G. Scott and K. Cowtan, *Acta Crystallogr. D Biol. Crystallogr.*, 2010, **66**, 486.

16. A. W. Schuttelkopf and D. M. van Aalten, Acta Crystallogr D Biol Crystallogr, 2004, 60, 1355; N. W. Moriarty, R. W. Grosse-Kunstleve and P. D. Adams, *Acta Crystallogr. D Biol. Crystallogr.*, 2009, **65**, 1074.

17. I. W. Davis, A. Leaver-Fay, V. B. Chen, J. N. Block, G. J. Kapral, X. Wang, L. W. Murray, W. B. Arendall, 3rd, J. Snoeyink, J. S. Richardson and D. C. Richardson, *Nucleic. Acids. Res.*, 2007, **35**, W375.

18. P. Koutnik, E. G. Shcherbakova, S. Gozem, M. G. Caglayan, T. Minami and P. Anzenbacher, *Chem.*, 2017, **2**, 271.

19. P. Gans, A. Sabatini and A. Vacca, Talanta, 1996, 43, 1739; P. Gans, A. Sabatini and A. Vacca, *Anal. Chim.*, 1999, **89**, 45.

20. S. Dengler, P. K. Mandal, L. Allmendinger, C. Douat and I. Huc, *Chem. Sci.*, 2021, **12**, 11004.

21. X. Hu, S. J. Dawson, P. K. Mandal, X. de Hatten, B. Baptiste and I. Huc, *Chem. Sci.*, 2017, **8**, 3741.

22. M. Vallade, P. Sai Reddy, L. Fischer and I. Huc, *Eur. J. Org. Chem.*, 2018, 5489.

23. V. Corvaglia, F. Sanchez, F. S. Menke, C. Douat and I. Huc, *Chem. – Eur. J.*, 2023, 29, e202300898.

24. J. Buratto, C. Colombo, M. Stupfel, S. J. Dawson, C. Dolain, B. Langlois d'Estaintot, L. Fischer, T. Granier, M. Laguerre, B. Gallois and I. Huc, *Angew. Chem., Int. Ed.*, 2014, **53**, 883.

25. N. G. Léonard, W. N. Palmer, M. R. Friedfeld, M. J. Bezdek and P. J. Chirik, *ACS Catal.*, 2019, **9**, 9034.

26. M. Zwillinger, P. S. Reddy, B. Wicher, P. K. Mandal, M. Csékei, L. Fischer, A. Kotschy and I. Huc, *Chem. – Eur. J.*, 2020, **26**, 17366.

27. W. P. Unsworth, J. D. Cuthbertson and R. J. K. Taylor, *Org. Lett.*, 2013, **15**, 3306.
